# Adiposity and NMR-measured lipid and metabolic biomarkers among 30,000 Mexican adults

**DOI:** 10.1038/s43856-022-00208-2

**Published:** 2022-11-14

**Authors:** Diego Aguilar-Ramirez, William G. Herrington, Jesus Alegre-Díaz, Natalie Staplin, Raúl Ramírez-Reyes, Louisa Friedrichs Gnatiuc, Michael Hill, Frederik Romer, Eirini Trichia, Fiona Bragg, Rachel Wade, Sarah Lewington, Rory Collins, Jonathan R. Emberson, Pablo Kuri-Morales, Roberto Tapia-Conyer

**Affiliations:** 1 Clinical Trial Service Unit & Epidemiological Studies Unit, Nuffield Department of Population Health, University of Oxford, Oxford, UK; 2 Faculty of Medicine, National Autonomous University of Mexico, Mexico City, Mexico; 3 MRC Population Health Research Unit, Nuffield Department of Population Health, University of Oxford, Oxford, UK

**Keywords:** Epidemiology, Metabolomics

## Abstract

**Background:**

Adiposity is a major cause of morbidity and mortality in part due to effects on blood lipids. Nuclear magnetic resonance (NMR) spectroscopy provides direct information on >130 biomarkers mostly related to blood lipid particles.

**Methods:**

Among 28,934 Mexican adults without chronic disease and not taking lipid-lowering therapy, we examine the cross-sectional relevance of body-mass index (BMI), waist circumference (WC), waist-hip ratio (WHR), and hip circumference (HC) to NMR-measured metabolic biomarkers. Confounder-adjusted associations between each adiposity measure and NMR biomarkers are estimated before and after mutual adjustment for other adiposity measures.

**Results:**

Markers of general (ie, BMI), abdominal (ie, WC and WHR) and gluteo-femoral (ie, HC) adiposity all display similar and strong associations across the NMR-platform of biomarkers, particularly for biomarkers that increase cardiometabolic risk. Higher adiposity associates with higher levels of Apolipoprotein-B (about 0.35, 0.30, 0.35, and 0.25 SD higher Apolipoprotein-B per 2-SD higher BMI, WHR, WC, and HC, respectively), higher levels of very low-density lipoprotein particles (and the cholesterol, triglycerides, and phospholipids within these lipoproteins), higher levels of all fatty acids (particularly mono-unsaturated fatty acids) and multiple changes in other metabolic biomarkers including higher levels of branched-chain amino acids and the inflammation biomarker glycoprotein acetyls. Associations for general and abdominal adiposity are fairly independent of each other but, given general and abdominal adiposity, higher gluteo-femoral adiposity is associated with a strongly favourable cardiometabolic lipid profile.

**Conclusions:**

Our results provide insight to the lipidic and metabolomic signatures of different adiposity markers in a previously understudied population where adiposity is common but lipid-lowering therapy is not.

## Plain language summary

Obesity increases the risk of multiple diseases, in part due to alterations in how the body breaks down carbohydrates and fats, which is reflected in molecules that circulate in blood. In obesity, disease risk may vary depending on whether fat accumulates in the body overall (i.e. total adiposity), in the middle of the body (i.e. abdominal adiposity) or around the hips (i.e. gluteo-femoral adiposity). Here, we show that in a population of Mexican adults higher total and abdominal adiposity relate adversely, while higher gluteo-femoral adiposity relates favourably, to numerous molecules in blood that are linked to type 2 diabetes and heart disease. These findings provide insight on the processes that link the accumulation of fat across the body with disease risk in a population where obesity rates are high.

## Abstract (web summary)

Aguilar-Ramirez et al. examine cross-sectional associations between adiposity and metabolic traits among Mexican adults. Their findings suggest that higher total and central adiposity relate adversely, whilst higher gluteo-femoral adiposity relates favourably, to numerous metabolic factors relevant to disease risk.

## Introduction

Adiposity is a leading cause of death and disability worldwide^1,2^. Prospective cohort studies have found that each 5 kg/m^2^ higher body mass index (BMI) above 25 kg/m^2^ is associated with an increase in the all-cause mortality rate of about one third^3^, chiefly because of the effects of BMI on blood pressure, diabetes and blood lipids^4^. However, although the most common and widely studied marker of adiposity, BMI does not characterise the distribution of adiposity (e.g. abdominal versus gluteo-femoral) and includes other contributors to weight (e.g. muscle). Furthermore, the extent to which the effects of different types of adiposity on mortality are driven by their impact on blood lipids are only partly understood because traditional measures of blood lipid particles tend to rely on simple clinical chemistry assays of cholesterol (either overall or that carried in particular lipid particles such as low-density lipoproteins) and triglycerides.

Nuclear magnetic resonance (NMR) spectroscopy provides a detailed quantification of circulating plasma lipoprotein lipids, including lipoprotein particle concentrations by subclasses and their lipidic composition (and provides information on other potentially relevant metabolic biomarkers). Although some studies have previously assessed the associations of BMI, and of dual-energy X-ray absorptiometry (DEXA) fat mass and visceral fat, with NMR biomarkers^5–7^, these have been largely limited to European populations and have not explored other markers of adiposity.

In Mexico, about three-quarters of adults are overweight or obese (i.e. have a body mass index >25 kg/m^2^) and lipid-lowering treatments are underused^8,9^. In this report, we use data from the Mexico City Prospective Study^10^ to explore the observational relationships between different markers of general, abdominal and gluteo-femoral adiposity with NMR lipids, lipoproteins and metabolic biomarkers among 30,000 people without diabetes or other chronic disease and not taking lipid-lowering therapy. We find that higher levels of general and abdominal markers of adiposity relate adversely to numerous molecules in blood that are linked to type 2 diabetes and heart disease. However, for a given level of general and central adiposity, we find that higher levels of gluteo-femoral adiposity relate favourably to such biomarkers.

## Methods

### Recruitment and study procedures

The rationale for, design and general aims of the Mexico City Prospective Study have been published previously^10^. Briefly, between 1998 and 2004, 52,644 men and 107,111 women aged 35 years or older who resided in two contiguous districts in Mexico City (Coyoacán and Iztapalapa) were recruited into a prospective study. Trained health professionals collected information on sociodemographic and lifestyle characteristics, medical history and current medications using standardised questionnaires. Calibrated electronic scales, stadiometers and non-stretchable tape were used to measure weight, height and waist and hip circumference, respectively. Blood pressure was measured twice with the participants seated and a 10 mL blood sample was obtained. All participants provided written informed consent. Ethics approval was granted by the Mexican Ministry of Health, the Mexican National Council of Science and Technology (0595 P-M), the Central Oxford Research Ethics Committee (C99.260) and the Ethics and Research commissions from the Medicine Faculty at the National Autonomous University of Mexico (FMED/CI/SPLR/067/2015).

### Adiposity markers

BMI was used as a proxy for general adiposity and was calculated as body weight in kilograms divided by the square of height in metres. General adiposity encompasses, but does not distinguish, the distribution of adiposity in the body. Therefore, WC (in cm) was used as a proxy of subcutaneous and visceral abdominal fat. HC (in cm) was used as a proxy of subcutaneous gluteo-femoral fat (inclusive, however, of hip muscle and bone). WHR was used as a measure of body-shape that quantifies abdominal adiposity relative to gluteo-femoral adiposity.

### Blood sample handling, storage and assays

After collection, blood samples were chilled at 4–10 °C and transported to a central laboratory for refrigeration at 4 °C and separation the next morning. Plasma and buffy coat samples were stored locally for a few months at −80 °C before being transported on dry ice to Oxford (UK) for long-term storage over liquid nitrogen at −150 °C. These storage conditions ensure stability of blood lipid and other analytes between collection and measurement^11^. In all participants, glycosylated haemoglobin (HbA_1c_) was measured from the buffy coat sample with validated high-performance liquid chromatography methods^12^. Between September 2018 and October 2019, a subset of 40,349 baseline plasma samples were subaliquoted and analysed by NMR spectroscopy. Of these, 35,380 were analysed at Nightingale Health Ltd (Kuopio, Finland) with the remaining 4969 analysed at the Clinical Trial Service Unit’s (CTSU) Wolfson Laboratory (Oxford, UK) with the same validated protocol. (Those with NMR data were predominantly from the Coyoacán study district but were otherwise similar to those without NMR data available: Table S1.) The Nightingale Health Ltd high-throughput targeted NMR-metabolomics platform^13^ quantifies 228 measures from plasma biomarkers as absolute concentrations or ratios. This automated platform has multiple and standardised quality control checkpoints at both plate and batch level. Additionally, an inter-laboratory comparison between Nightingale Health Ltd and CTSU’s Wolfson Laboratory was performed (using multiple sets of samples run in both laboratories) with good agreement observed.

### Statistical analysis

Among participants with NMR measurements available, analyses excluded those aged 85 years or older at recruitment, those who reported use of a lipid-lowering medication at recruitment, those with missing or extreme values for any anthropometry measure (Table S2) and those with missing or implausible data on covariates (see below). To reduce the risk of previous diseases distorting baseline adiposity and/or NMR biomarker levels, the main analyses further excluded those with previously-diagnosed coronary heart disease, stroke, cirrhosis, emphysema, cancer, or diabetes, as well as those with a baseline HbA_1c_ level indicative of diabetes (i.e. 6.5% or higher). Analyses including these participants are provided as sensitivity analyses however. (See Supplementary methods for a more detailed rationale for exclusion of those with diabetes, or HbA1c consistent with diabetes, from the main analyses).

**Fig. 1:**
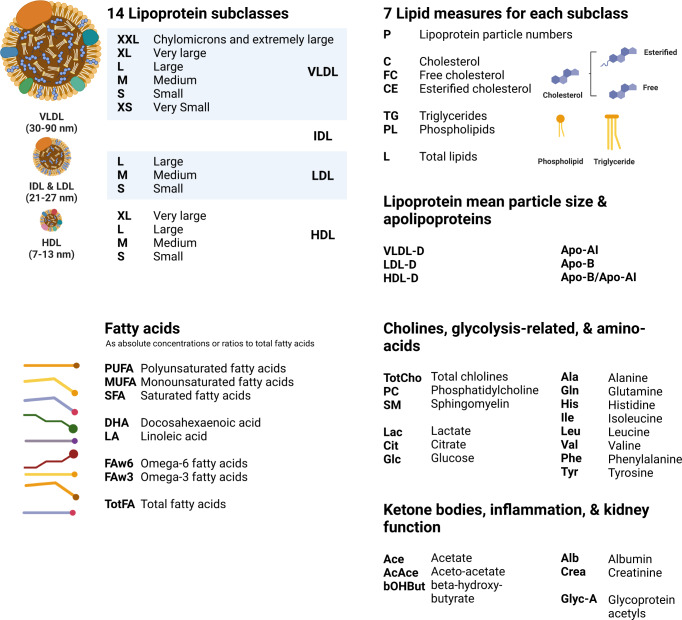
Plasma lipid and metabolic measures quantified by nuclear magnetic resonance spectroscopy. The abbreviations shown represent the nomenclature used in Figs. 2–4. Apo-AI Apolipoprotein A-I, Apo-B Apolipoprotein B-100, D Diameter, HDL High-density lipoprotein, IDL Intermediate-density lipoprotein, LDL Low-density lipoprotein, VLDL Very low-density lipoprotein. Created with BioRender.com.

For the present analysis, an array of 139 NMR biomarkers (Fig. 1 and Supplementary Data 1) that prioritised direct measurements over ratios of biomarkers was selected. The array was built by manually selecting biomarkers in such a way that all the metabolic groups resolved by the NMR platform were represented while restricting the overlap in metabolic measures (see Supplementary methods). NMR values below the detection limit were assigned the value of the lower detection limit. To facilitate comparisons between NMR biomarkers (and to facilitate comparisons with previous studies) the NMR biomarkers were then log-transformed and normalised (so that the transformed and normalised markers all had zero mean and a standard deviation of one).

Linear regression was used to individually relate BMI, WC, WHR and HC (each in SD units) to each log-NMR biomarker (also in SD units). The standardisation to SD units was done to allow appropriate comparison of associations across different adiposity measures and across different NMR biomarkers. Sex-specific SDs of adiposity values were used due to differences between men and women in the distribution of the some of the adiposity markers. Subsequent models further included a quadratic term of each adiposity marker into the regression model in order to investigate departures from linearity. However, even in the presence of non-linearity, a model which includes only a linear term still provides a useful summary statistic for how each NMR biomarker varies on average across the range of adiposity values studied. In addition, estimates of the mean level of each log-NMR biomarker were also calculated according to tenths of the distribution of each adiposity marker (by including the adiposity marker in each regression model as a 10-level categorical variable). This allows for a detailed exploration of the nature of any dose-response associations whilst making no assumptions about the nature of that association. Ten groups were selected to provide estimates across most of the distribution of each adiposity marker, whilst ensuring that each estimate was itself still statistically reliable (i.e. narrow 95% confidence limit). Such an approach affords some advantages over other approaches (e.g. use of splines) which, although providing internally-good fits to the data, offer no additional flexibility and can give misleading results where data are sparse (eg, towards the tails of the distributions).

The regression models described above were routinely adjusted for the possible confounders of age, sex, educational level, district of residence, smoking status, alcohol intake, fifths of fasting duration and NMR sample processing site (Finland or Oxford), and were used to describe the overall association of each adiposity marker to each NMR biomarker. However, adiposity markers are correlated, meaning that the overall associations of each adiposity marker in isolation may be inflated, reduced, or even reversed due to their correlations with other adiposity markers. To investigate the overall versus the independent association of each marker of adiposity to the panel of NMR biomarkers, we therefore repeated the main regression analyses after additional adjustment for other markers of adiposity. Such adjustments allow an assessment to be done of how higher or lower than expected levels of a particular adiposity marker (i.e. expected given the other adiposity markers) predict NMR levels.

To examine for potential effect modification by age and sex, additional analyses were done separately by age (those aged 35–54 versus those aged 55–84 years) and by sex. The main analyses removed people with pre-existing diseases which could lead to changes in adiposity and/or NMR biomarker levels (as inclusion of them could artificially distort the associations between adiposity and NMR biomarkers). However, in some circumstances, such exclusions may theoretically lead to the introduction of a selection bias. Sensitivity analyses were therefore done including all such participants with prior disease. In addition, to guard against potential estimation problems arising from collinearity in the mutually-adjusted regression models, additional sensitivity analyses were done utilising the residuals of each adiposity marker adjusted for other adiposity markers (rather than models in which the correlated adiposity markers were entered into the same regression model).

Regression estimates are presented with 95% confidence intervals. To account for multiplicity, however, the false discovery rate (FDR) was controlled at 5% using the Benjamini–Hochberg method^14^. Thus, for each association, a two-sided FDR-adjusted *p*-value below 0.05 was considered as evidence against the null hypothesis. For comparison of associations by age and sex, standard tests for heterogeneity were used (which were also FDR-corrected). Data processing and statistical analyses were performed in SAS 9.4 (SAS Institute, Cary NC). Plots were created in basic R (v4.0.2)^15^ and with the package “RCircos”^16^.

### Reporting summary

Further information on research design is available in the Nature Research Reporting Summary linked to this article.

## Results

Of the 159,755 potential study participants, 37,592 (24%) were excluded from the main analyses because of a prior history of diabetes or other chronic disease, because they had HbA1c ≥ 6.5% or because they were aged ≥85 years at recruitment. Of the remaining 122,163 participants, NMR-metabolomics assays were available in a subset of 31,314 (26%). Of these participants, 2232 (7%) were excluded because of missing or extreme data on adiposity, covariates or NMR biomarker data, or because, for a very small percentage, the participant was recruited twice (data from the first visit at which a blood sample was collected was used), while a further 148 (0.5%) were excluded because they were taking a lipid-lowering medication. This left 28,934 in the main analyses (10,225 men and 18,709 women; mean age 50 [SD 12] years) (Table 1 and Table S1).

**Table 1:** Baseline characteristics of 10,225 men and 18,709 women aged 35–84 included in main analyses.

	Men (*n* = 10,225)	Women (*n* = 18,709)	All (*n* = 28,934)
Age, years	50 (12)	50 (12)	50 (12)
Anthropometric and adiposity measurements			
Weight, kg	75 (12)	67 (12)	70 (13)
Height, cm	165 (7)	151 (6)	156 (9)
Body mass index, kg/m^2^	27.6 (4.0)	29.1 (5.0)	28.6 (4.7)
Waist circumference, cm	95 (10)	91 (12)	93 (11)
Hip circumference, cm	101 (7)	105 (11)	104 (10)
Waist-hip ratio	0.94 (0.06)	0.87 (0.06)	0.89 (0.07)
Socio-economic status and lifestyle characteristics			
Resident of Coyoacán	9324 (91%)	16,804 (90%)	26,128 (90%)
University/college educated	2544 (25%)	2317 (12%)	4861 (17%)
Current smoker	4686 (46%)	3912 (21%)	8598 (30%)
Current alcohol use	8080 (79%)	12,287 (66%)	20,367 (70%)
Blood pressure, mmHg			
Systolic	129 (15)	127 (17)	127 (16)
Diastolic	85 (10)	83 (10)	84 (10)
HbA_1c_	5.3 (0.4)	5.3 (0.4)	5.3 (0.4)
Fasting duration, hours			
Median (IQR)	2.8 (1.0–5.0)	2.7 (1.1–4.6)	2.7 (1.1–4.8)
<8	8816 (86%)	16,652 (89%)	25,468 (88%)

Mean (SD), median (IQR) or n (%) shown.

*HbA*_*1c*_ glycosylated haemoglobin.

Mean BMI in this main analysis population was 27.6 (SD 4.0) kg/m^2^ in men and 29.1 (SD 5.0) kg/m^2^ in women. Mean WC was higher in men than women whereas mean HC was higher in women than men. Consequently, mean WHR was higher in men than women (0.94 [0.06] vs 0.87 [0.06] units respectively). BMI was strongly correlated with WC (r = 0.82 in both men and women) and with HC (r = 0.78 in men and r = 0.86 in women), but only moderately correlated with WHR (r = 0.44 in men and r = 0.26 in women) (Table S3).

### Metabolic profiles associated with different adiposity markers

**Fig. 2:**
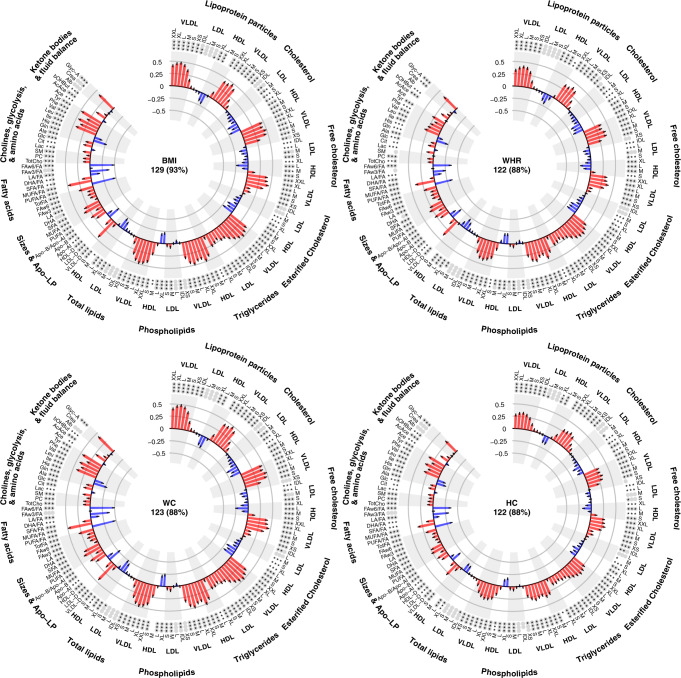
Associations of adiposity measures with each NMR biomarker. Difference (in SD) units of each log-NMR biomarker associated with 2 SD higher adiposity measures. Analyses include 28,934 participants aged 35–84 years without diabetes or other chronic disease and who were not using lipid-lowering medications at baseline. All linear regression models are adjusted for age, sex, educational level, district of residence, smoking, alcohol intake, fasting duration and NMR − experiment site. 2-SD differences represent about 9 kg/m^2^ BMI, 0.12 units waist-hip ratio, 22 cms waist circumference and 18 cms hip circumference. The error bars represent 95% confidence intervals. The n reported corresponds to the number of biomarkers for which the statistical association of the linear adiposity term (after false discovery rate correction) gives *p* < 0.05. The number of * symbols identifies the level of significance: *p* < 0.05 (*), *p* < 0.01 (**) or *p* < 0.001 (***). Similarly, the statistical significance of an additional quadratic adiposity term is shown by the number of filled grey circles: *p* < 0.05 (one grey circle), *p* < 0.01 (two grey circles) or *p* < 0.001 (three grey circles). AcAce acetoacetate, Ace acetate, Ala alanine, Alb albumin, Apo−A1 apolipoprotein A1, Apo−B apolipoprotein-B, bOHBut 3−hydroxybutyrate, BMI body mass index, Cit citrate, Crea creatinine, DHA docosahexaenoic acid, FA fatty acids, FAw3 omega−3 fatty acids, FAw6 omega−6 fatty acids, Glc glucose, Gln glutamine, Glyc−A glycoprotein acetyls, HC hip circumference, HDL high density lipoproteins, HDL − D high density lipoprotein particle diameter, His histidine, IDL intermediate density lipoproteins, Ile isoleucine, L large, LA linoleic acid, Lac lactate, LDL low-density lipoproteins, LDL − D low-density lipoprotein particle diameter, Leu leucine, LP lipoprotein, M medium, MUFA mono-unsaturated fatty acids, PC phosphatidylcholines, Phe phenylalanine, PUFA polyunsaturated fatty acids, S small, SFA saturated fatty acids, SM sphingomyelins, TotFA total fatty acids, TotCho total cholines, Tyr tyrosine, Val valine, VLDL very low-density lipoproteins, VLDL − D very low-density lipoprotein particle diameter, WC waist circumference, WHR waist-hip ratio, XL very large, XS very small, XXL extremely large.

Figure 2 shows the associations between markers of general (BMI), abdominal (WHR and WC) and gluteo-femoral (HC) adiposity and each NMR biomarker, which are circumferentially sorted according to metabolic groups (Supplementary Data 2). Estimates denote the adjusted mean difference in SD units of each log-NMR biomarker per 2-SD higher adiposity marker, equivalent to about 9 kg/m^2^ of BMI, 0.13 units of WHR, 21 cm of WC and 18 cm of HC. Overall, the metabolic profiles of all adiposity markers were extremely consistent: of the 139 biomarkers used for analyses, BMI was associated with 129 (93%), WHR with 122 (88%), WC with 123 (88%) and HC with 122 (88%) at the FDR-adjusted significance threshold.

#### ApoB-carrying lipoproteins and their lipids

Higher levels of adiposity were associated with higher levels of the lipids within (i.e. triglycerides, cholesterol and phospholipids) and the particle numbers of all VLDL subclasses. Similarly, higher levels of adiposity were associated with higher triglycerides within all lipoproteins (VLDLs, IDLs, LDLs and HDLs). However, higher adiposity levels were not much associated with IDL and LDL lipids and lipoproteins. Consequently, higher adiposity levels were associated with higher ApoB (about 0.20, 0.15, 0.20 and 0.15 SD higher ApoB per 2-SD higher BMI, WHR, WC and HC, respectively) and with larger VLDL and LDL mean diameters.

#### HDL-related biomarkers

The associations of adiposity with HDL biomarkers varied by type of lipid and by lipoprotein subclass. Higher adiposity levels were inversely associated with total and esterified cholesterol in all HDL subclasses. In the two largest HDL subclasses, higher adiposity levels tended to be associated with lower levels of large HDL particle numbers, free cholesterol and phospholipids. However, in the two smallest HDL subclasses (and especially the smallest HDL subclass), the opposite was observed: higher adiposity levels were associated with higher levels of small HDL particle numbers, free cholesterol and phospholipids. Considering all HDL subclasses together, higher adiposity was associated with slightly lower ApoA1 levels and moderately lower HDL diameter.

#### Fatty acids

Higher adiposity levels were associated with higher levels of all fatty acid measures (as absolute concentrations), of which the strongest association was observed for MUFA. Relative to total fatty acids, adiposity was associated with a fatty acid profile with proportionally higher MUFA, SFA and omega-3, but proportionally lower PUFA, DHA, LA and omega-6. The strongest positive associations were for the ratio of MUFA to total fatty acids (about 0.50, 0.40, 0.50 and 0.30 SD higher biomarker levels per 2-SD higher BMI, WHR, WC and HC, respectively). Conversely, the strongest inverse associations were for ratio of omega-6 to total fatty acids (about 0.50, 0.35, 0.50 and 0.35 SD lower biomarker levels per 2-SD higher BMI, WHR, WC and HC, respectively).

#### Cholines, glycolysis metabolites, amino acids, ketone bodies and other

Higher adiposity levels were associated with moderately higher levels of cholines, lactate, glucose, alanine and the ketone body acetoacetate. Furthermore, adiposity was also positively associated with higher levels of the branched-chain amino acids isoleucine, leucine and valine, the aromatic amino acids phenylalanine and tyrosine, as well as the inflammation marker glycoprotein acetyls (about 0.40, 0.30, 0.40 and 0.30 SD higher Glyc-A levels per 2-SD higher BMI, WHR, WC and HC, respectively). Adiposity levels were inversely associated with glutamine and histidine.

#### Shape of the relationships between adiposity markers and NMR biomarkers

Most of the biomarkers which varied appreciably depending on adiposity did so in a curvilinear fashion (Figs. S1–S4). Indeed, the addition of a quadratic adiposity term to the main regression models significantly improved model fit for 92% of the BMI associations, 87% of the WHR associations, 90% of the WC associations and 89% of the HC associations (Fig. 2). Thus, the main linear associations reported above correspond only to the average associations seen across the adiposity distributions studied (but may not be a good reflection of the associations seen towards the tails of each adiposity distribution).

#### Associations by age and sex

Overall, associations of adiposity markers with VLDL particles and lipids, with triglycerides, with certain fatty acids and with Glyc-A tended to be stronger in those aged 35–54 years than in those 55–84 years (Fig. S5). Likewise, the associations of certain VLDL particles and lipids, of triglycerides, and most fatty acids were somewhat stronger in men than in women (Fig. S6). Most associations did not qualitatively vary by age or sex. (Except for certain LDL-related biomarkers, for which associations were very close to the null).

### Effect of adjusting for other adiposity markers

**Fig. 3:**
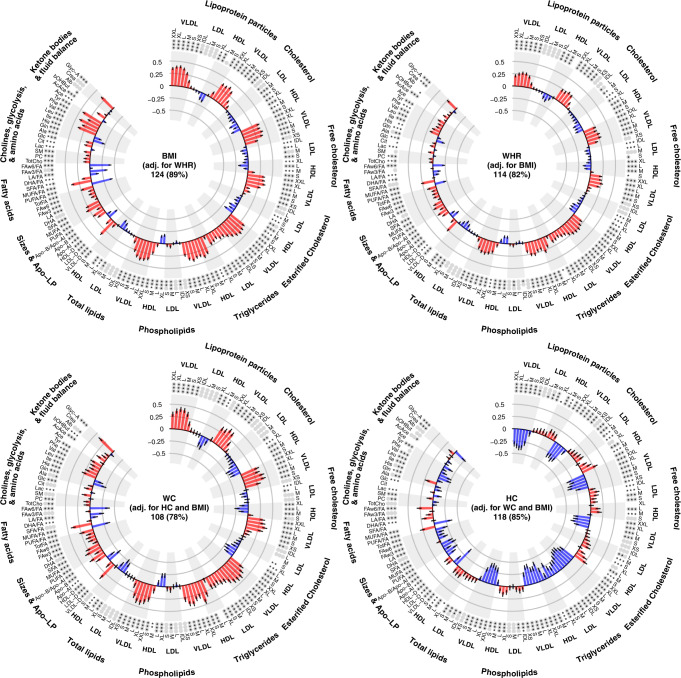
Associations of (mutually − adjusted) adiposity measures with each NMR biomarker. Difference (in SD) units of each log-NMR biomarker associated with 2 SD higher adiposity measures. Analyses, exclusions, notation and abbreviations as in Fig. 2.

**Fig. 4:**
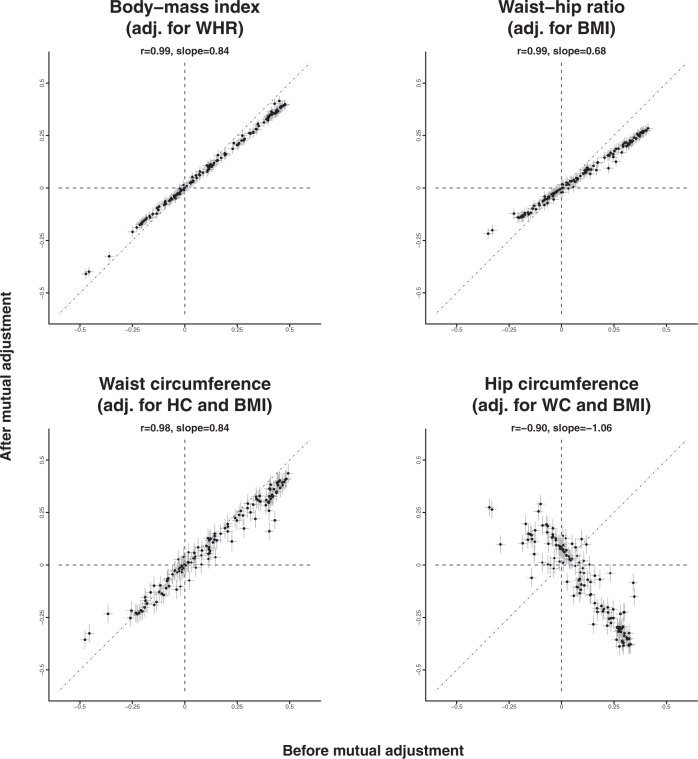
Associations of each adiposity marker with NMR biomarkers before and after adjustment for other adiposity measures. Difference (in SD) units of each log-NMR biomarker associated with 2 SD higher adiposity measures. Analyses and exclusions as in Fig. 2. Vertical and horizontal bars represent 95% confidence intervals.

Figure 3 shows the associations of BMI (adjusted for WHR), of WHR (adjusted for BMI), of WC (adjusted for HC and BMI) and of HC (adjusted for WC and BMI) with each NMR biomarker (Supplementary Data 2). The extent of agreement between associations before and after mutual adjustment is shown in Fig. 4. For the markers of general and abdominal adiposity, mutual adjustment for the other adiposity markers attenuated the associations seen with the various NMR biomarkers but the general patterns of associations remained. However, for gluteo-femoral adiposity (HC), adjustment for markers of abdominal (WC) and general (BMI) adiposity had a substantial impact, reversing the direction of the association that was seen before adjustment for the majority of NMR biomarkers. For example, given (ie, adjusted for) WC and BMI, higher levels of HC were associated with lower levels of VLDL lipids and lipoproteins, smaller VLDLs, lower ApoB, lower triglycerides, lower fatty acids and amino acids and lower Glyc-A.

### Sensitivity analyses

The main results were broadly comparable when repeated after inclusion of participants with diabetes or other chronic disease (Figs. S7–S9) or when based on the residuals of each adiposity marker adjusted for other adiposity markers (rather than entering multiple adiposity markers simultaneously into a regression model: Fig. S10).

## Discussion

This study of 28,934 adults without diabetes or other chronic disease and not taking lipid-lowering treatments when recruited into the Mexico City Prospective Study compared the associations of markers of general (BMI), abdominal (WC, WHR) and gluteo-femoral adiposity (HC) with NMR-measured metabolic biomarkers. Results show that these adiposity markers associate with a metabolic profile that includes alterations in biomarkers linked to higher risk of type 2 diabetes^17–19^, and of cardiovascular disease^20,21^. The metabolic profile associated with adiposity did not vary much by age or sex, albeit associations (positive or negative) tended to be stronger at younger than at older ages and stronger in men than in women. This study also shows that while general and abdominal adiposity are similarly associated with NMR metabolic biomarkers, and that these associations are largely independent of each other, the associations of gluteo-femoral adiposity with NMR biomarkers are reversed after adjustment for waist circumference and BMI. Thus, for a given level of general and abdominal adiposity, fat (and muscle) mass preferentially stored around the hips was associated with a more favourable metabolic profile.

There is limited population-based evidence of the associations of adiposity markers with lipids from Mexico. A previous sub-study of a Mexican nationally-representative health survey (of about 2200 participants from the 44,000 survey sample) from the year 2000 found that obesity (i.e. BMI ≥ 30 kg/m^2^) was strongly associated with hypercholesterolaemia (defined as total cholesterol above 200 mg/dL) and hypertriglyceridemia (defined as triglycerides above 150 mg/dL), and that 93% of those with obesity had at least one lipid abnormality (i.e. elevated triglycerides or total cholesterol, or decreased HDL cholesterol)^22^. The NMR metabolic profiling from the current study of ~30,000 Mexican adults greatly expands these observations by showing that these lipid perturbations are due to a diverse range of alterations across lipoprotein subclasses. Adiposity-related hypertriglyceridemia is mainly driven by increased numbers of triglyceride-rich VLDLs (which carry the largest proportion of triglycerides in blood). Concurrently, the cholesterol in these lipoproteins also seem to be higher at higher adiposity levels. Although LDL-related measures (except for the triglycerides within LDL particles) did not vary much with adiposity, VLDL-related measures did. Consequently, since LDL and VLDL both have exactly one ApoB anchored to their phospholipidic surface, higher adiposity was moderately strongly related to higher ApoB. HDL alterations related to adiposity varied by subclass size and resulted in only modest increases in ApoA1. Adiposity was also associated with changes in fatty acids, cholines, glycolysis-related metabolites, amino acids, ketone bodies and inflammation. Importantly, the metabolic profile of adiposity described in this study largely aligns with results derived from Finnish^5^, British^6^ and North American^7^ populations, so the presented results suggest that the correlations of adiposity with the NMR metabolic biomarkers are broadly consistent across Western and Latin-American populations.

The findings from this study offer mechanistic insights on the links between adiposity and cardiometabolic and other diseases. Alterations in ApoB-carrying VLDLs, ApoB, triglycerides and the inflammation marker Glyc-A link adiposity to an inflammed atherogenic pattern which predisposes to atherothrombotic cardiovascular disease^23–25^. Of these, ApoB, VLDL-triglycerides and VLDL particles have been causally linked to coronary artery disease either by randomised trial^26^ or genetic evidence^27,28^. Higher levels of VLDLs and triglycerides, fatty acids, branched-chain amino acids, glucose and ketone bodies have also been linked to incident insulin resistance and type 2 diabetes^17–19,29,30^. Of these, the branched-chain amino acids leucine, isoleucine and valine may be causally linked to type 2 diabetes^31^. Certain metabolic biomarkers involving lipids, fatty acids and Glyc-A have been recently associated with higher risk of severe pneumonia and COVID-19^32^. Although causality is yet to be determined, it is possible that some of these biomarkers mediate the associations between excess adiposity and higher risk of death from respiratory infection. Metabolic profiling with targeted NMR metabolomics could have translational applications for risk assessment in people with overweight or obesity.

An important finding of this study is that the associations of abdominal adiposity (WC and WHR) with NMR biomarkers were nearly identical to those of general adiposity (BMI). These findings are consistent with a previous NMR-metabolomics study^6^. The present findings further showed that the metabolic signatures of general and abdominal adiposity remain largely unchanged (albeit with attenuated strength) after mutual adjustment, supporting previous observations of how general and abdominal adiposity strongly and independently associate with cardiometabolic mortality^33^.

Conversely, the metabolic profile of gluteo-femoral adiposity, after adjustment for BMI and waist circumference, was reversed, showing that the risk profile of higher hip circumference independently of other adiposity markers is towards lower cardiometabolic risk. Thus, although knowledge of height, weight and waist circumference may provide a reasonable prediction of hip circumference (accounting for almost three-quarters of its variance), the difference between actual and predicted hip circumference was associated with lower levels of atherosclerotic lipids and lipoproteins and of chronic inflammation (and other fluctuations in NMR biomarkers). These findings greatly expand previous results from the EPIC-Norfolk study^34^ in which higher levels of HC were cross-sectionally associated with lower levels of total and LDL-cholesterol and with higher levels of HDL cholesterol when adjusting for BMI or waist circumference. This low-cardiometabolic-risk profile may explain recent results from the MCPS that showed how HC (adjusted for BMI and WC) is strongly associated with lower risk of vascular-metabolic mortality^35^.

Assuming causation, the implications of our findings are that general, abdominal and gluteo-femoral adiposity are likely to exert their effects (of risk or protection) on cardiometabolic diseases through similar metabolic pathways (albeit in opposite direction for gluteo-femoral adiposity). Further analyses using genetic data are needed to formally assess the causal relevance of these associations at a population level and help confirm whether or not fat (or muscle) stored preferentially around the hips really results in a causally favourable metabolic profile^36^.

To the best of our knowledge, this large study is the first population-based assessment of the associations of adiposity markers with NMR-measured lipids, lipoproteins and other metabolic biomarkers in a Mexican population. It benefits from its large scale, use of standardised adiposity measurements and low use of lipid-lowering drugs in the population at the time of recruitment. While the main analyses were limited to healthy participants (to try to reduce reverse causality bias), results from sensitivity analyses including those with prior disease were similar, suggesting that selection bias was unlikely to have had a major effect. Potential limitations, however, include the absence of imaging to measure fat in the abdomen (or elsewhere in the body) and lean mass, which would allow the comparison of these body composition measures alongside traditionally markers of adiposity, and the observational cross-sectional nature of these analyses. Additionally, the interactions by age in the associations of adiposity and LDL biomarkers (unlikely to be caused by lipid-lowering indication in those at higher adiposity levels as these individuals were excluded) require further exploration. Triangulation and integration of findings from multiple study designs, including Mendelian randomisation approaches, are likely to be needed to establish which of the associations in our report are truly causal (as done recently for some BMI-metabolic associations using Finnish data)^5^. Although derived from adults living in Mexico City, the findings are likely to be generalisable to other populations in Mexico (and other parts of Latin-America). Future assessments in other populations would be valuable however.

In summary, this study of middle-aged Mexican adults without diabetes shows that general and abdominal adiposity are strongly, similarly, and independently associated with a metabolic profile of high cardiometabolic risk. This metabolic profile associated with higher levels of ApoB and ApoB-carrying lipoproteins, but particularly VLDLs; with heterogeneous and subclass-specific alterations in HDL; and with higher MUFAs, branched-chain and aromatic amino acids and inflammation. Given general and abdominal adiposity however, higher levels of hip circumference were strongly associated with a favourable metabolomics profile.

## Supplementary information

Supplementary Data 1

Supplementary Data 2

Supplementary information

Description of Additional Supplementary Files

Reporting Summary

## Data Availability

The MCPS represents a long-standing collaboration between researchers at the National Autonomous University of Mexico (UNAM) and the University of Oxford. The investigators welcome requests from researchers in Mexico and elsewhere who wish to access MCPS data. If you are interested in obtaining data from the study for research purposes, or in collaborating with MCPS investigators on a specific research proposal, please visit https://www.ctsu.ox.ac.uk/research/prospective-blood-based-study-of-150-000-individuals-in-mexico where you can download the study’s Data and Sample Access Policy in English or Spanish. The policy lists the data available for sharing with researchers in Mexico and in other parts of the world. Full details of the data available may also be viewed at https://datashare.ndph.ox.ac.uk/. The NMR data used in the current report were made available to researchers from Mexico in October 2022. Source data for the figures can be found in Supplementary Data 2.

## References

[1] Gakidou E (2017). Global, regional, and national comparative risk assessment of 84 behavioural, environmental and occupational, and metabolic risks or clusters of risks, 1990–2016: a systematic analysis for the Global Burden of Disease Study 2016. Lancet.

[2] The GBD. (2017). 2015 Obesity Collaborators. Health Effects of Overweight and Obesity in 195 Countries over 25 Years. N. Engl. J. Med..

[3] Prospective Studies Collaboration. (2009). Body-mass index and cause-specific mortality in 900 000 adults: collaborative analyses of 57 prospective studies. Lancet.

[4] Wade KH (2018). Assessing the Causal Role of Body Mass Index on Cardiovascular Health in Young Adults: Mendelian Randomization and Recall-by-Genotype Analyses. Circulation.

[5] Würtz P (2014). Metabolic signatures of adiposity in young adults: mendelian randomization analysis and effects of weight change. Sheehan NA, editor. PLoS Med.

[6] Bell JA (2018). Associations of body mass and fat indexes with cardiometabolic traits. J. Am. Coll. Cardiol..

[7] Neeland, I. J. et al. Metabolomics profiling of visceral adipose tissue: results from MESA and the NEO study. *JAHA* https://www.ahajournals.org/doi/10.1161/JAHA.118.010810 (2019). 10.1161/JAHA.118.010810 PMC6512086 31017036

[8] Barquera S (2020). Obesidad en México, prevalencia y tendencias en adultos. Ensanut 2018-19. Salud Publica Mex.

[9] Aguilar-Ramirez D (2021). Changes in the diagnosis and management of diabetes in mexico city between 1998–2004 and 2015–2019. Diabetes Care..

[10] Tapia-Conyer R (2006). Cohort profile: the Mexico City Prospective Study. Int. J. Epidemiol.

[11] Muzakova V, Beekhof PK, Jansen EHJM (2020). Very long-term stability of lipid biomarkers in human serum. Anal. Biochem..

[12] Youngman LD, Clark S, Manley S, Peto R, Collins R (2002). Reliable measurement of glycated hemoglobin in frozen blood samples: implications for epidemiologic studies. Clin. Chem..

[13] Soininen P, Kangas AJ, Würtz P, Suna T, Ala-Korpela M (2015). Quantitative serum nuclear magnetic resonance metabolomics in cardiovascular epidemiology and genetics. Circ. Cardiovasc. Genet..

[14] Benjamini Y, Hochberg Y (1995). Controlling the false discovery rate: a practical and powerful approach to multiple testing. J. R. Stat. Soc. Ser. B Methodol..

[15] R Core Team. *R: A language and environment for statistical computing*. (R Foundation for Statistical Computing, Vienna, 2021).

[16] Zhang H, Meltzer P, Davis S (2013). RCircos: an R package for Circos 2D track plots. BMC Bioinforma..

[17] Ahola-Olli AV (2019). Circulating metabolites and the risk of type 2 diabetes: a prospective study of 11,896 young adults from four Finnish cohorts. Diabetologia..

[18] Bell JA (2020). Early metabolic features of genetic liability to type 2 diabetes: cohort study with repeated metabolomics across early life. Diabetes Care.

[19] Wang TJ (2011). Metabolite profiles and the risk of developing diabetes. Nat. Med..

[20] Würtz P (2015). Metabolite profiling and cardiovascular event risk: a prospective study of 3 population-based cohorts. Circulation..

[21] Holmes MV (2018). Lipids, lipoproteins, and metabolites and risk of myocardial infarction and stroke. J. Am. Coll. Cardiol..

[22] Barquera S (2007). Dyslipidemias and obesity in Mexico. Salud pública Méx.

[23] Després JP, Lemieux I (2006). Abdominal obesity and metabolic syndrome. Nature.

[24] Libby P (2019). Atherosclerosis. Nat. Rev. Dis. Primers.

[25] Ridker PM (2017). Antiinflammatory therapy with canakinumab for atherosclerotic disease. N. Engl. J. Med..

[26] Bhatt DL (2019). Cardiovascular risk reduction with icosapent ethyl for hypertriglyceridemia. N. Engl. J. Med..

[27] Holmes MV (2015). Mendelian randomization of blood lipids for coronary heart disease. Eur. Heart J..

[28] Richardson TG (2020). Evaluating the relationship between circulating lipoprotein lipids and apolipoproteins with risk of coronary heart disease: a multivariable Mendelian randomisation analysis. Rader DJ, editor. PLoS Med.

[29] Guasch-Ferré M (2016). Metabolomics in prediabetes and diabetes: a systematic review and meta-analysis. Diabetes Care.

[30] Liu J (2017). A Mendelian Randomization study of metabolite profiles, fasting glucose, and type 2 diabetes. Diabetes..

[31] Wang Q, Holmes MV, Davey Smith G, Ala-Korpela M (2017). Genetic support for a causal role of insulin resistance on circulating branched-chain amino acids and inflammation. Diabetes Care.

[32] Julkunen H, Cichońska A, Slagboom PE, Würtz P (2021). Nightingale Health UK Biobank Initiative. Metabolic biomarker profiling for identification of susceptibility to severe pneumonia and COVID-19 in the general population. eLife..

[33] Gnatiuc L (2019). General and abdominal adiposity and mortality in Mexico City: a prospective study of 150 000 adults. Ann. Intern. Med..

[34] Canoy D (2006). Serum lipid concentration in relation to anthropometric indices of central and peripheral fat distribution in 20,021 British men and women: results from the EPIC-Norfolk population-based cohort study. Atherosclerosis.

[35] Gnatiuc L (2022). Abdominal and gluteo-femoral markers of adiposity and risk of vascular-metabolic mortality in a prospective study of 150 000 Mexican adults. Eur. J. Prev. Cardiol.

[36] Karpe F, Pinnick KE (2015). Biology of upper-body and lower-body adipose tissue—link to whole-body phenotypes. Nat. Rev. Endocrinol..

