# Supplementary material for: Adiposity and NMR-measured lipid and metabolic biomarkers among 30,000 Mexican adults
Source: Commun Med (Lond). 2022 Nov 14;2:143. doi: 10.1038/s43856-022-00208-2 (PMC9663185; doi:10.1038/s43856-022-00208-2)
Supplement: Supplementary file 3 — Supplementary information [file 43856_2022_208_MOESM3_ESM.pdf]

Adiposity and NMR-measured lipid and metabolic biomarkers among 30,000 Mexican adults

Supplementary Information

Supplementary Tables

	Page
S1. Baseline characteristics of those in the main versus the sensitivity analyses, as well as all those aged 35 to 84 years with versus without NMR data available	2
S2. Exclusion criteria for extreme measures of anthropometry or adiposity	3
S3. Correlations between adiposity markers (for 28,934 participants in the main analysis)	4

Supplementary Figures

S1. NMR biomarkers by body−mass index levels before and after adjustment for WHR	5
S2. NMR biomarkers by waist−hip ratio levels before and after adjustment for BMI	12
S3. NMR biomarkers by waist circumference levels before and after adjustment for HC and BMI	19
S4. NMR biomarkers by hip circumference levels before and after adjustment for WC and BMI	26
S5. Comparison of associations of adiposity measures with each NMR biomarker by age	33
S6. Comparison of associations of adiposity measures with each NMR biomarker by sex	34

Sensitivity analyses *including* those with diabetes or other chronic disease

S7. Associations of adiposity measures with each NMR biomarker	35
S8. Comparison of associations of adiposity measures with each NMR biomarker by age	36
S9. Comparison of associations of adiposity measures with each NMR biomarker by sex	37
S10. Independent associations of adiposity measures with each NMR biomarker in the main analysis population using residuals of each adiposity measure rather than mutual adjustments	38

Supplementary Methods

Supplementary Methods 39

Table S1. Baseline characteristics of those in the main *versus* the sensitivity analyses, as well as all those aged 35 to 84 years with *versus* without NMR data available

	**Main analysis population* (*n*=28,934)**	**Sensitivity analysis population† (*n*=36,481)**	**Participants aged 35-84 years with NMR data (*n*=39,762)**	**Participants aged 35-84 years without NMR data (*n*=117,527)**
Age, years	50 (12)	52 (12)	52(12)	52 (13)
Men	10,225 (35%)	12,839 (35%)	14,407 (36%)	37,422 (32%)
Anthropometric and adiposity measurements				
Weight, kg	70 (13)	70 (13)	70 (13)	71 (13)
Height, cm	156 (9)	156 (9)	156 (9)	156 (9)
Body-mass index, kg/m^2	28.6 (4.7)	28.7 (4.8)	28.7 (4.9)	29.2 (5.1)
Waist circumference, cm	93 (11)	93 (11)	93 (12)	95 (12)
Hip circumference, cm	104 (10)	104 (10)	104 (10)	105 (11)
Waist-hip ratio	0.89 (0.07)	0.90 (0.07)	0.90 (0.08)	0.90 (0.08)
Socio-economic status and lifestyle characteristics				
Resident of Coyoacán	26,128 (90%)	32,849 (90%)	35,624 (90%)	27,326 (23%)
University/college educated	4861 (17%)	5467 (15%)	5,995 (15%)	18,347 (16%)
Current smoker	8598 (30%)	10,332 (28%)	11,355 (29%)	31,889 (27%)
Current alcohol use	20,367 (70%)	24,832 (68%)	27,172 (68%)	76,716 (65%)
Blood pressure, mmHg				
Systolic	127 (16)	129 (17)	129 (17)	127 (17)
Diastolic	84 (10)	84 (10)	84 (10)	83 (10)
HbA1c	5.3 (0.4)	5.9 (1.7)	5.9 (1.7)	6.2 (1.7)
Fasting duration, hours	2.7 (1.1-4.8)	2.8 (1.1-4.8)	2.8 (1.1-4.8)	2.6 (1.1-4.5)
<8 hours	25,468 (88%)	32,236 (88%)	34,835 (88%)	98,342 (84%)
Previously-diagnosed diseases				
Diabetes‡	0 (0.0%)	4,499 (12%)	5,036 (13%)	16,827 (14%)
Cardiovascular disease	0 (0.0%)	1,028 (3%)	1,177 (3%)	2,935 (2%)
Other§	0 (0.0%)	1,036 (3%)	1,189 (3%)	2,746 (2%)

Mean (SD), median (IQR), or n (%) shown.

* Aged 35-84 years, no previously-diagnosed diabetes or other chronic disease, and complete data.

† As for main analysis population but *including* those with prior chronic disease or HbA1c >6.5%

‡ Previously-diagnosed or treated.

§ Cirrhosis, emphysema or cancer.

Table S2. Exclusion criteria for extreme measures of anthropometry or adiposity

	All participants aged 35-84 (*n*=157,289)
**Anthropometry**	
Height <120 or >200 cm	99 (<0.5%)
Weight <35 or >250 kg	66 (<0.5%)
Waist circumference <60 or >180 cm	195 (<0.5%)
Hip circumference <70 or >180 cm	157 (<0.5%)
**Adiposity**	
Body-mass index <15 or >60 kg/m^2	55 (<0.5%)
Waist-hip ratio <0.5 or >1.5	54 (<0.5%)

Table S3. Correlations between adiposity markers (for 28,934 participants in the main analysis)

	BMI	WC	HC	WHR	
BMI	1.00	Men			
	1.00	Women			
WC	0.82	1.00	Men		
	0.82	1.00	Women		
HC	0.78	0.81	1.00	Men	
	0.86	0.82	1.00	Women	
WHR	0.44	0.71	0.17	1.00	Men
	0.26	0.63	0.07	1.00	Women

Pearson correlation coefficients shown. BMI=Body mass index, HC=Hip circumference, WC=Waist circumference, WHR=Waist-hip ratio

Figure S1. NMR biomarkers by body−mass index levels before and after adjustment for WHR

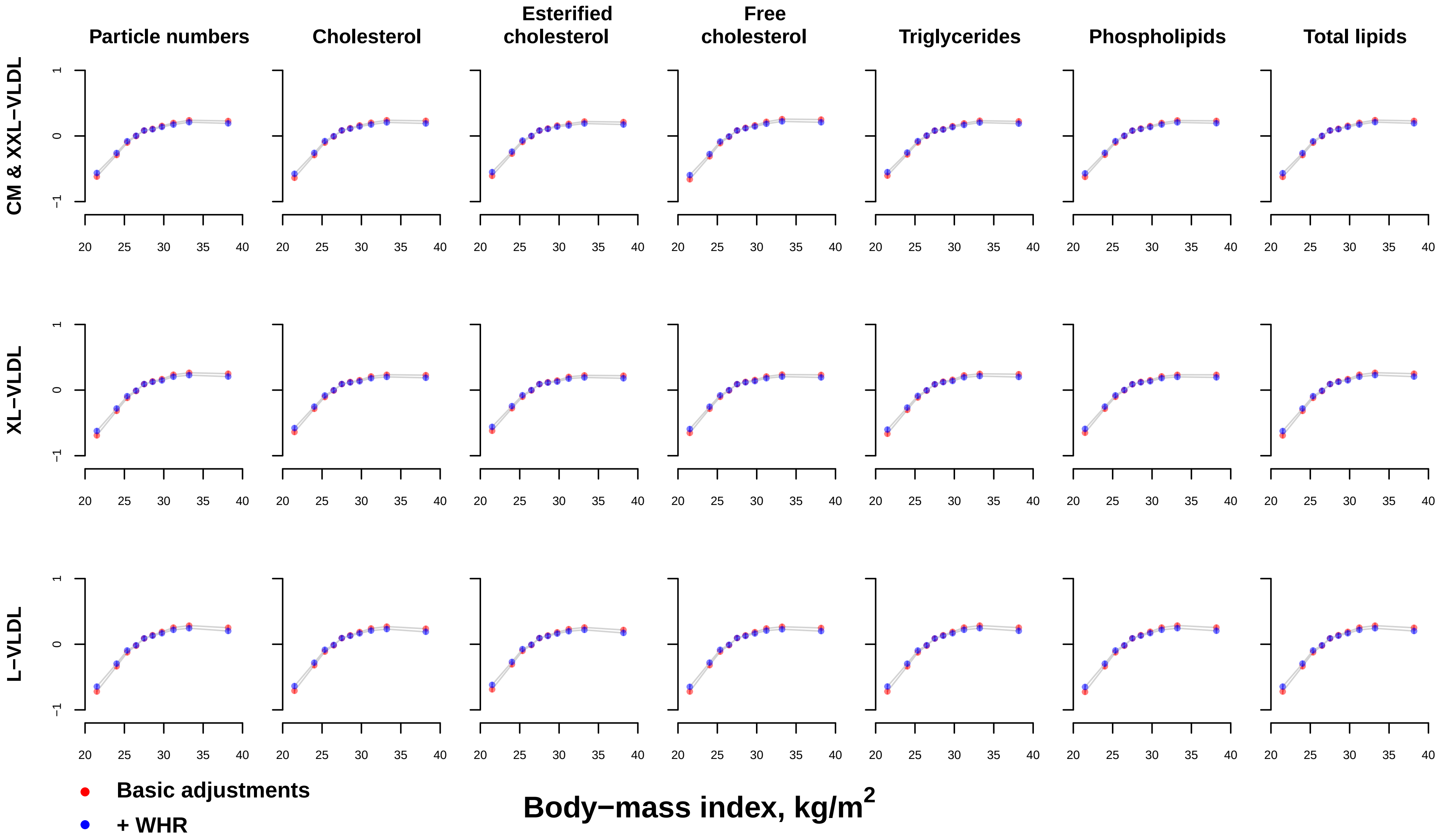

Points represent means (with 95% confidence intervals) of log−transformed (and then scaled to a mean of 0 and an SD of 1) NMR−biomarkers for each tenth of body−mass index. Exclusions as per Figure 2. Estimates are adjusted for age, district of residence, educational level, smoking, alcohol intake, sex−specific fifths of fasting duration, and NMR−experiment site. The range in the y−axis of each panel corresponds to +/− 1.0 standard deviations from the study population mean of the relevant log−transformed biomaker unless marked differently. The range in the x−axis of each panel corresponds to +/− 2.0 standard deviations from the study population mean BMI. NMR denotes nuclear magnetic resonance; VLDL, very low density lipoprotein; IDL, intermediate density lipoprotein; LDL, low density liporpotein; HDL, high density lipoprotein; FA, fatty acids; BMI, body−mass index; WC, waist cirfumference; WHR, waist−hip ratio; HC hip circumference.

Figure S1. NMR biomarkers by body−mass index levels before and after adjustment for WHR

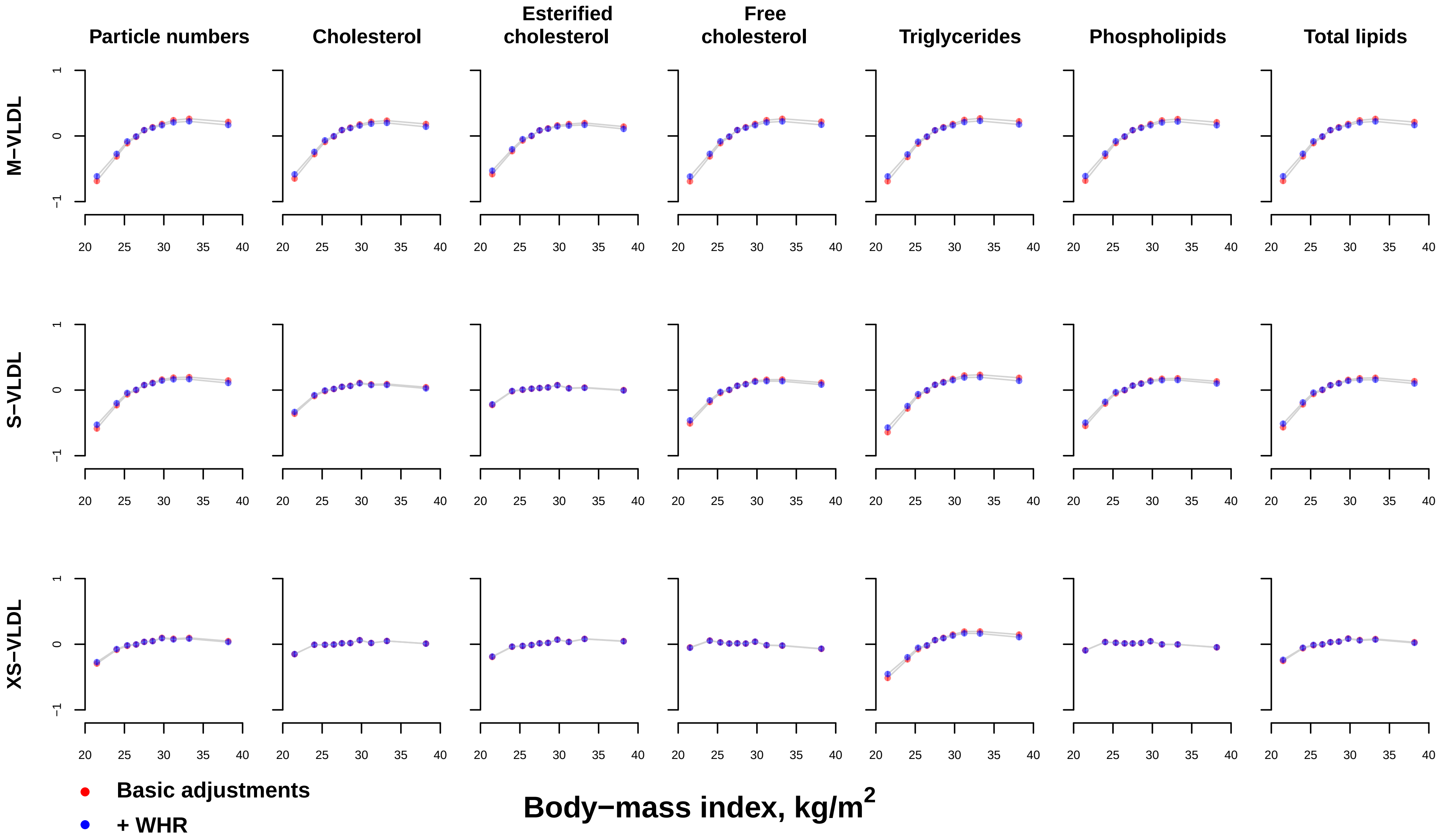

Points represent means (with 95% confidence intervals) of log−transformed (and then scaled to a mean of 0 and an SD of 1) NMR−biomarkers for each tenth of body−mass index. Exclusions as per Figure 2. Estimates are adjusted for age, district of residence, educational level, smoking, alcohol intake, sex−specific fifths of fasting duration, and NMR−experiment site. The range in the y−axis of each panel corresponds to +/− 1.0 standard deviations from the study population mean of the relevant log−transformed biomaker unless marked differently. The range in the x−axis of each panel corresponds to +/− 2.0 standard deviations from the study population mean BMI. NMR denotes nuclear magnetic resonance; VLDL, very low density lipoprotein; IDL, intermediate density lipoprotein; LDL, low density liporpotein; HDL, high density lipoprotein; FA, fatty acids; BMI, body−mass index; WC, waist cirfumference; WHR, waist−hip ratio; HC hip circumference.

Figure S1. NMR biomarkers by body−mass index levels before and after adjustment for WHR

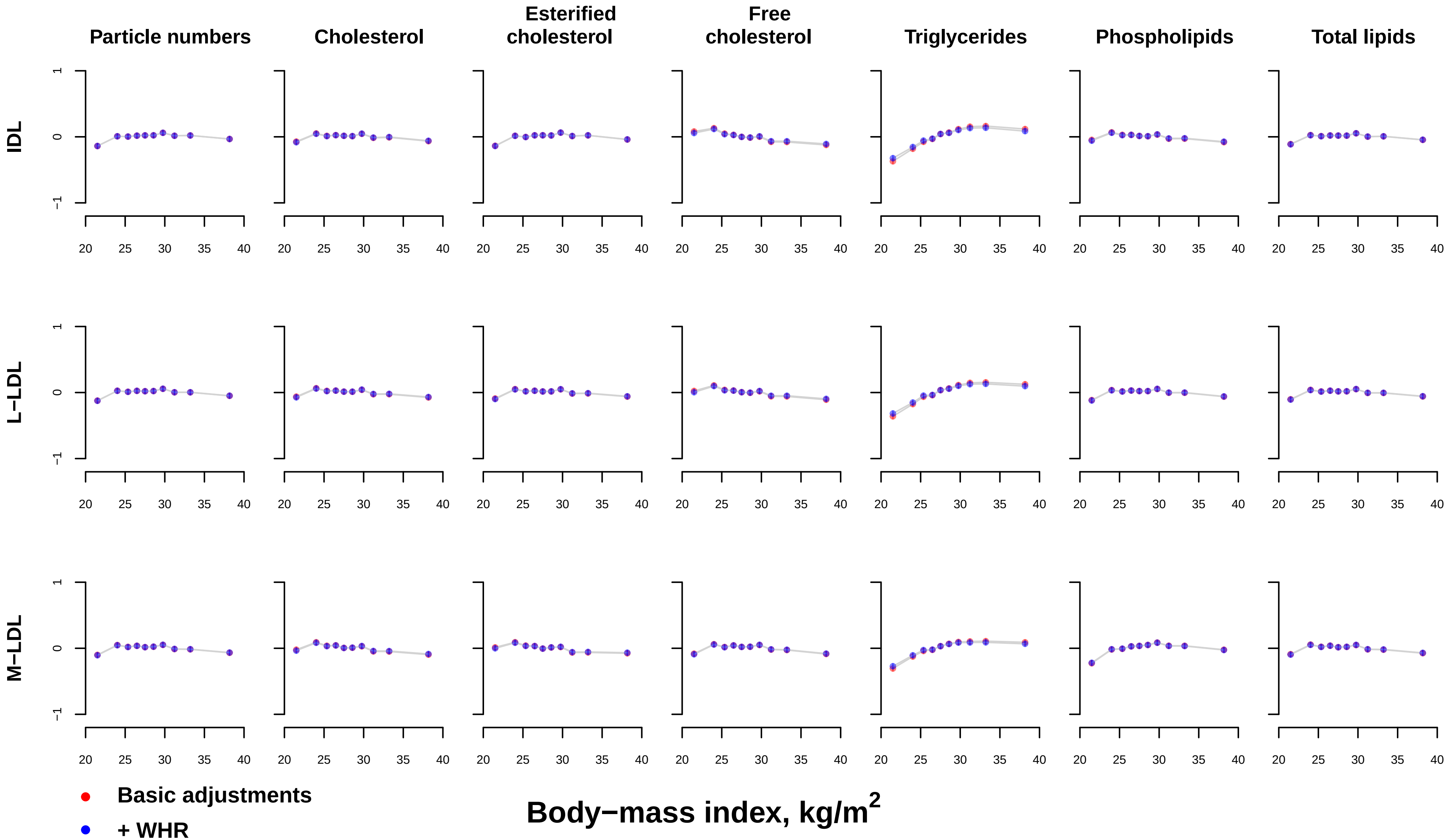

Points represent means (with 95% confidence intervals) of log−transformed (and then scaled to a mean of 0 and an SD of 1) NMR−biomarkers for each tenth of body−mass index. Exclusions as per Figure 2. Estimates are adjusted for age, district of residence, educational level, smoking, alcohol intake, sex−specific fifths of fasting duration, and NMR−experiment site. The range in the y−axis of each panel corresponds to +/− 1.0 standard deviations from the study population mean of the relevant log−transformed biomaker unless marked differently. The range in the x−axis of each panel corresponds to +/− 2.0 standard deviations from the study population mean BMI. NMR denotes nuclear magnetic resonance; VLDL, very low density lipoprotein; IDL, intermediate density lipoprotein; LDL, low density liporpotein; HDL, high density lipoprotein; FA, fatty acids; BMI, body−mass index; WC, waist cirfumference; WHR, waist−hip ratio; HC hip circumference.

Figure S1. NMR biomarkers by body−mass index levels before and after adjustment for WHR

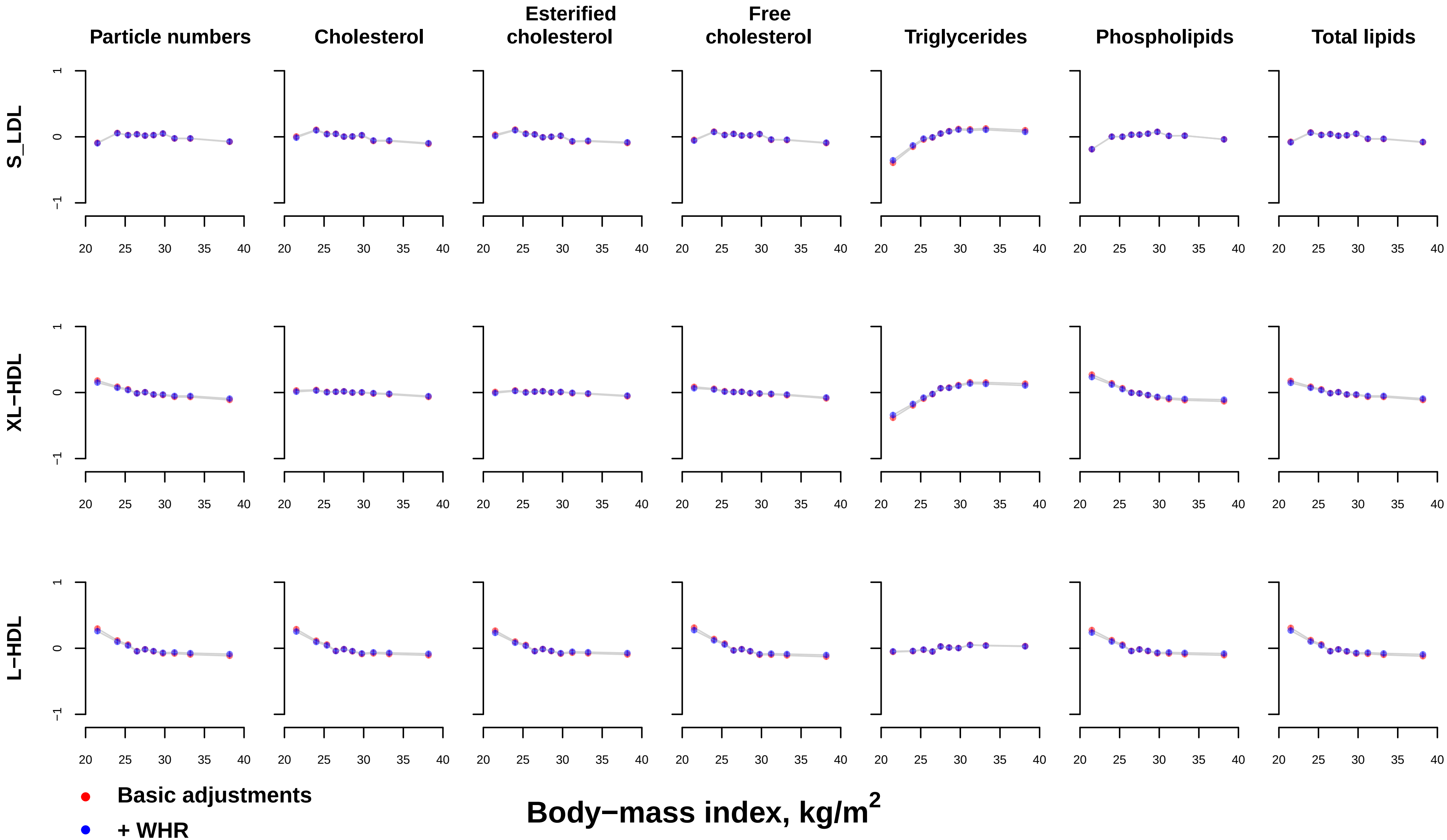

Points represent means (with 95% confidence intervals) of log−transformed (and then scaled to a mean of 0 and an SD of 1) NMR−biomarkers for each tenth of body−mass index. Exclusions as per Figure 2. Estimates are adjusted for age, district of residence, educational level, smoking, alcohol intake, sex−specific fifths of fasting duration, and NMR−experiment site. The range in the y−axis of each panel corresponds to +/− 1.0 standard deviations from the study population mean of the relevant log−transformed biomaker unless marked differently. The range in the x−axis of each panel corresponds to +/− 2.0 standard deviations from the study population mean BMI. NMR denotes nuclear magnetic resonance; VLDL, very low density lipoprotein; IDL, intermediate density lipoprotein; LDL, low density liporpotein; HDL, high density lipoprotein; FA, fatty acids; BMI, body−mass index; WC, waist cirfumference; WHR, waist−hip ratio; HC hip circumference.

Figure S1. NMR biomarkers by body−mass index levels before and after adjustment for WHR

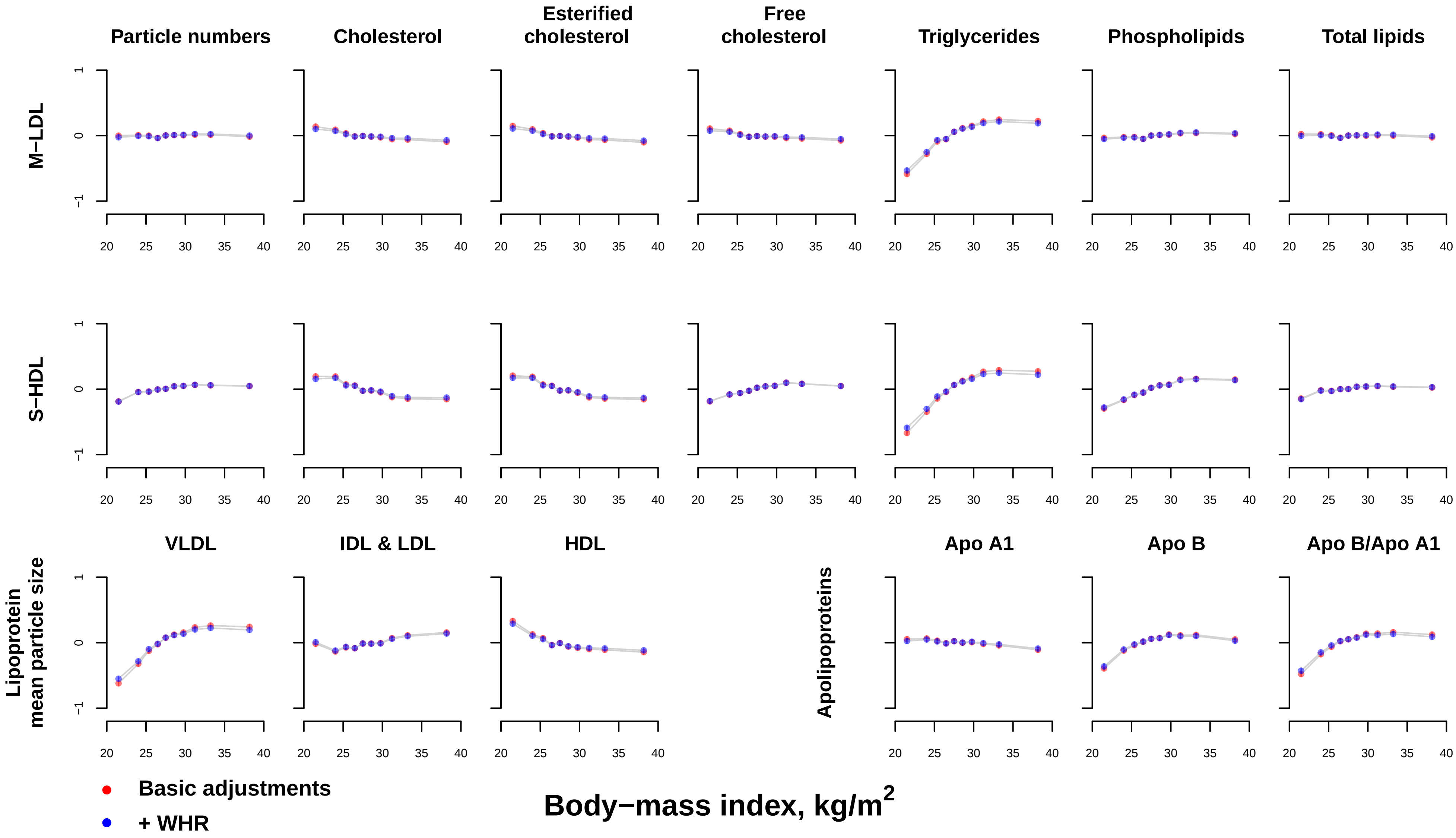

Points represent means (with 95% confidence intervals) of log−transformed (and then scaled to a mean of 0 and an SD of 1) NMR−biomarkers for each tenth of body−mass index. Exclusions as per Figure 2. Estimates are adjusted for age, district of residence, educational level, smoking, alcohol intake, sex−specific fifths of fasting duration, and NMR−experiment site. The range in the y−axis of each panel corresponds to +/− 1.0 standard deviations from the study population mean of the relevant log−transformed biomaker unless marked differently. The range in the x−axis of each panel corresponds to +/− 2.0 standard deviations from the study population mean BMI. NMR denotes nuclear magnetic resonance; VLDL, very low density lipoprotein; IDL, intermediate density lipoprotein; LDL, low density liporpotein; HDL, high density lipoprotein; FA, fatty acids; BMI, body−mass index; WC, waist cirfumference; WHR, waist−hip ratio; HC hip circumference.

Figure S1. NMR biomarkers by body−mass index levels before and after adjustment for WHR

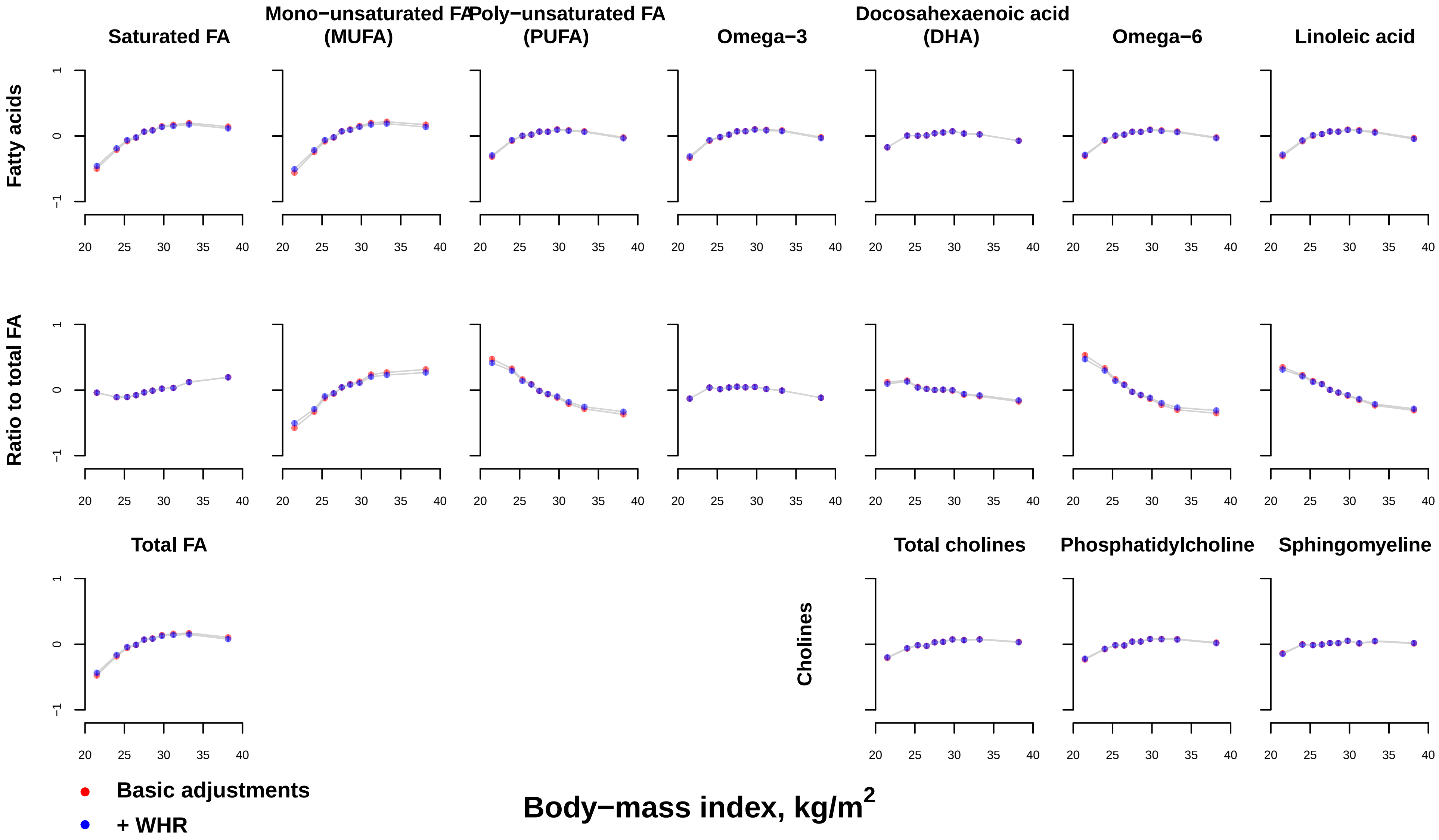

Points represent means (with 95% confidence intervals) of log−transformed (and then scaled to a mean of 0 and an SD of 1) NMR−biomarkers for each tenth of body−mass index. Exclusions as per Figure 2. Estimates are adjusted for age, district of residence, educational level, smoking, alcohol intake, sex−specific fifths of fasting duration, and NMR−experiment site. The range in the y−axis of each panel corresponds to +/− 1.0 standard deviations from the study population mean of the relevant log−transformed biomaker unless marked differently. The range in the x−axis of each panel corresponds to +/− 2.0 standard deviations from the study population mean BMI. NMR denotes nuclear magnetic resonance; VLDL, very low density lipoprotein; IDL, intermediate density lipoprotein; LDL, low density liporpotein; HDL, high density lipoprotein; FA, fatty acids; BMI, body−mass index; WC, waist cirfumference; WHR, waist−hip ratio; HC hip circumference.

Figure S1. NMR biomarkers by body−mass index levels before and after adjustment for WHR

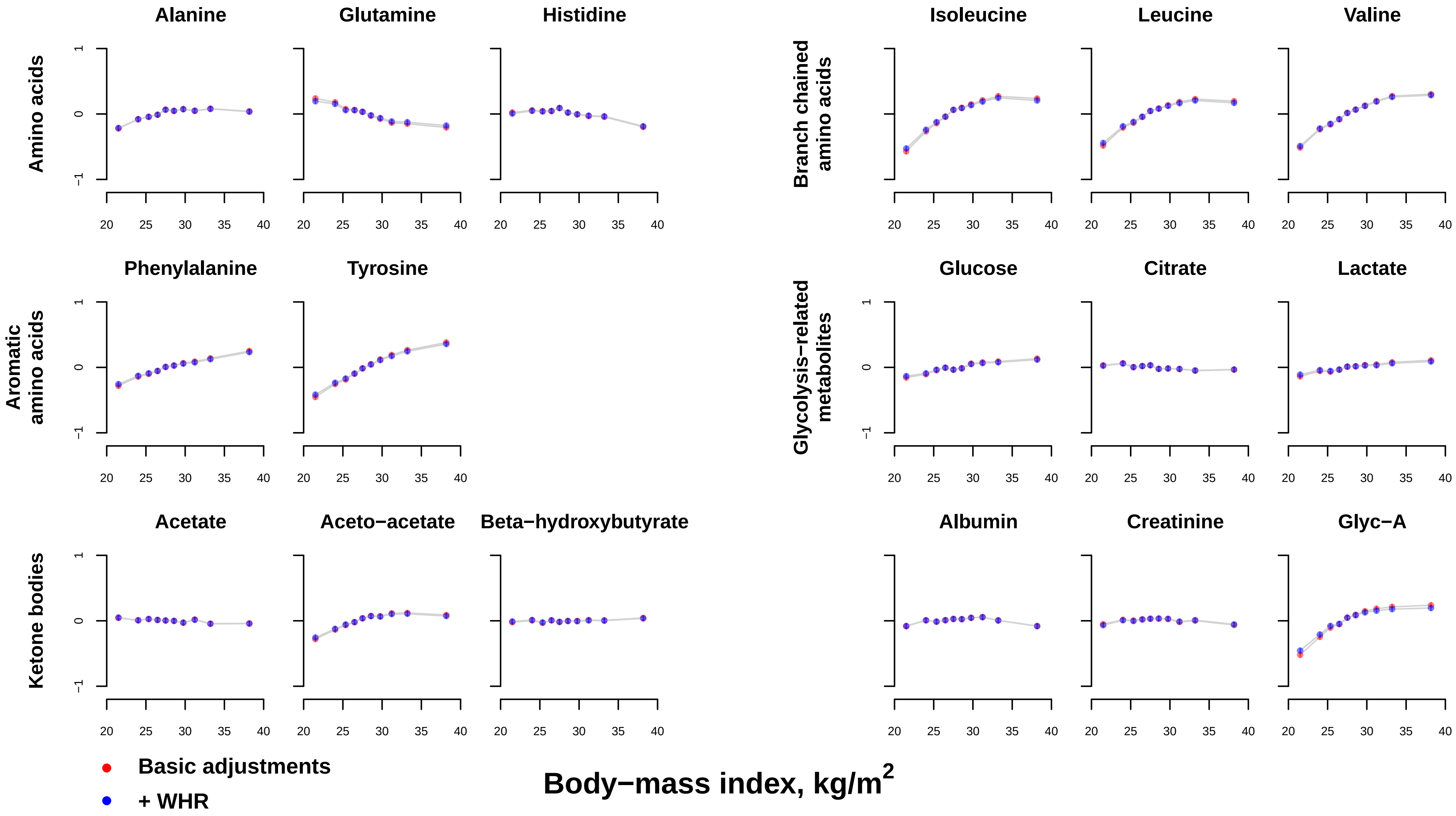

Points represent means (with 95% confidence intervals) of log−transformed (and then scaled to a mean of 0 and an SD of 1) NMR−biomarkers for each tenth of body−mass index. Exclusions as per Figure 2. Estimates are adjusted for age, district of residence, educational level, smoking, alcohol intake, sex−specific fifths of fasting duration, and NMR−experiment site. The range in the y−axis of each panel corresponds to +/− 1.0 standard deviations from the study population mean of the relevant log−transformed biomaker unless marked differently. The range in the x−axis of each panel corresponds to +/− 2.0 standard deviations from the study population mean BMI. NMR denotes nuclear magnetic resonance; VLDL, very low density lipoprotein; IDL, intermediate density lipoprotein; LDL, low density liporpotein; HDL, high density lipoprotein; FA, fatty acids; BMI, body−mass index; WC, waist cirfumference; WHR, waist−hip ratio; HC hip circumference.

Figure S2. NMR biomarkers by waist−hip ratio levels before and after adjustment for BMI

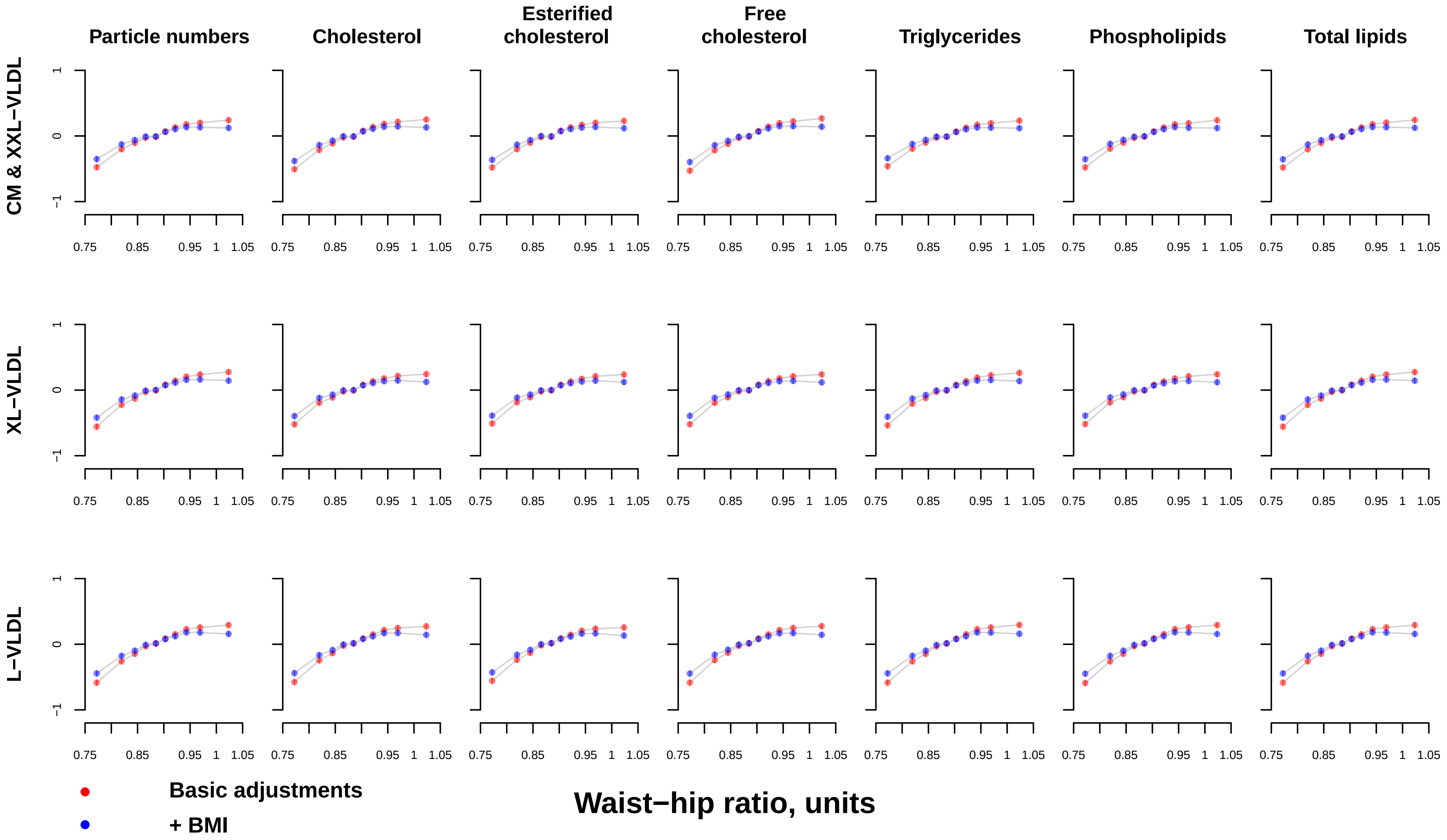

Points represent means (with 95% confidence intervals) of log−transformed (and then scaled to a mean of 0 and an SD of 1) NMR−biomarkers for each tenth of waist−hip ratio. Exclusions as per Figure 2. Estimates are adjusted for age, district of residence, educational level, smoking, alcohol intake, sex−specific fifths of fasting duration, and NMR−experiment site. The range in the y−axis of each panel corresponds to +/− 1.0 standard deviations from the study population mean of the relevant log−transformed biomaker unless marked differently. The range in the x−axis of each panel corresponds to +/− 2.0 standard deviations from the study population mean BMI. NMR denotes nuclear magnetic resonance; VLDL, very low density lipoprotein; IDL, intermediate density lipoprotein; LDL, low density liporpotein; HDL, high density lipoprotein; FA, fatty acids; BMI, body−mass index; WC, waist cirfumference; WHR, waist−hip ratio; HC hip circumference.

Figure S2. NMR biomarkers by waist−hip ratio levels before and after adjustment for BMI

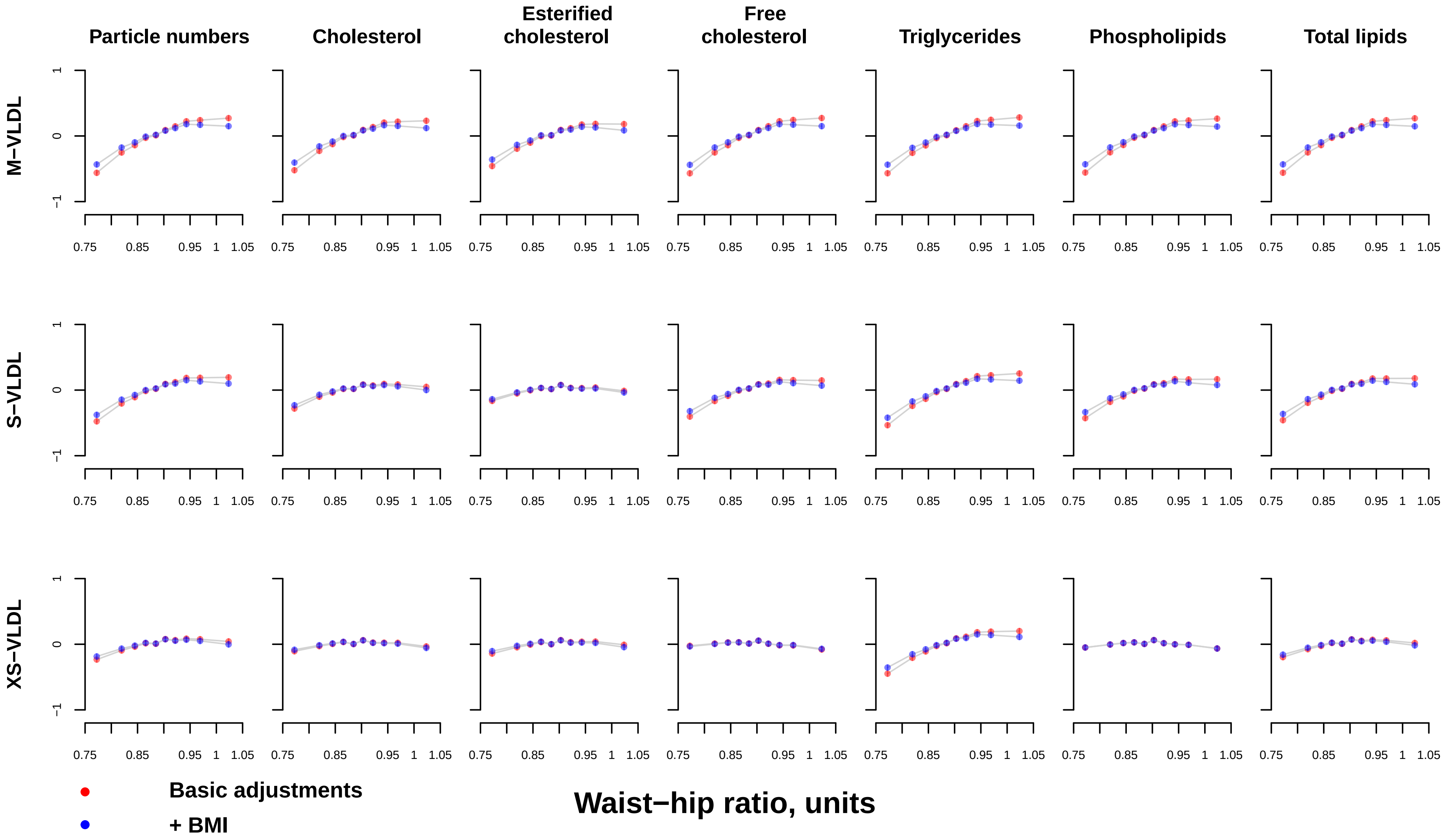

Points represent means (with 95% confidence intervals) of log−transformed (and then scaled to a mean of 0 and an SD of 1) NMR−biomarkers for each tenth of waist−hip ratio. Exclusions as per Figure 2. Estimates are adjusted for age, district of residence, educational level, smoking, alcohol intake, sex−specific fifths of fasting duration, and NMR−experiment site. The range in the y−axis of each panel corresponds to +/− 1.0 standard deviations from the study population mean of the relevant log−transformed biomaker unless marked differently. The range in the x−axis of each panel corresponds to +/− 2.0 standard deviations from the study population mean BMI. NMR denotes nuclear magnetic resonance; VLDL, very low density lipoprotein; IDL, intermediate density lipoprotein; LDL, low density liporpotein; HDL, high density lipoprotein; FA, fatty acids; BMI, body−mass index; WC, waist cirfumference; WHR, waist−hip ratio; HC hip circumference.

Figure S2. NMR biomarkers by waist−hip ratio levels before and after adjustment for BMI

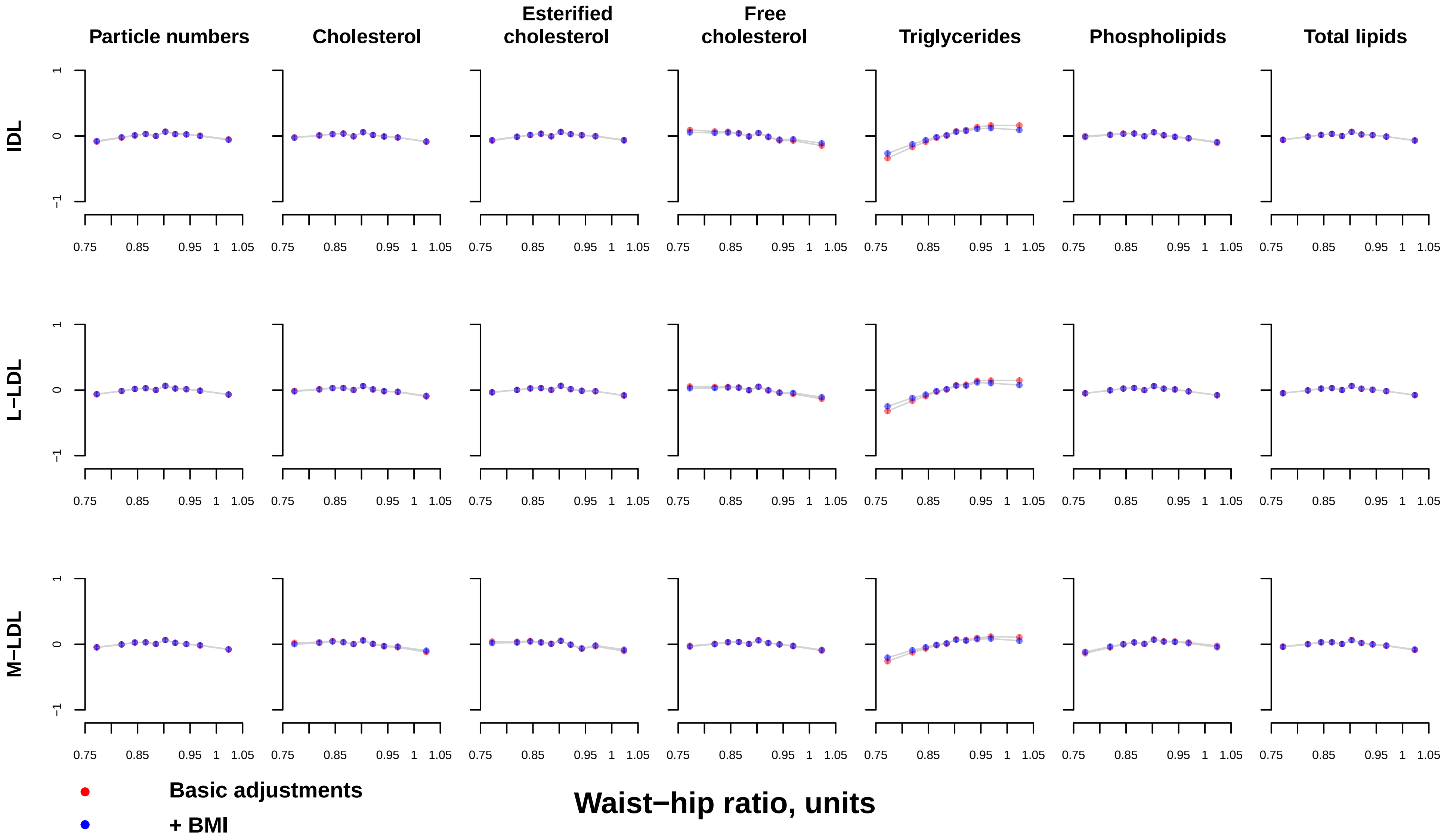

Points represent means (with 95% confidence intervals) of log−transformed (and then scaled to a mean of 0 and an SD of 1) NMR−biomarkers for each tenth of waist−hip ratio. Exclusions as per Figure 2. Estimates are adjusted for age, district of residence, educational level, smoking, alcohol intake, sex−specific fifths of fasting duration, and NMR−experiment site. The range in the y−axis of each panel corresponds to +/− 1.0 standard deviations from the study population mean of the relevant log−transformed biomaker unless marked differently. The range in the x−axis of each panel corresponds to +/− 2.0 standard deviations from the study population mean BMI. NMR denotes nuclear magnetic resonance; VLDL, very low density lipoprotein; IDL, intermediate density lipoprotein; LDL, low density liporpotein; HDL, high density lipoprotein; FA, fatty acids; BMI, body−mass index; WC, waist cirfumference; WHR, waist−hip ratio; HC hip circumference.

Figure S2. NMR biomarkers by waist−hip ratio levels before and after adjustment for BMI

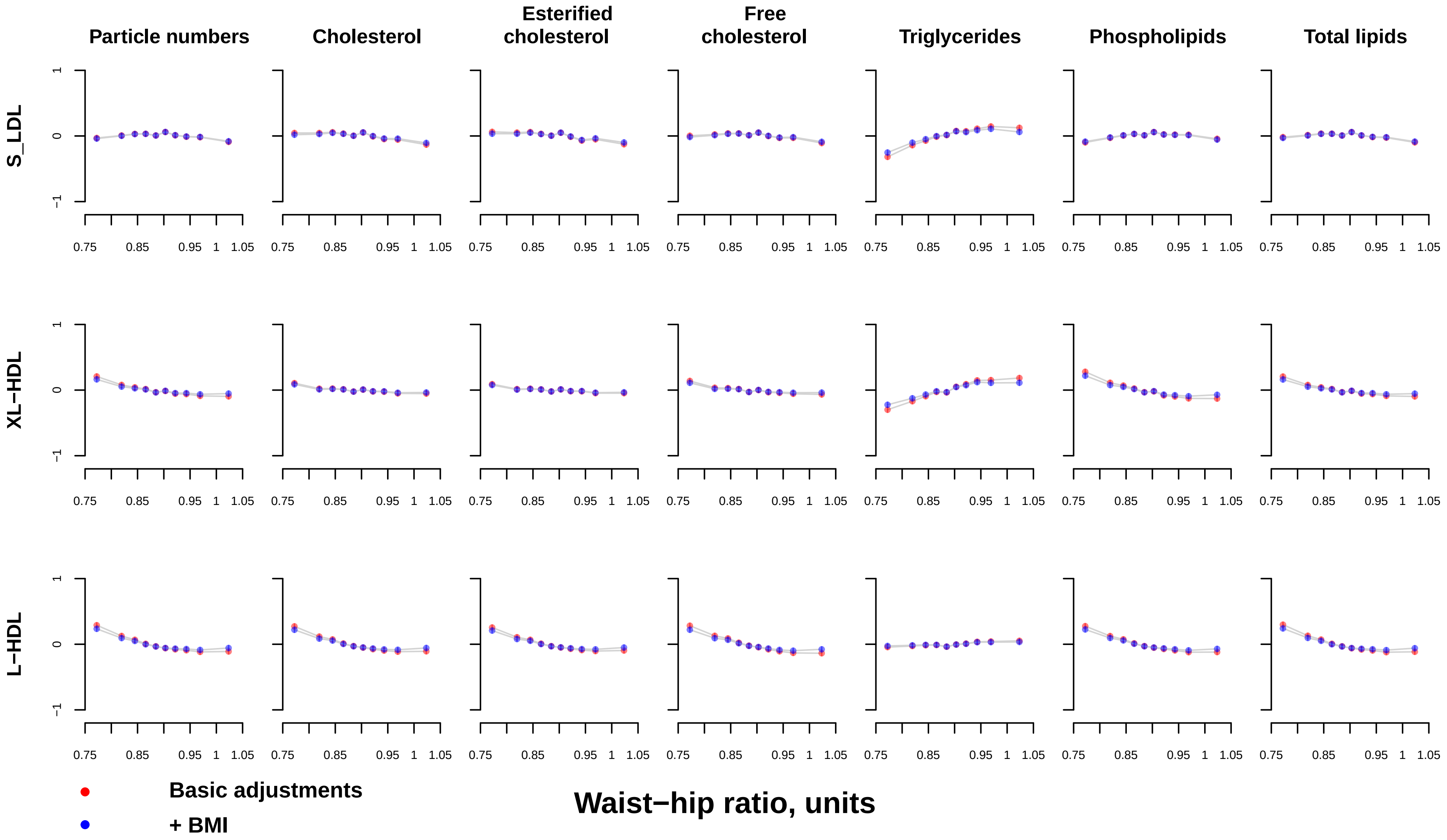

Points represent means (with 95% confidence intervals) of log−transformed (and then scaled to a mean of 0 and an SD of 1) NMR−biomarkers for each tenth of waist−hip ratio. Exclusions as per Figure 2. Estimates are adjusted for age, district of residence, educational level, smoking, alcohol intake, sex−specific fifths of fasting duration, and NMR−experiment site. The range in the y−axis of each panel corresponds to +/− 1.0 standard deviations from the study population mean of the relevant log−transformed biomaker unless marked differently. The range in the x−axis of each panel corresponds to +/− 2.0 standard deviations from the study population mean BMI. NMR denotes nuclear magnetic resonance; VLDL, very low density lipoprotein; IDL, intermediate density lipoprotein; LDL, low density liporpotein; HDL, high density lipoprotein; FA, fatty acids; BMI, body−mass index; WC, waist cirfumference; WHR, waist−hip ratio; HC hip circumference.

Figure S2. NMR biomarkers by waist−hip ratio levels before and after adjustment for BMI

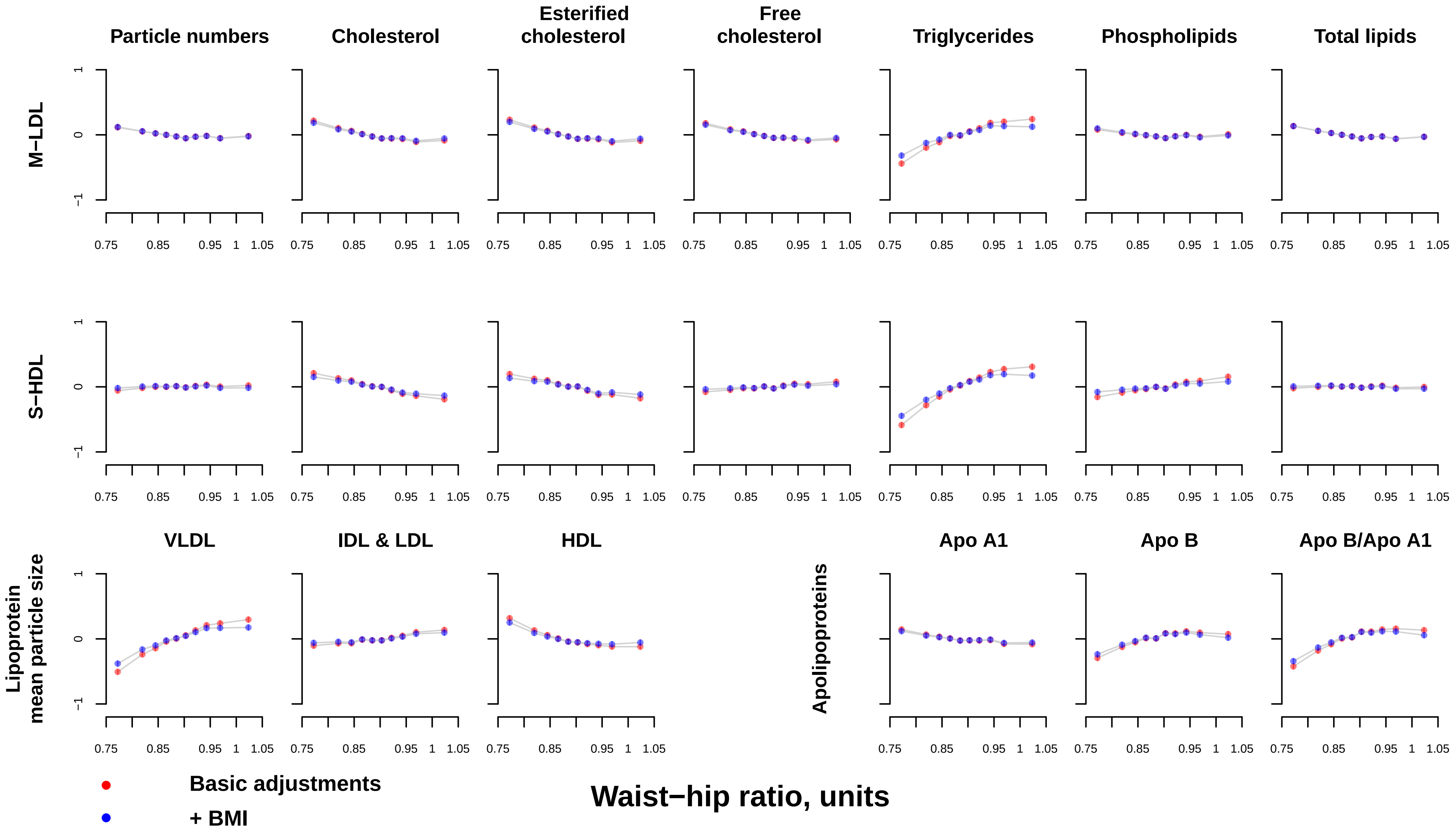

Points represent means (with 95% confidence intervals) of log−transformed (and then scaled to a mean of 0 and an SD of 1) NMR−biomarkers for each tenth of waist−hip ratio. Exclusions as per Figure 2. Estimates are adjusted for age, district of residence, educational level, smoking, alcohol intake, sex−specific fifths of fasting duration, and NMR−experiment site. The range in the y−axis of each panel corresponds to +/− 1.0 standard deviations from the study population mean of the relevant log−transformed biomaker unless marked differently. The range in the x−axis of each panel corresponds to +/− 2.0 standard deviations from the study population mean BMI. NMR denotes nuclear magnetic resonance; VLDL, very low density lipoprotein; IDL, intermediate density lipoprotein; LDL, low density liporpotein; HDL, high density lipoprotein; FA, fatty acids; BMI, body−mass index; WC, waist cirfumference; WHR, waist−hip ratio; HC hip circumference.

Figure S2. NMR biomarkers by waist−hip ratio levels before and after adjustment for BMI

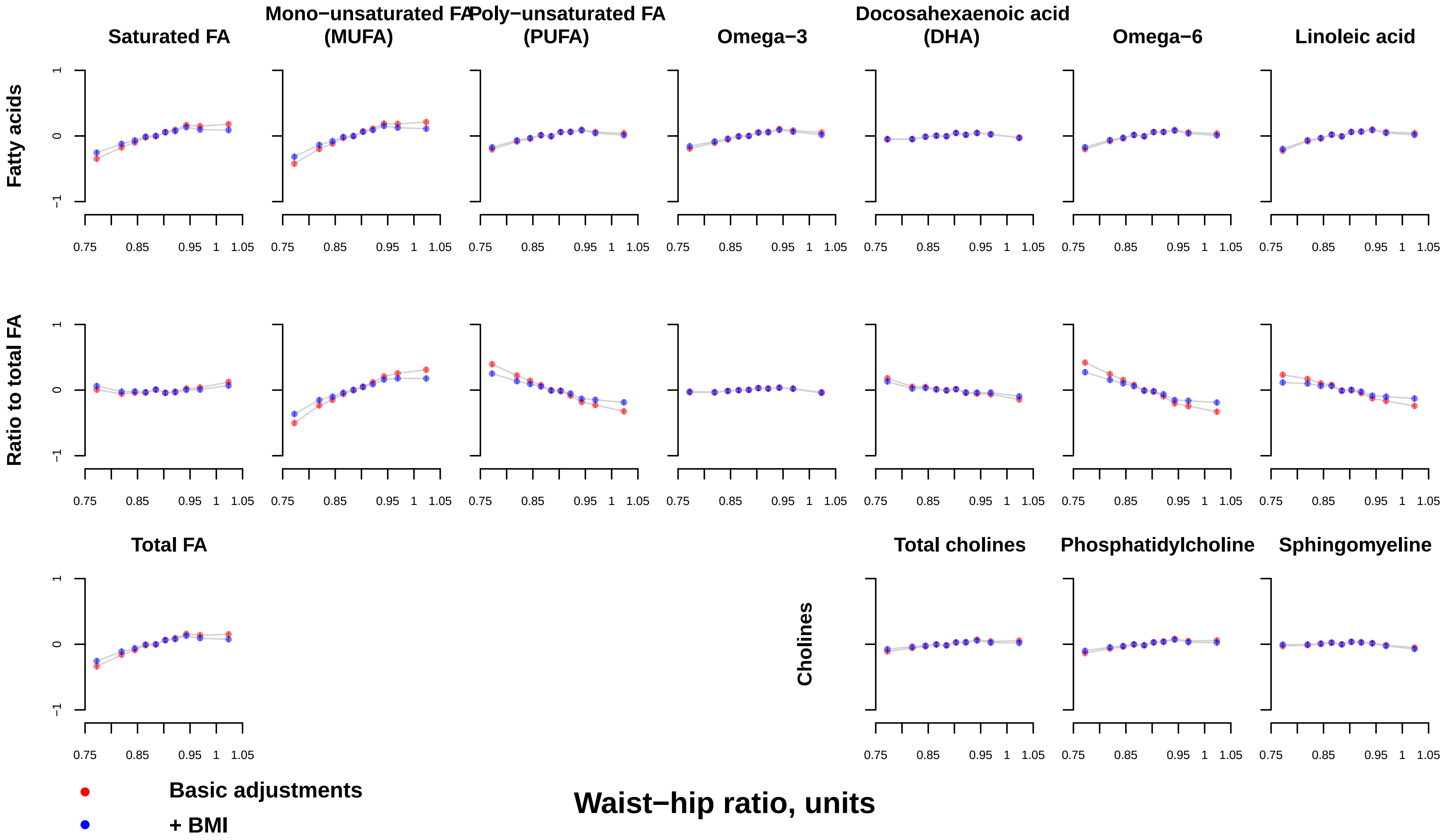

Points represent means (with 95% confidence intervals) of log−transformed (and then scaled to a mean of 0 and an SD of 1) NMR−biomarkers for each tenth of waist−hip ratio. Exclusions as per Figure 2. Estimates are adjusted for age, district of residence, educational level, smoking, alcohol intake, sex−specific fifths of fasting duration, and NMR−experiment site. The range in the y−axis of each panel corresponds to +/− 1.0 standard deviations from the study population mean of the relevant log−transformed biomaker unless marked differently. The range in the x−axis of each panel corresponds to +/− 2.0 standard deviations from the study population mean BMI. NMR denotes nuclear magnetic resonance; VLDL, very low density lipoprotein; IDL, intermediate density lipoprotein; LDL, low density liporpotein; HDL, high density lipoprotein; FA, fatty acids; BMI, body−mass index; WC, waist cirfumference; WHR, waist−hip ratio; HC hip circumference.

Figure S2. NMR biomarkers by waist−hip ratio levels before and after adjustment for BMI

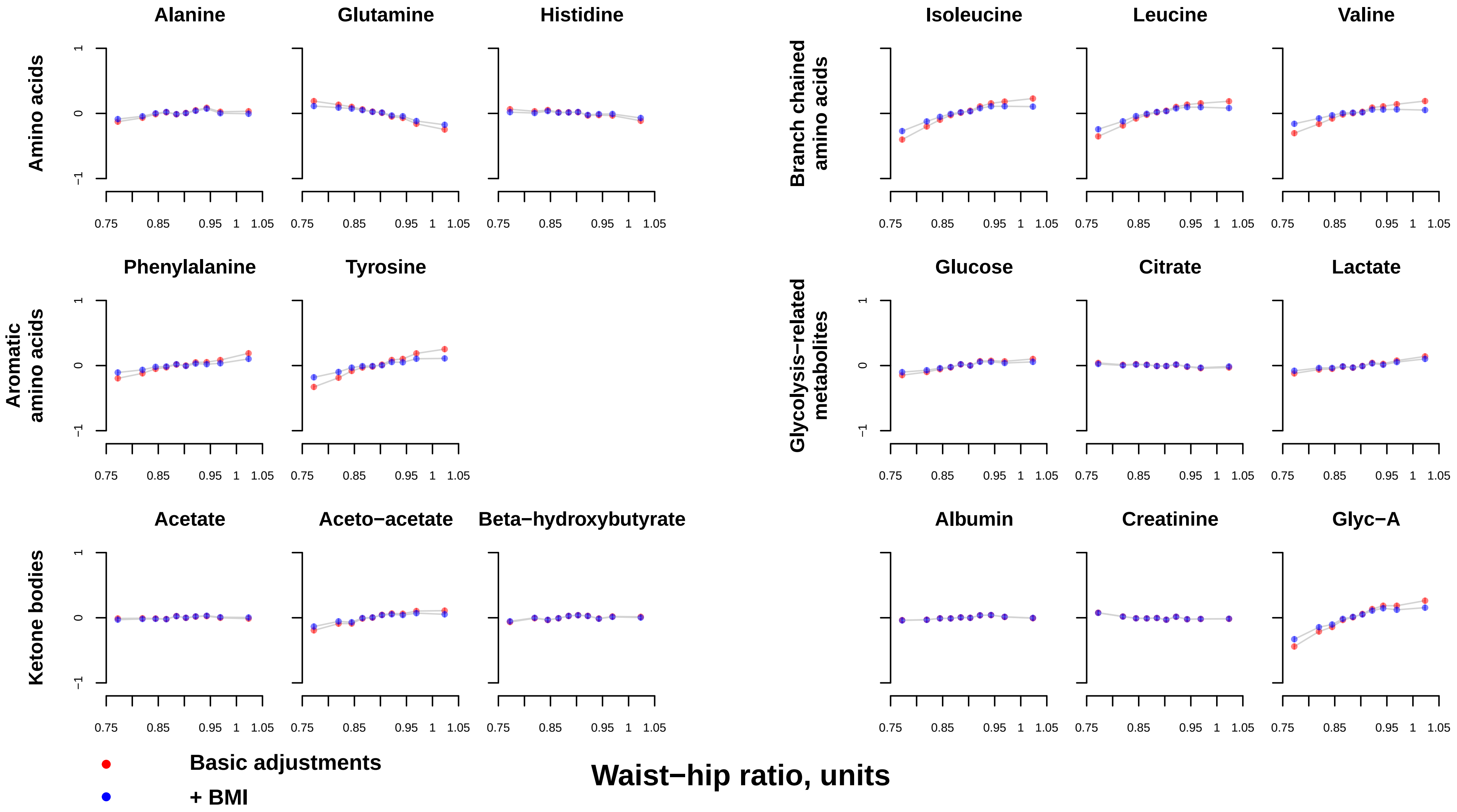

Points represent means (with 95% confidence intervals) of log−transformed (and then scaled to a mean of 0 and an SD of 1) NMR−biomarkers for each tenth of waist−hip ratio. Exclusions as per Figure 2. Estimates are adjusted for age, district of residence, educational level, smoking, alcohol intake, sex−specific fifths of fasting duration, and NMR−experiment site. The range in the y−axis of each panel corresponds to +/− 1.0 standard deviations from the study population mean of the relevant log−transformed biomaker unless marked differently. The range in the x−axis of each panel corresponds to +/− 2.0 standard deviations from the study population mean BMI. NMR denotes nuclear magnetic resonance; VLDL, very low density lipoprotein; IDL, intermediate density lipoprotein; LDL, low density liporpotein; HDL, high density lipoprotein; FA, fatty acids; BMI, body−mass index; WC, waist cirfumference; WHR, waist−hip ratio; HC hip circumference.

Figure S3. NMR biomarkers by waist circumference levels before and after adjustment for HC and BMI

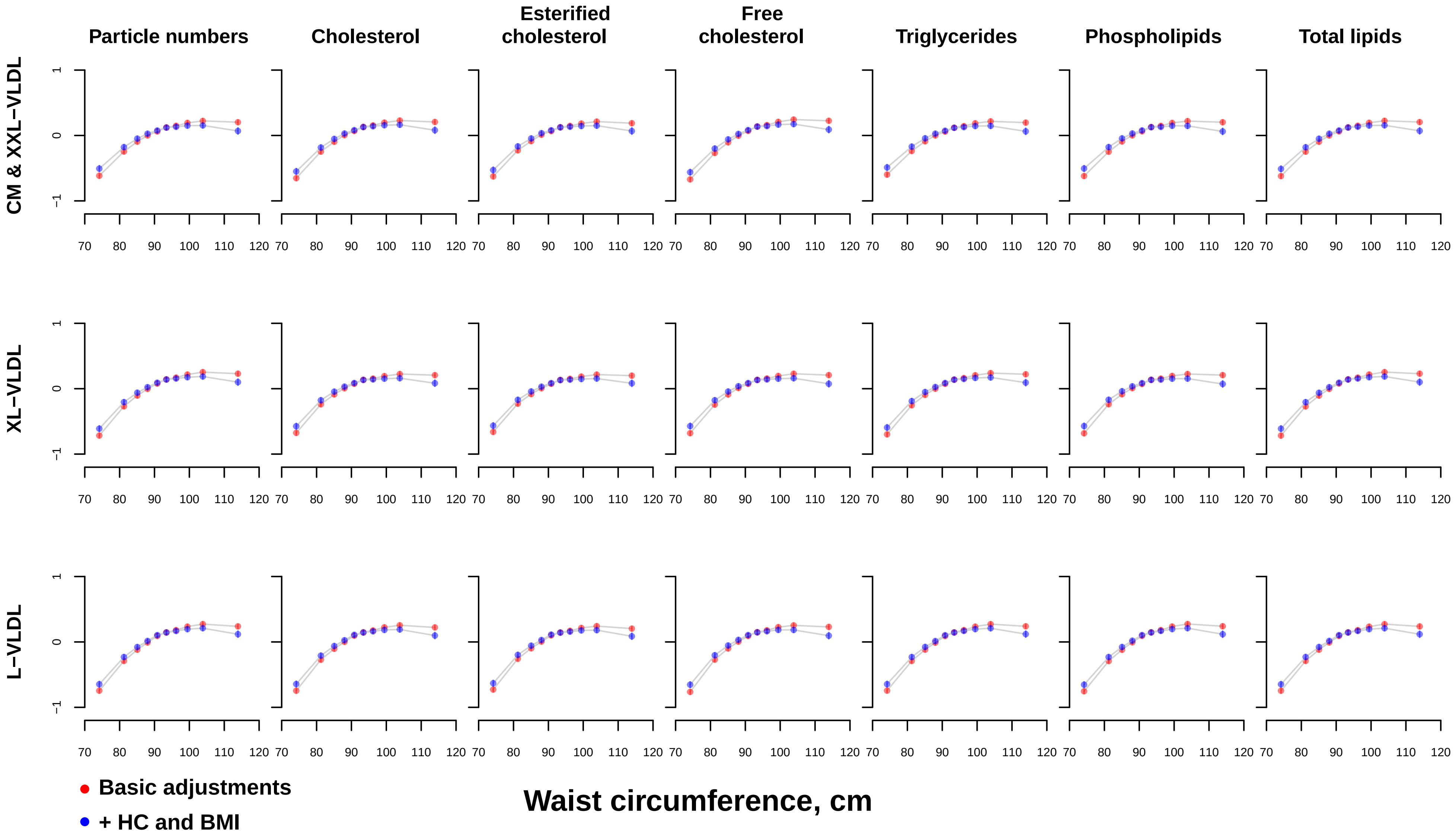

Points represent means (with 95% confidence intervals) of log−transformed (and then scaled to a mean of 0 and an SD of 1) NMR−biomarkers for each tenth of waist circumference. Exclusions as per Figure 2. Estimates are adjusted for age, district of residence, educational level, smoking, alcohol intake, sex−specific fifths of fasting duration, and NMR−experiment site. The range in the y−axis of each panel corresponds to +/− 1.0 standard deviations from the study population mean of the relevant log−transformed biomaker unless marked differently. The range in the x−axis of each panel corresponds to +/− 2.0 standard deviations from the study population mean BMI. NMR denotes nuclear magnetic resonance; VLDL, very low density lipoprotein; IDL, intermediate density lipoprotein; LDL, low density liporpotein; HDL, high density lipoprotein; FA, fatty acids; BMI, body−mass index; WC, waist cirfumference; WHR, waist−hip ratio; HC hip circumference.

Figure S3. NMR biomarkers by waist circumference levels before and after adjustment for HC and BMI

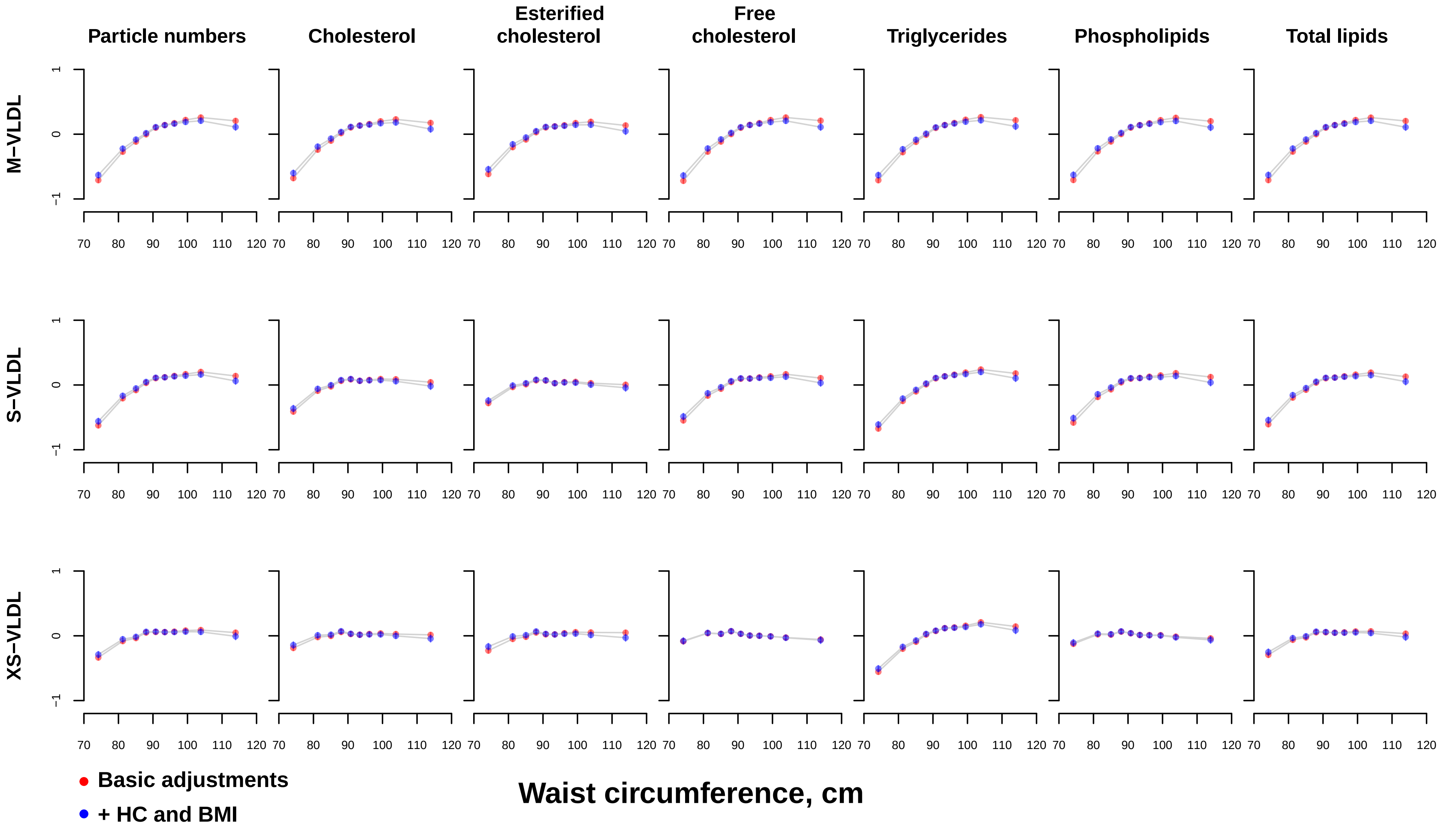

Points represent means (with 95% confidence intervals) of log−transformed (and then scaled to a mean of 0 and an SD of 1) NMR−biomarkers for each tenth of waist circumference. Exclusions as per Figure 2. Estimates are adjusted for age, district of residence, educational level, smoking, alcohol intake, sex−specific fifths of fasting duration, and NMR−experiment site. The range in the y−axis of each panel corresponds to +/− 1.0 standard deviations from the study population mean of the relevant log−transformed biomaker unless marked differently. The range in the x−axis of each panel corresponds to +/− 2.0 standard deviations from the study population mean BMI. NMR denotes nuclear magnetic resonance; VLDL, very low density lipoprotein; IDL, intermediate density lipoprotein; LDL, low density liporpotein; HDL, high density lipoprotein; FA, fatty acids; BMI, body−mass index; WC, waist cirfumference; WHR, waist−hip ratio; HC hip circumference.

Figure S3. NMR biomarkers by waist circumference levels before and after adjustment for HC and BMI

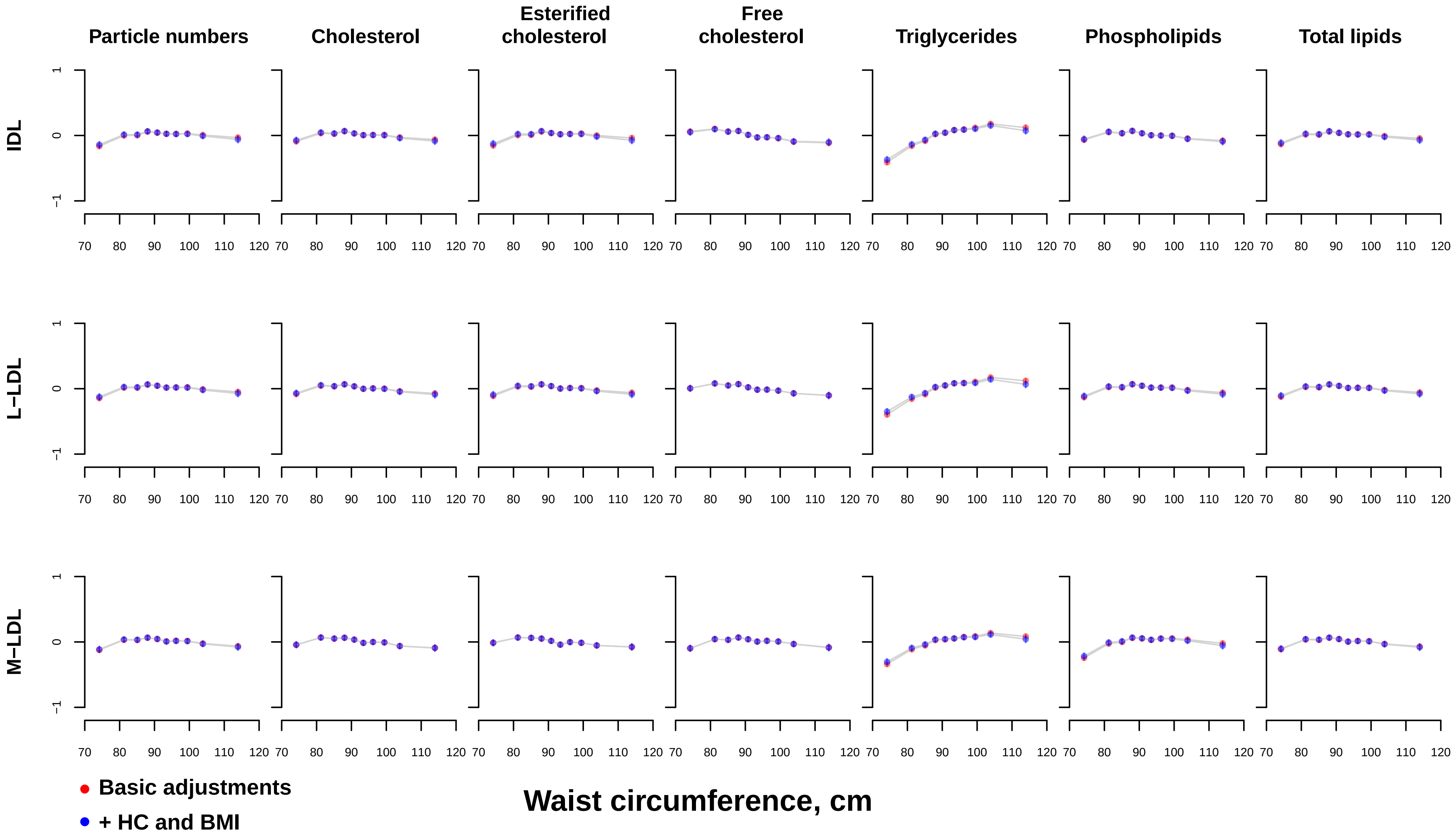

Points represent means (with 95% confidence intervals) of log−transformed (and then scaled to a mean of 0 and an SD of 1) NMR−biomarkers for each tenth of waist circumference. Exclusions as per Figure 2. Estimates are adjusted for age, district of residence, educational level, smoking, alcohol intake, sex−specific fifths of fasting duration, and NMR−experiment site. The range in the y−axis of each panel corresponds to +/− 1.0 standard deviations from the study population mean of the relevant log−transformed biomaker unless marked differently. The range in the x−axis of each panel corresponds to +/− 2.0 standard deviations from the study population mean BMI. NMR denotes nuclear magnetic resonance; VLDL, very low density lipoprotein; IDL, intermediate density lipoprotein; LDL, low density liporpotein; HDL, high density lipoprotein; FA, fatty acids; BMI, body−mass index; WC, waist cirfumference; WHR, waist−hip ratio; HC hip circumference.

Figure S3. NMR biomarkers by waist circumference levels before and after adjustment for HC and BMI

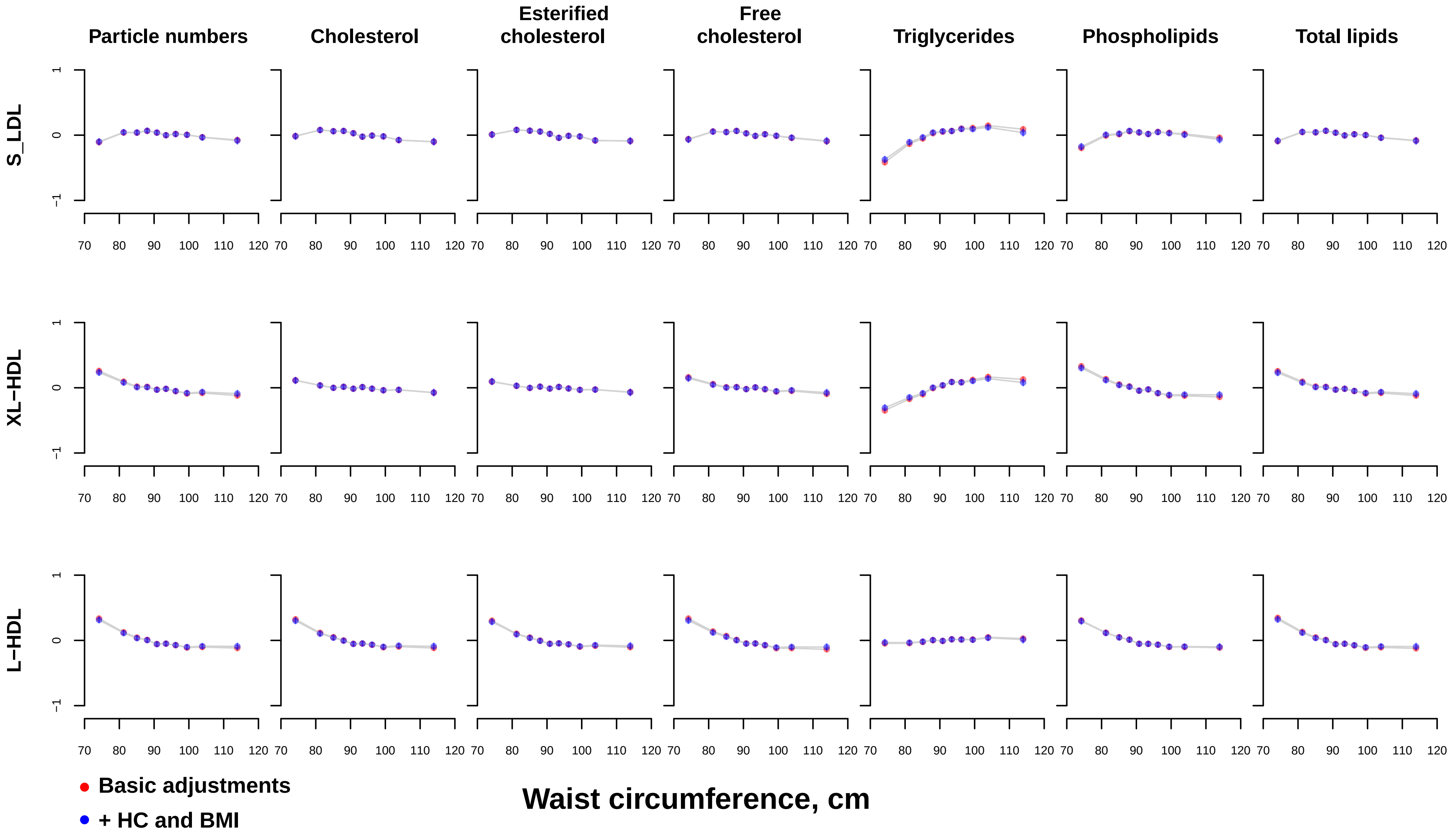

Points represent means (with 95% confidence intervals) of log−transformed (and then scaled to a mean of 0 and an SD of 1) NMR−biomarkers for each tenth of waist circumference. Exclusions as per Figure 2. Estimates are adjusted for age, district of residence, educational level, smoking, alcohol intake, sex−specific fifths of fasting duration, and NMR−experiment site. The range in the y−axis of each panel corresponds to +/− 1.0 standard deviations from the study population mean of the relevant log−transformed biomaker unless marked differently. The range in the x−axis of each panel corresponds to +/− 2.0 standard deviations from the study population mean BMI. NMR denotes nuclear magnetic resonance; VLDL, very low density lipoprotein; IDL, intermediate density lipoprotein; LDL, low density liporpotein; HDL, high density lipoprotein; FA, fatty acids; BMI, body−mass index; WC, waist cirfumference; WHR, waist−hip ratio; HC hip circumference.

Figure S3. NMR biomarkers by waist circumference levels before and after adjustment for HC and BMI

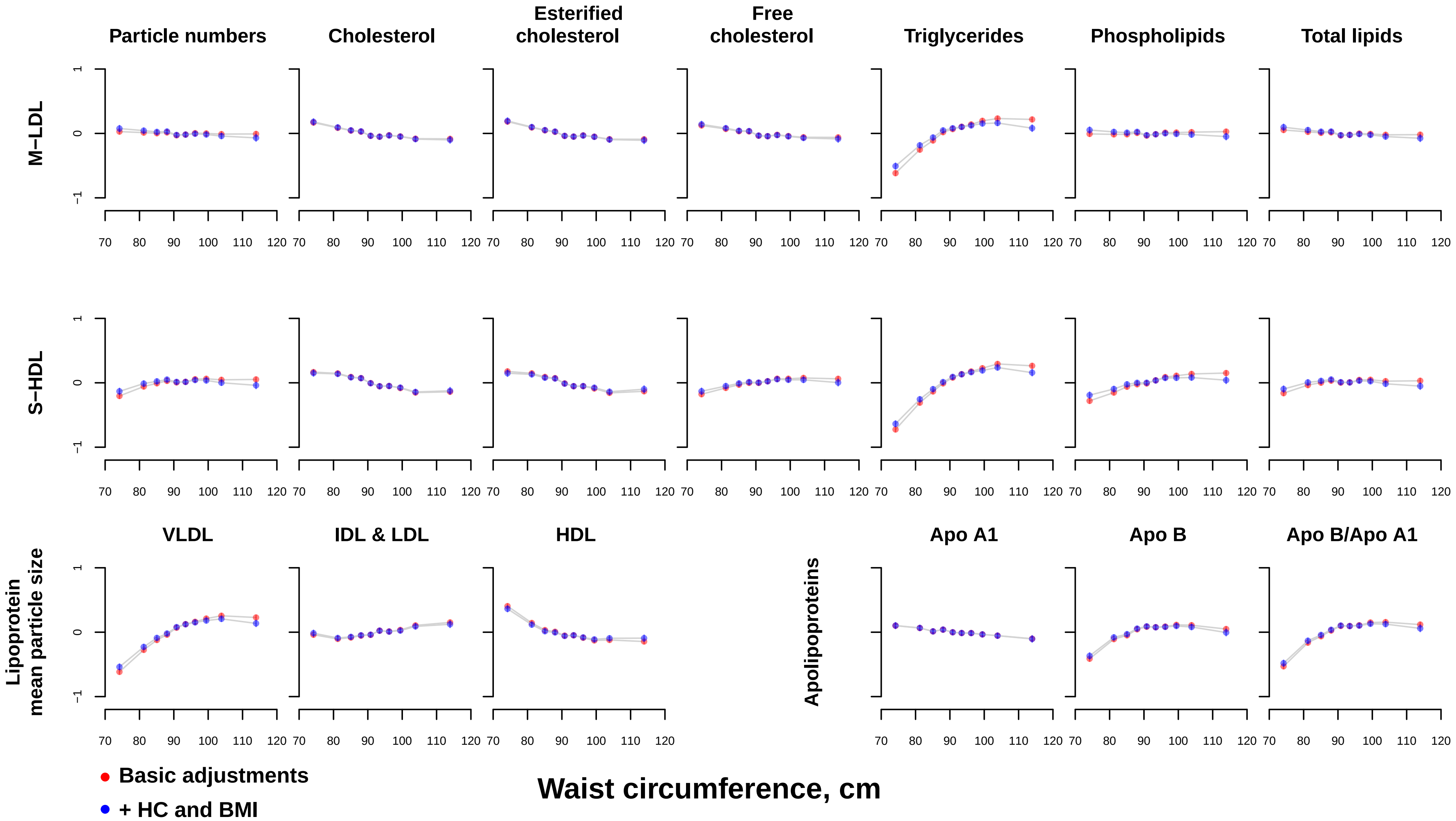

Points represent means (with 95% confidence intervals) of log−transformed (and then scaled to a mean of 0 and an SD of 1) NMR−biomarkers for each tenth of waist circumference. Exclusions as per Figure 2. Estimates are adjusted for age, district of residence, educational level, smoking, alcohol intake, sex−specific fifths of fasting duration, and NMR−experiment site. The range in the y−axis of each panel corresponds to +/− 1.0 standard deviations from the study population mean of the relevant log−transformed biomaker unless marked differently. The range in the x−axis of each panel corresponds to +/− 2.0 standard deviations from the study population mean BMI. NMR denotes nuclear magnetic resonance; VLDL, very low density lipoprotein; IDL, intermediate density lipoprotein; LDL, low density liporpotein; HDL, high density lipoprotein; FA, fatty acids; BMI, body−mass index; WC, waist cirfumference; WHR, waist−hip ratio; HC hip circumference.

Figure S3. NMR biomarkers by waist circumference levels before and after adjustment for HC and BMI

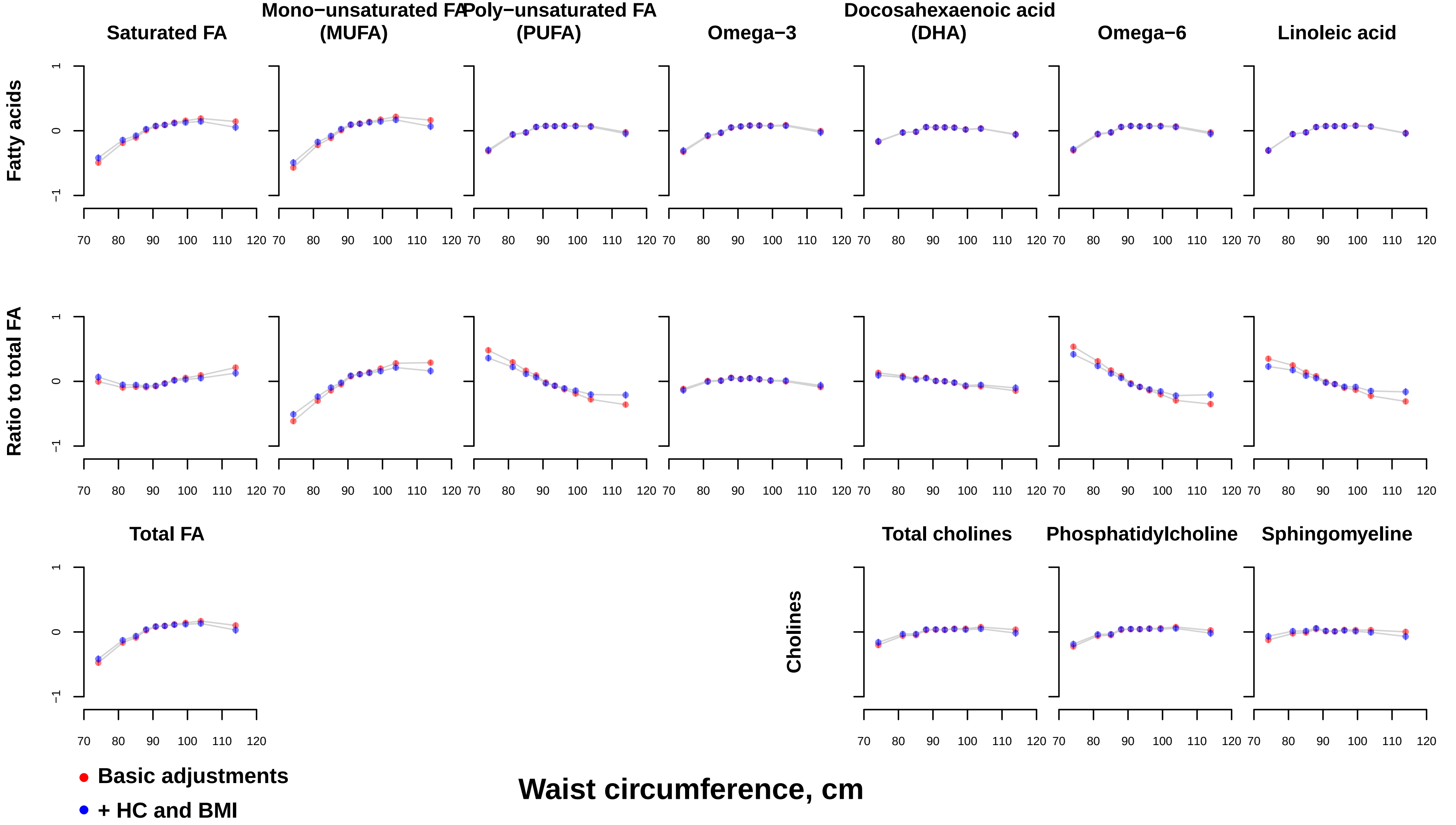

Points represent means (with 95% confidence intervals) of log−transformed (and then scaled to a mean of 0 and an SD of 1) NMR−biomarkers for each tenth of waist circumference. Exclusions as per Figure 2. Estimates are adjusted for age, district of residence, educational level, smoking, alcohol intake, sex−specific fifths of fasting duration, and NMR−experiment site. The range in the y−axis of each panel corresponds to +/− 1.0 standard deviations from the study population mean of the relevant log−transformed biomaker unless marked differently. The range in the x−axis of each panel corresponds to +/− 2.0 standard deviations from the study population mean BMI. NMR denotes nuclear magnetic resonance; VLDL, very low density lipoprotein; IDL, intermediate density lipoprotein; LDL, low density liporpotein; HDL, high density lipoprotein; FA, fatty acids; BMI, body−mass index; WC, waist cirfumference; WHR, waist−hip ratio; HC hip circumference.

Figure S3. NMR biomarkers by waist circumference levels before and after adjustment for HC and BMI

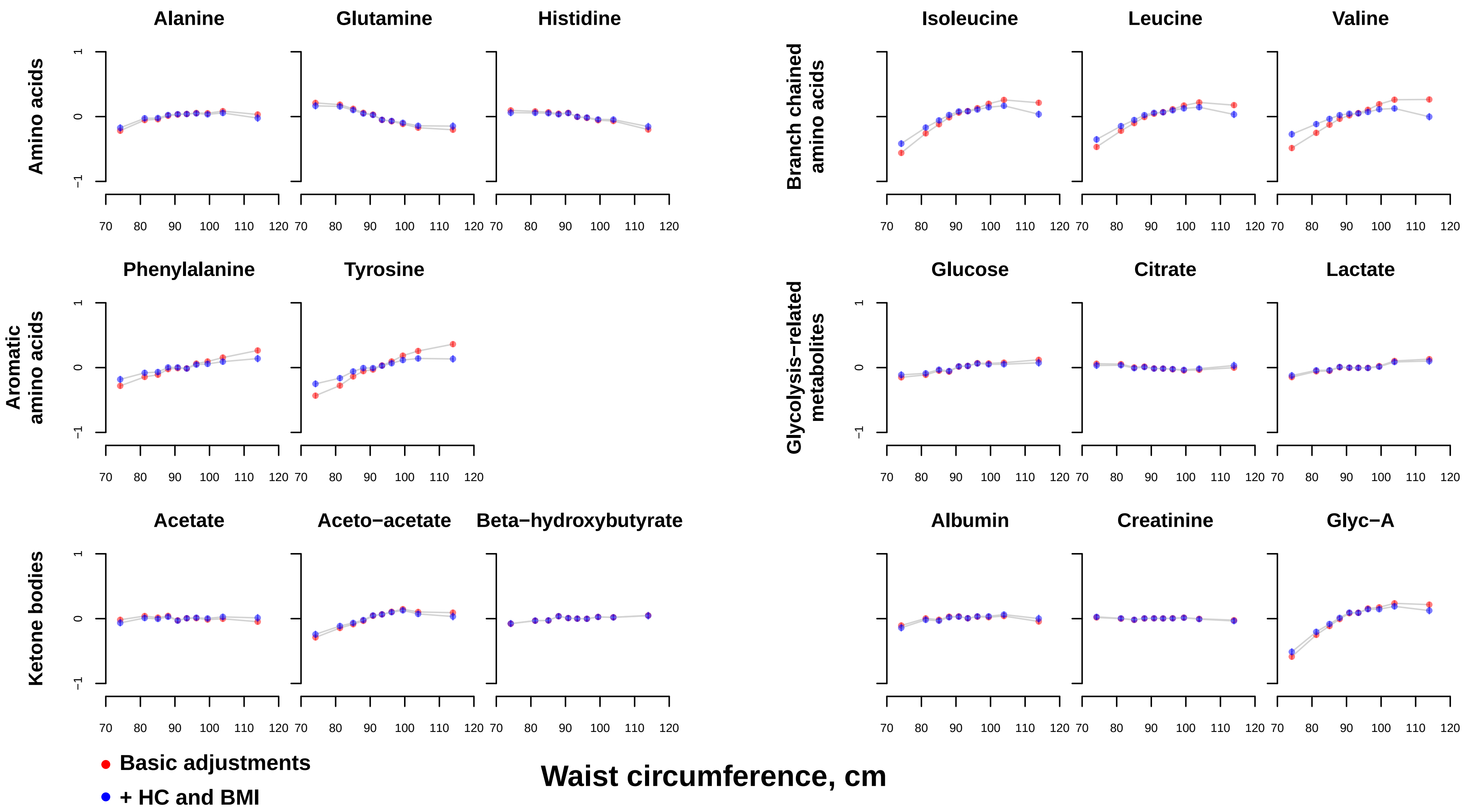

Points represent means (with 95% confidence intervals) of log−transformed (and then scaled to a mean of 0 and an SD of 1) NMR−biomarkers for each tenth of waist circumference. Exclusions as per Figure 2. Estimates are adjusted for age, district of residence, educational level, smoking, alcohol intake, sex−specific fifths of fasting duration, and NMR−experiment site. The range in the y−axis of each panel corresponds to +/− 1.0 standard deviations from the study population mean of the relevant log−transformed biomaker unless marked differently. The range in the x−axis of each panel corresponds to +/− 2.0 standard deviations from the study population mean BMI. NMR denotes nuclear magnetic resonance; VLDL, very low density lipoprotein; IDL, intermediate density lipoprotein; LDL, low density liporpotein; HDL, high density lipoprotein; FA, fatty acids; BMI, body−mass index; WC, waist cirfumference; WHR, waist−hip ratio; HC hip circumference.

Figure S4. NMR biomarkers by hip circumference levels before and after adjustment for WC and BMI

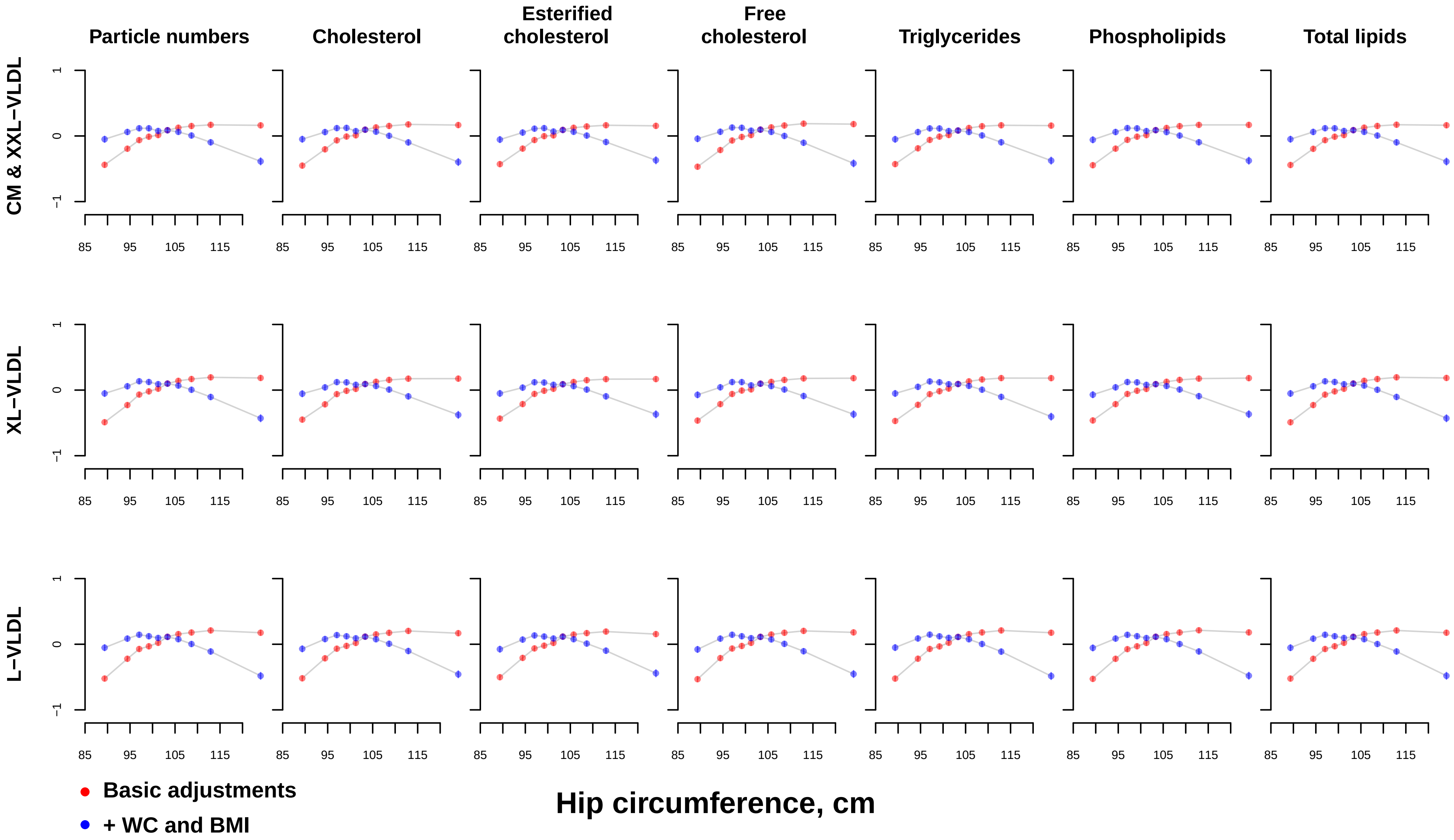

Points represent means (with 95% confidence intervals) of log−transformed (and then scaled to a mean of 0 and an SD of 1) NMR−biomarkers for each tenth of hip circumference. Exclusions as per Figure 2. Estimates are adjusted for age, district of residence, educational level, smoking, alcohol intake, sex−specific fifths of fasting duration, and NMR−experiment site. The range in the y−axis of each panel corresponds to +/− 1.0 standard deviations from the study population mean of the relevant log−transformed biomaker unless marked differently. The range in the x−axis of each panel corresponds to +/− 2.0 standard deviations from the study population mean BMI. NMR denotes nuclear magnetic resonance; VLDL, very low density lipoprotein; IDL, intermediate density lipoprotein; LDL, low density liporpotein; HDL, high density lipoprotein; FA, fatty acids; BMI, body−mass index; WC, waist cirfumference; WHR, waist−hip ratio; HC hip circumference.

Figure S4. NMR biomarkers by hip circumference levels before and after adjustment for WC and BMI

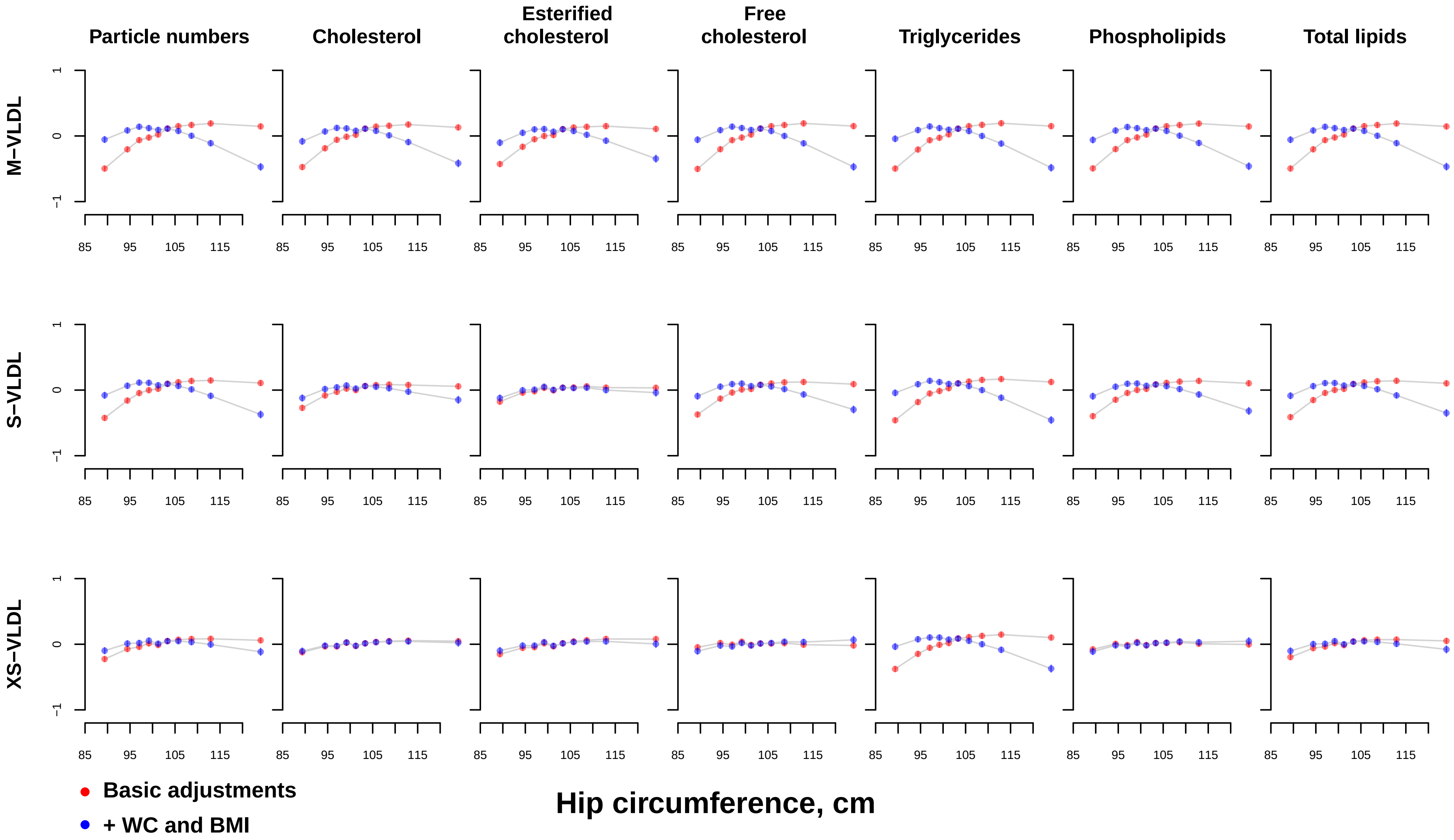

Points represent means (with 95% confidence intervals) of log−transformed (and then scaled to a mean of 0 and an SD of 1) NMR−biomarkers for each tenth of hip circumference. Exclusions as per Figure 2. Estimates are adjusted for age, district of residence, educational level, smoking, alcohol intake, sex−specific fifths of fasting duration, and NMR−experiment site. The range in the y−axis of each panel corresponds to +/− 1.0 standard deviations from the study population mean of the relevant log−transformed biomaker unless marked differently. The range in the x−axis of each panel corresponds to +/− 2.0 standard deviations from the study population mean BMI. NMR denotes nuclear magnetic resonance; VLDL, very low density lipoprotein; IDL, intermediate density lipoprotein; LDL, low density liporpotein; HDL, high density lipoprotein; FA, fatty acids; BMI, body−mass index; WC, waist cirfumference; WHR, waist−hip ratio; HC hip circumference.

Figure S4. NMR biomarkers by hip circumference levels before and after adjustment for WC and BMI

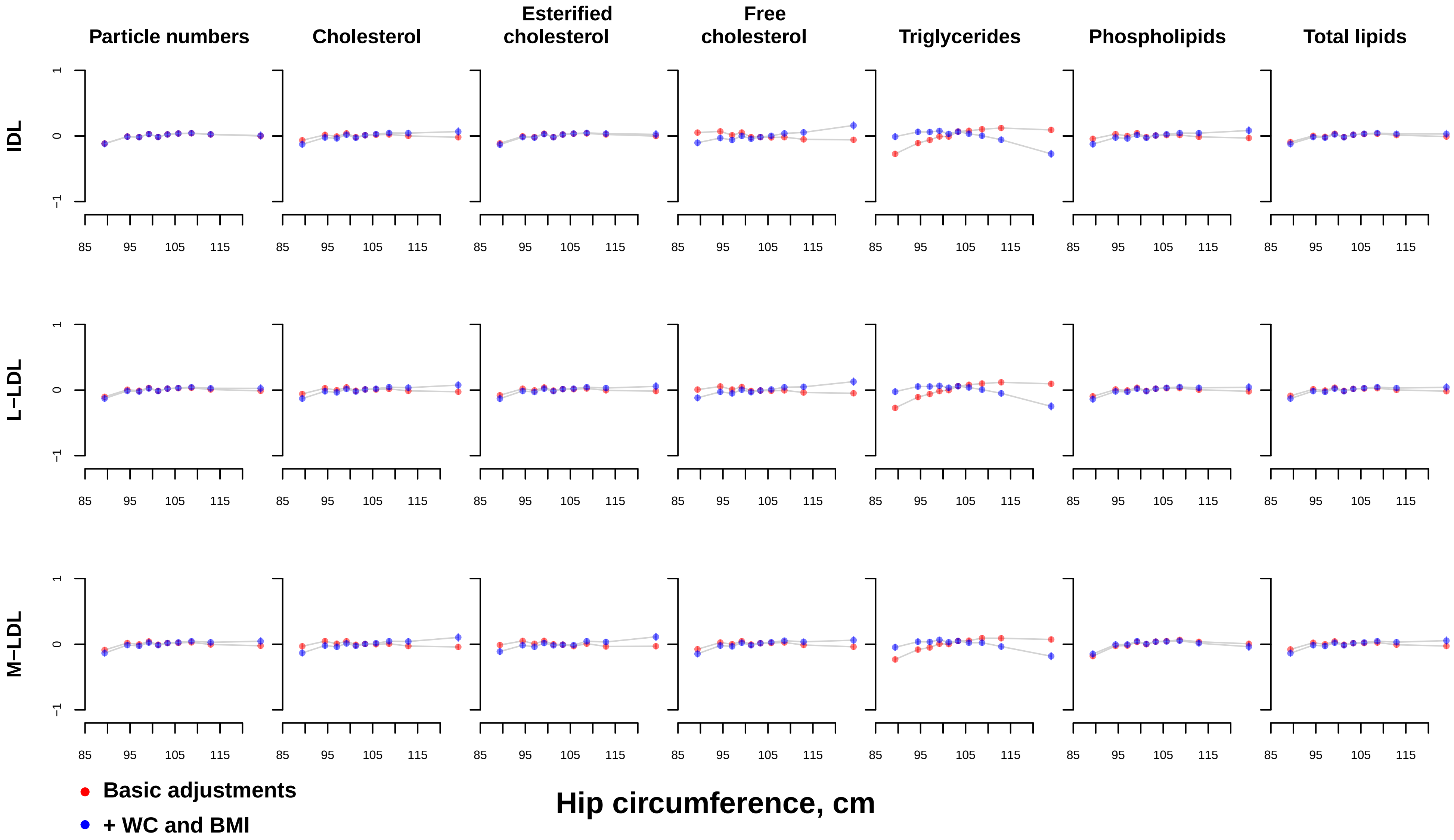

Points represent means (with 95% confidence intervals) of log−transformed (and then scaled to a mean of 0 and an SD of 1) NMR−biomarkers for each tenth of hip circumference. Exclusions as per Figure 2. Estimates are adjusted for age, district of residence, educational level, smoking, alcohol intake, sex−specific fifths of fasting duration, and NMR−experiment site. The range in the y−axis of each panel corresponds to +/− 1.0 standard deviations from the study population mean of the relevant log−transformed biomaker unless marked differently. The range in the x−axis of each panel corresponds to +/− 2.0 standard deviations from the study population mean BMI. NMR denotes nuclear magnetic resonance; VLDL, very low density lipoprotein; IDL, intermediate density lipoprotein; LDL, low density liporpotein; HDL, high density lipoprotein; FA, fatty acids; BMI, body−mass index; WC, waist cirfumference; WHR, waist−hip ratio; HC hip circumference.

Figure S4. NMR biomarkers by hip circumference levels before and after adjustment for WC and BMI

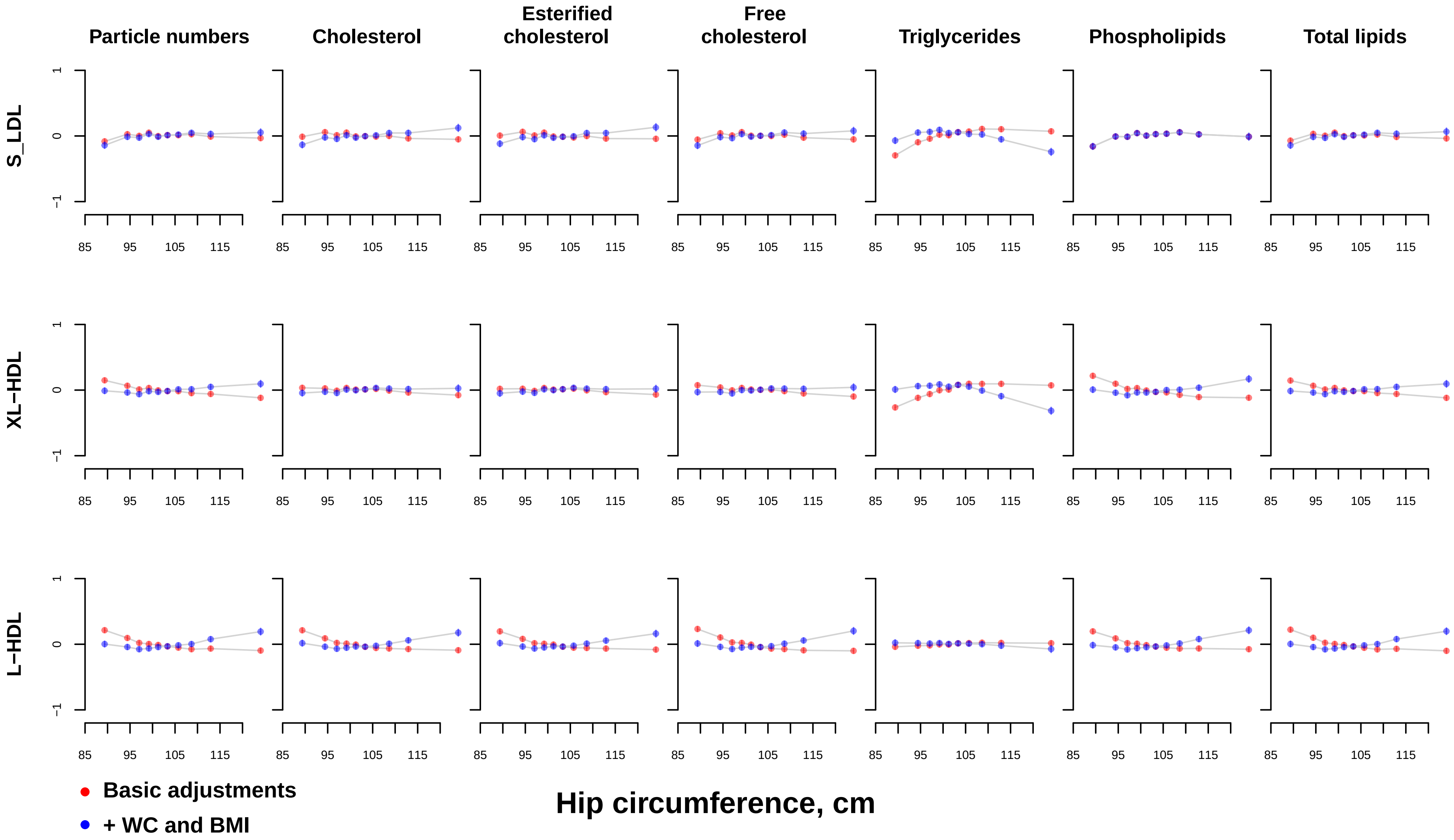

Points represent means (with 95% confidence intervals) of log−transformed (and then scaled to a mean of 0 and an SD of 1) NMR−biomarkers for each tenth of hip circumference. Exclusions as per Figure 2. Estimates are adjusted for age, district of residence, educational level, smoking, alcohol intake, sex−specific fifths of fasting duration, and NMR−experiment site. The range in the y−axis of each panel corresponds to +/− 1.0 standard deviations from the study population mean of the relevant log−transformed biomaker unless marked differently. The range in the x−axis of each panel corresponds to +/− 2.0 standard deviations from the study population mean BMI. NMR denotes nuclear magnetic resonance; VLDL, very low density lipoprotein; IDL, intermediate density lipoprotein; LDL, low density liporpotein; HDL, high density lipoprotein; FA, fatty acids; BMI, body−mass index; WC, waist cirfumference; WHR, waist−hip ratio; HC hip circumference.

Figure S4. NMR biomarkers by hip circumference levels before and after adjustment for WC and BMI

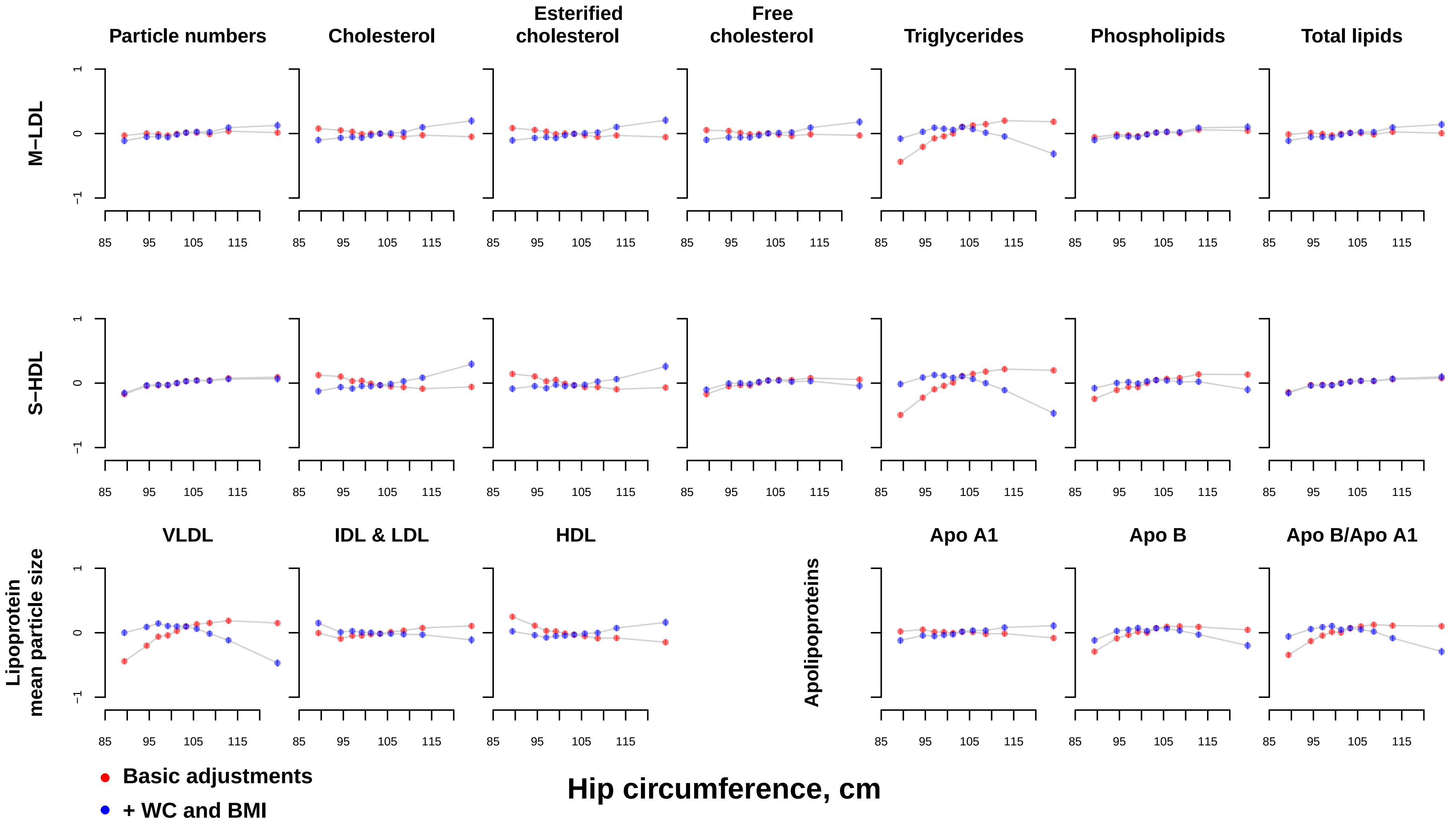

Points represent means (with 95% confidence intervals) of log−transformed (and then scaled to a mean of 0 and an SD of 1) NMR−biomarkers for each tenth of hip circumference. Exclusions as per Figure 2. Estimates are adjusted for age, district of residence, educational level, smoking, alcohol intake, sex−specific fifths of fasting duration, and NMR−experiment site. The range in the y−axis of each panel corresponds to +/− 1.0 standard deviations from the study population mean of the relevant log−transformed biomaker unless marked differently. The range in the x−axis of each panel corresponds to +/− 2.0 standard deviations from the study population mean BMI. NMR denotes nuclear magnetic resonance; VLDL, very low density lipoprotein; IDL, intermediate density lipoprotein; LDL, low density liporpotein; HDL, high density lipoprotein; FA, fatty acids; BMI, body−mass index; WC, waist cirfumference; WHR, waist−hip ratio; HC hip circumference.

Figure S4. NMR biomarkers by hip circumference levels before and after adjustment for WC and BMI

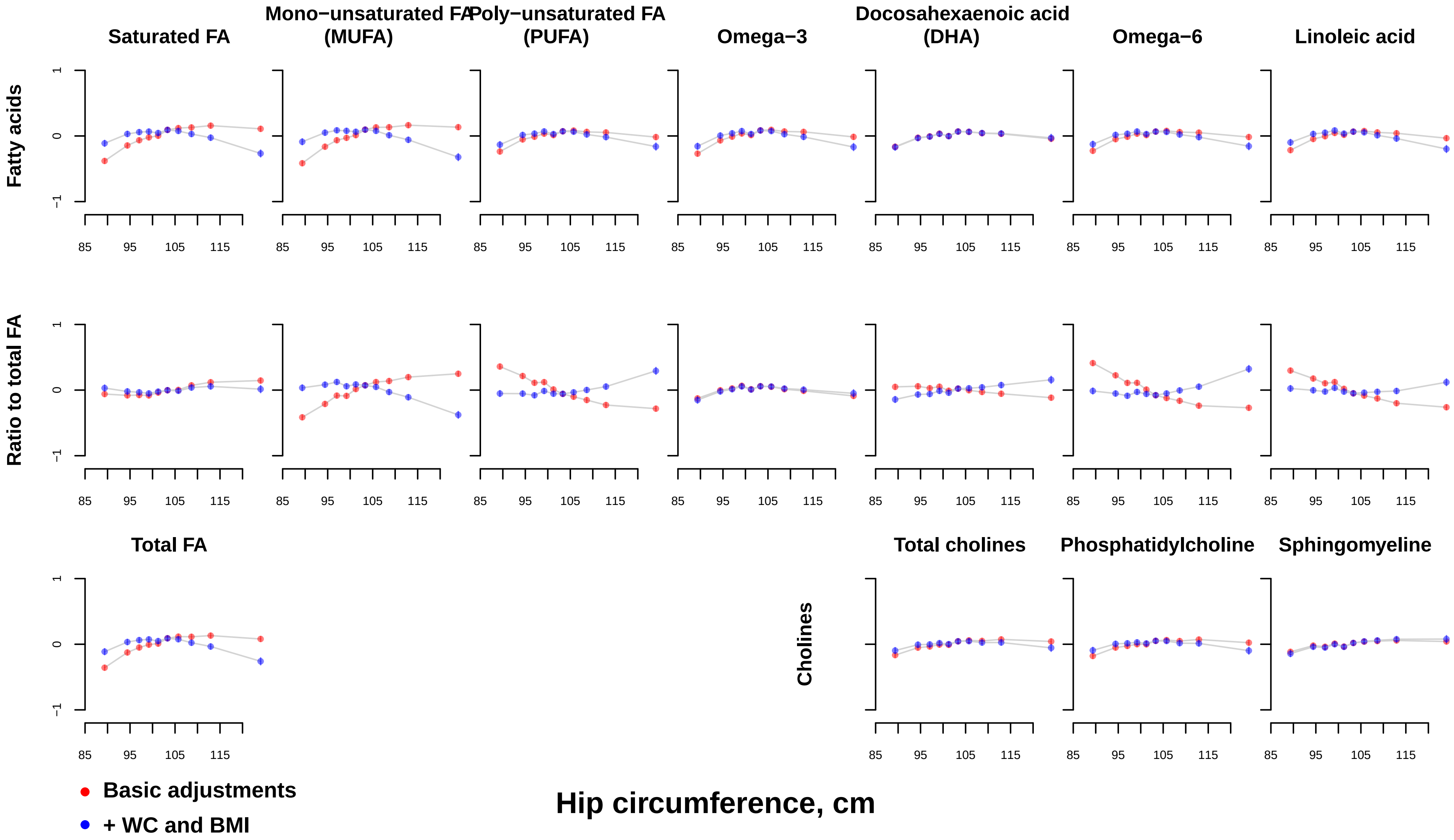

Points represent means (with 95% confidence intervals) of log−transformed (and then scaled to a mean of 0 and an SD of 1) NMR−biomarkers for each tenth of hip circumference. Exclusions as per Figure 2. Estimates are adjusted for age, district of residence, educational level, smoking, alcohol intake, sex−specific fifths of fasting duration, and NMR−experiment site. The range in the y−axis of each panel corresponds to +/− 1.0 standard deviations from the study population mean of the relevant log−transformed biomaker unless marked differently. The range in the x−axis of each panel corresponds to +/− 2.0 standard deviations from the study population mean BMI. NMR denotes nuclear magnetic resonance; VLDL, very low density lipoprotein; IDL, intermediate density lipoprotein; LDL, low density liporpotein; HDL, high density lipoprotein; FA, fatty acids; BMI, body−mass index; WC, waist cirfumference; WHR, waist−hip ratio; HC hip circumference.

Figure S4. NMR biomarkers by hip circumference levels before and after adjustment for WC and BMI

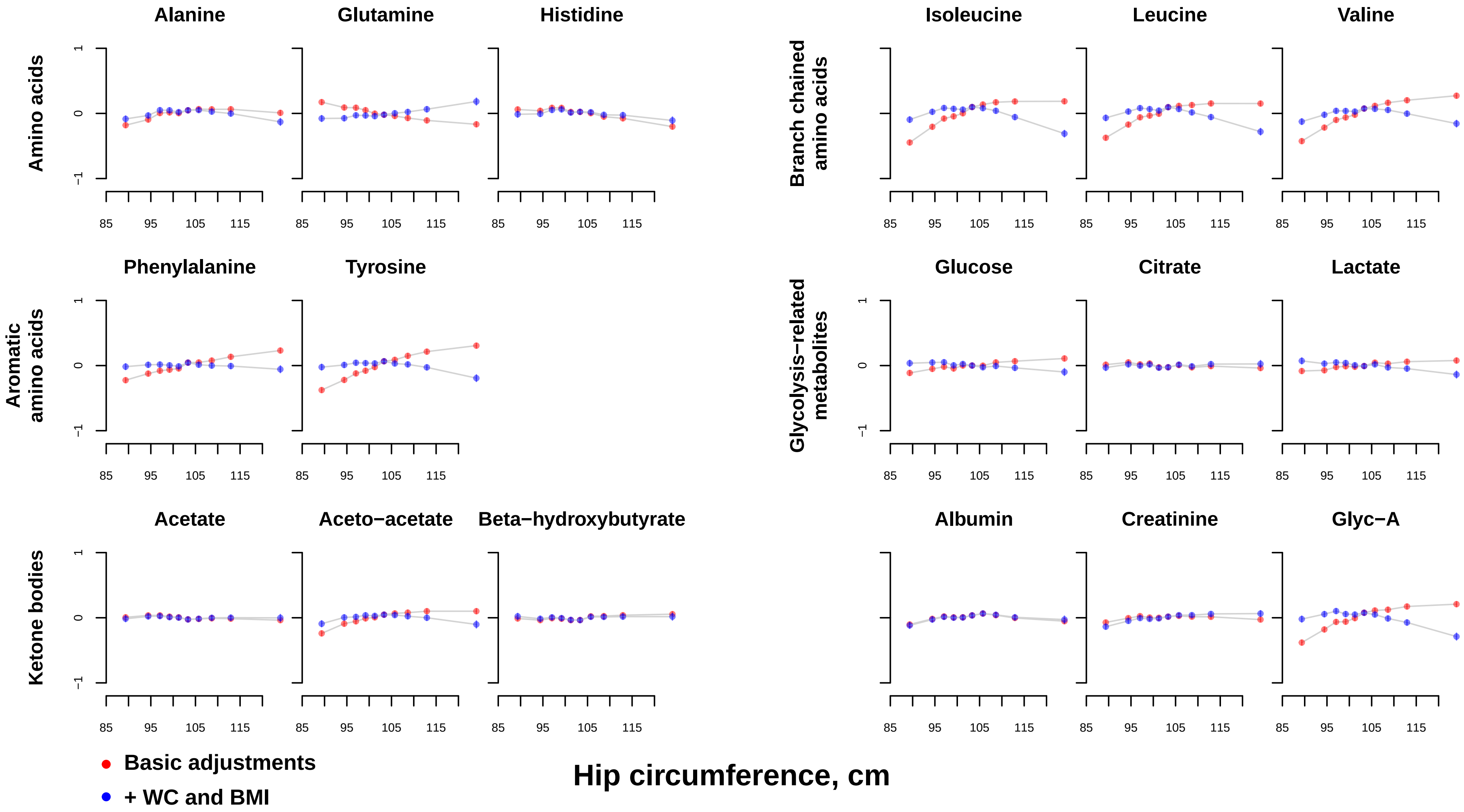

Points represent means (with 95% confidence intervals) of log−transformed (and then scaled to a mean of 0 and an SD of 1) NMR−biomarkers for each tenth of hip circumference. Exclusions as per Figure 2. Estimates are adjusted for age, district of residence, educational level, smoking, alcohol intake, sex−specific fifths of fasting duration, and NMR−experiment site. The range in the y−axis of each panel corresponds to +/− 1.0 standard deviations from the study population mean of the relevant log−transformed biomaker unless marked differently. The range in the x−axis of each panel corresponds to +/− 2.0 standard deviations from the study population mean BMI. NMR denotes nuclear magnetic resonance; VLDL, very low density lipoprotein; IDL, intermediate density lipoprotein; LDL, low density liporpotein; HDL, high density lipoprotein; FA, fatty acids; BMI, body−mass index; WC, waist cirfumference; WHR, waist−hip ratio; HC hip circumference.

Figure S5. Comparison of associations of adiposity measures with each NMR biomarker by age

Difference (in SD−units) of each log−NMR biomarker associated with 2−SD higher adiposity trait in 35−54 years and in 55−84 years

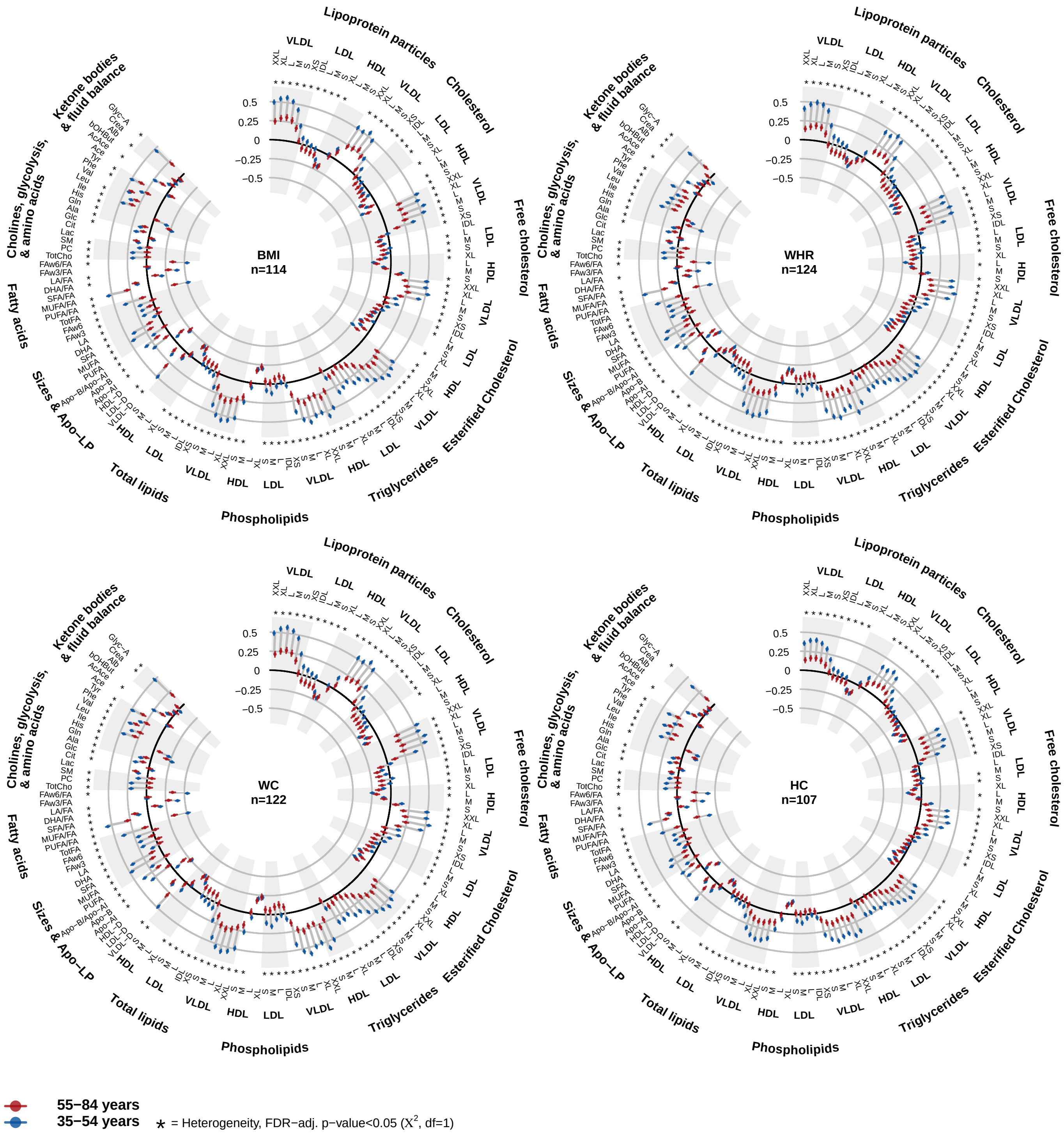

The n reported corresponds to the number of heterogeneity tests by age which would be considered FDR−significant at the 5% level. Exclusions, notation and abbreviations as in Figure 2. All linear regression models are adjusted for, or estimated separately by, age, sex, educational level, district of residence, smoking, alcohol intake, fasting duration, and NMR−experiment site.

Figure S6. Comparison of associations of adiposity measures with each NMR biomarker by sex

Difference (in SD−units) of each log−NMR biomarker associated with 2−SD higher adiposity trait in women and in men

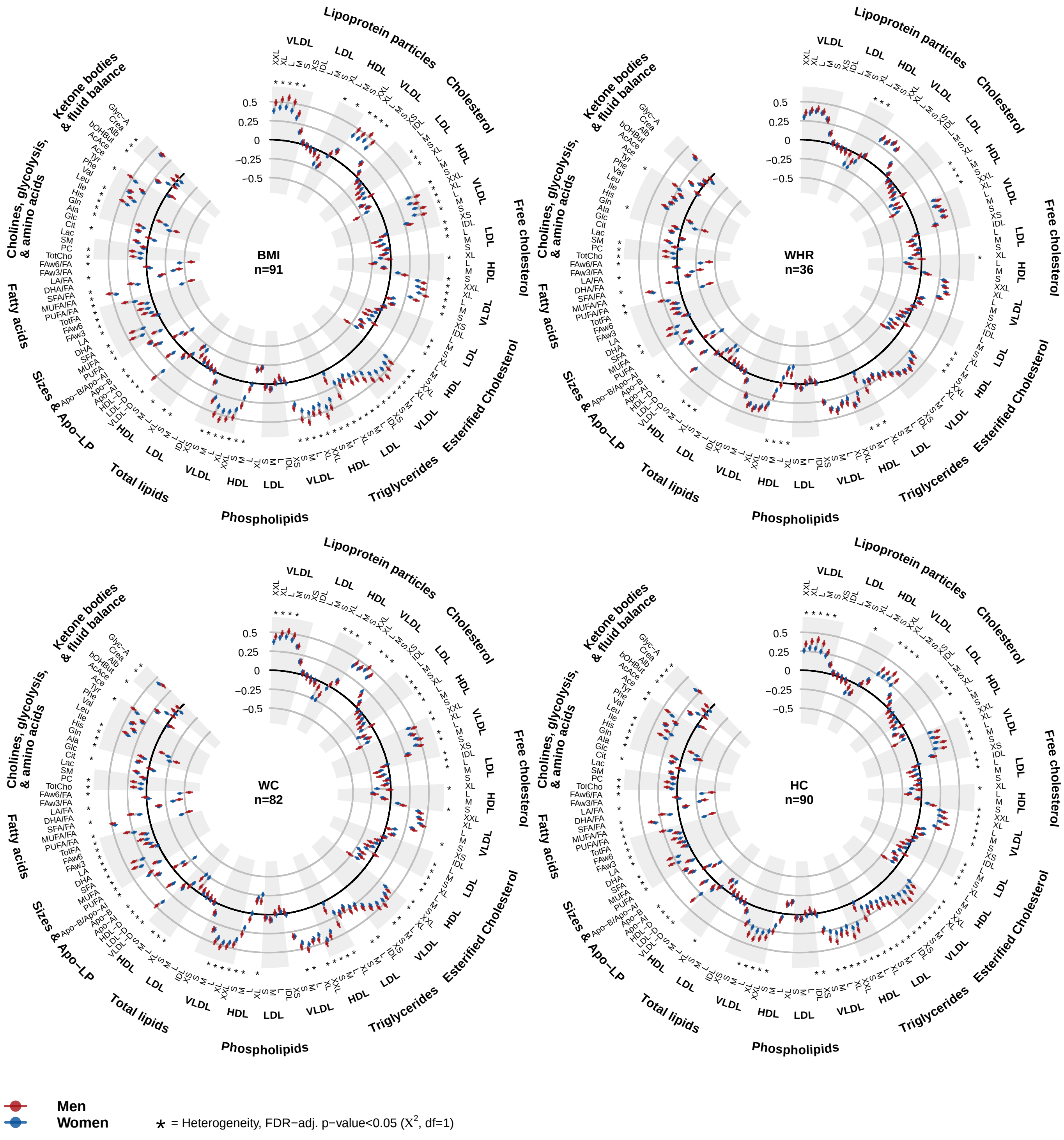

The n reported corresponds to the number of heterogeneity tests by sex which would be considered FDR−significant at the 5% level. Exclusions, notation and abbreviations as in Figure 2. All linear regression models are adjusted for, or estimated separately by, age, sex, educational level, district of residence, smoking, alcohol intake, fasting duration, and NMR−experiment site.

SENSITIVITY ANALYSES − REPEAT OF FIGURE 2 FURTHER INCLUDING PARTICIPANTS WITH PRIOR CHRONIC DISEASE

Figure S7. Associations of adiposity measures with each NMR biomarker

Difference (in SD−units) of each log−NMR biomarker associated with 2−SD higher adiposity measures

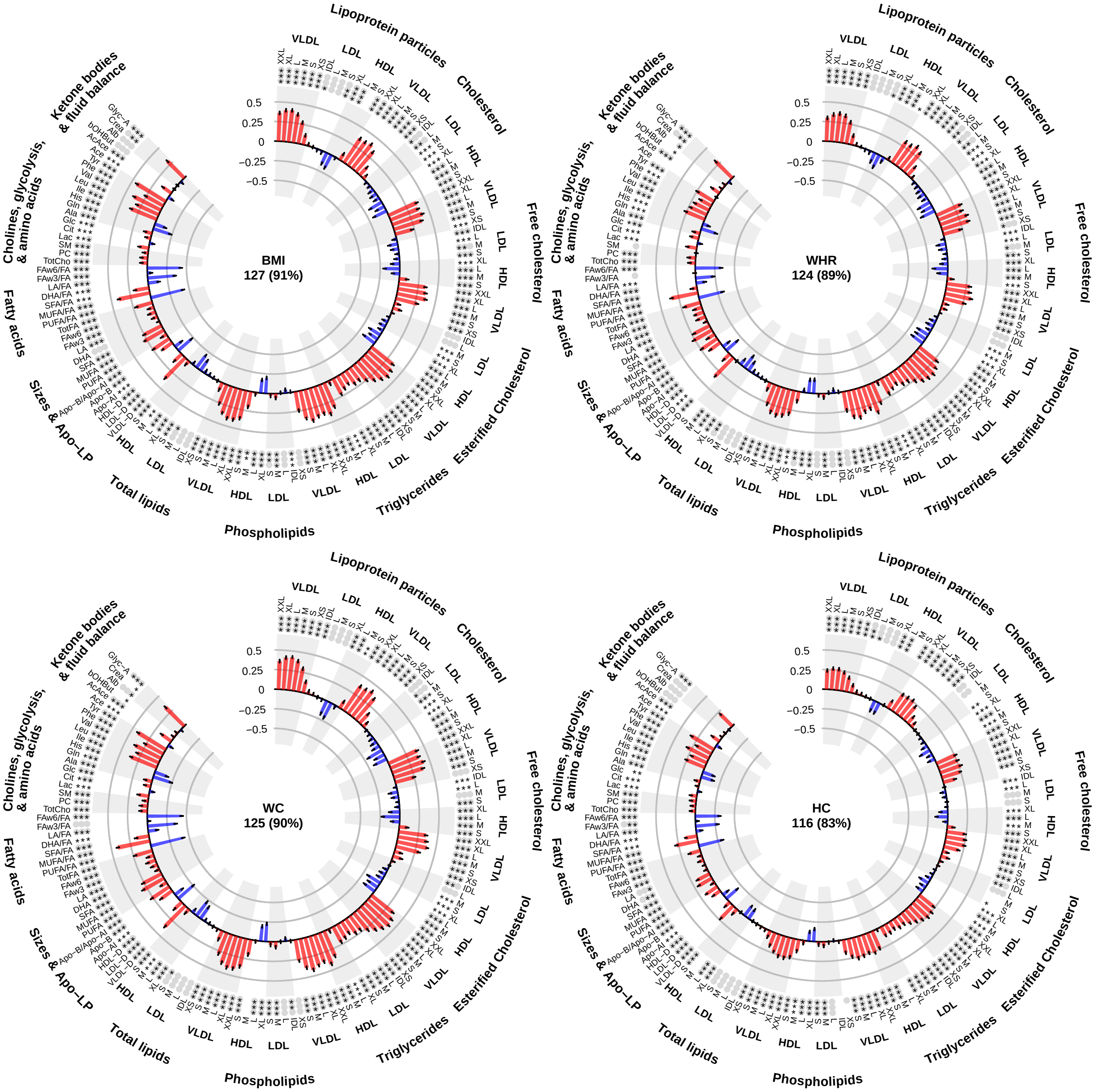

n (%) is the number (%) of the 139 NMR biomarkers that were significantly associated with each adiposity measure after FDR correction.
Analyses included 36,481 participants aged 35 to 84 years who were not using lipid−lowering medications at baseline. All linear regression models are adjusted for age, sex, educational level, district of residence, smoking, alcohol intake, diabetes (i.e., either a self−reported previous diabetes diagnosis, use of glucose−lowering medication, or an HbA1c >6.5%), history of cardiovascular disease (coronary heart disease or stroke), history of other chronic diseases (chronic kidney disease, cirrhosis, emphysema, or cancer), fasting duration, and NMR−experiment site. Notation and abbreviations as in Figure 2.

SENSITIVITY ANALYSES − REPEAT OF FIGURE S5 FURTHER INCLUDING PARTICIPANTS WITH PRIOR CHRONIC DISEASE

Figure S8. Comparison of associations of adiposity measures with each NMR biomarker by age

Difference (in SD−units) of each log−NMR biomarker associated with 2−SD higher adiposity trait in 35−54 years and in 55−84 years

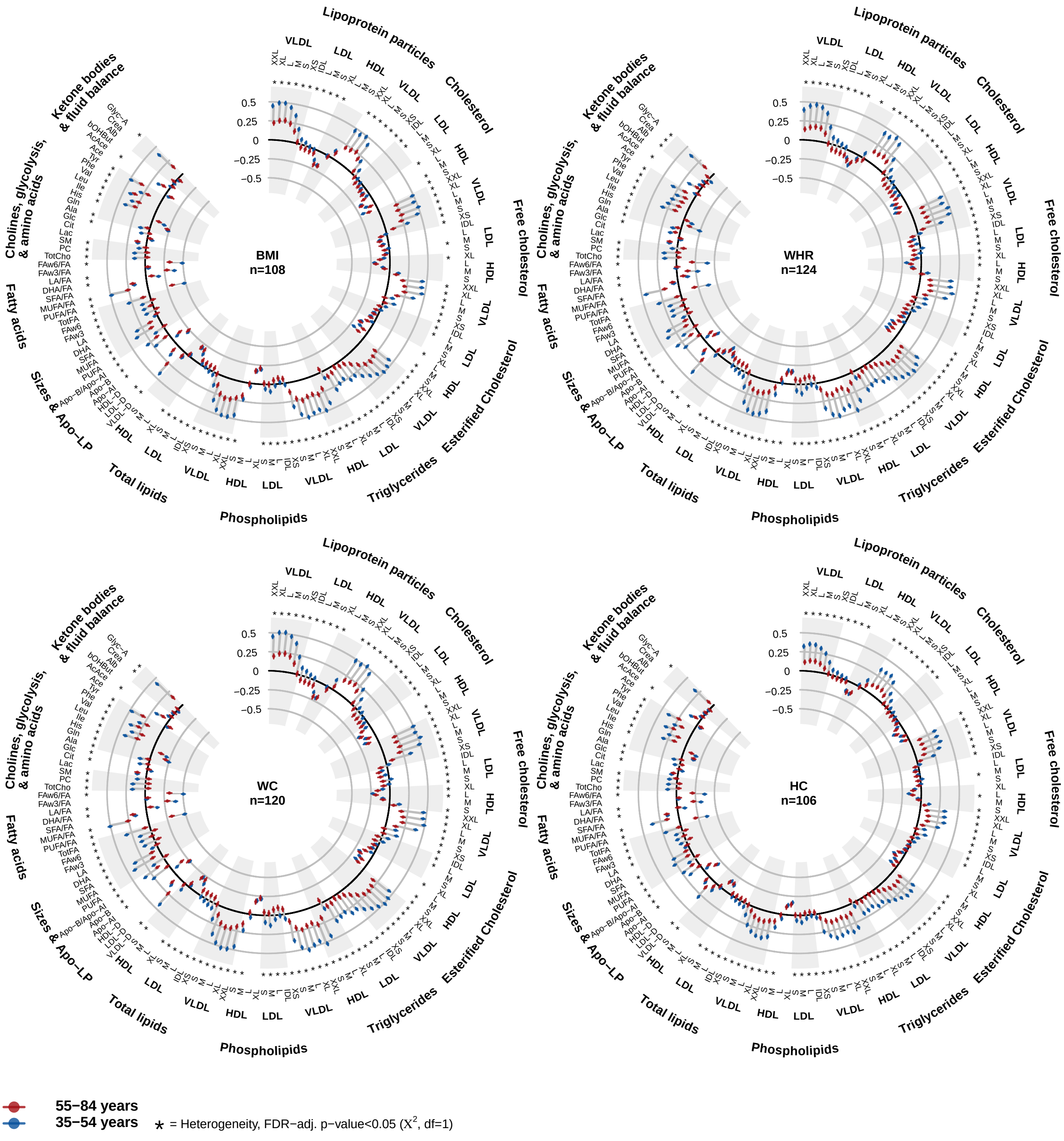

The n reported corresponds to the number of heterogeneity tests by age which would be considered FDR−significant at the 5% level. Analyses included 36,481 participants aged 35 to 84 years who were not using lipid−lowering medications at baseline. All linear regression models are adjusted for, or estimated separately by, age, sex, educational level, district of residence, smoking, alcohol intake, diabetes (i.e., either a self−reported previous diabetes diagnosis, use of glucose−lowering medication, or an HbA1c >6.5%), history of cardiovascular disease (coronary heart disease or stroke), history of other chronic diseases (chronic kidney disease, cirrhosis, emphysema, or cancer), fasting duration, and NMR−experiment site. Notation and abbreviations as per Figure 2.

SENSITIVITY ANALYSES − REPEAT OF FIGURE S6 FURTHER INCLUDING PARTICIPANTS WITH PRIOR CHRONIC DISEASE

Figure S9. Comparison of associations of adiposity measures with each NMR biomarker by sex

Difference (in SD−units) of each log−NMR biomarker associated with 2−SD higher adiposity trait in women and in men

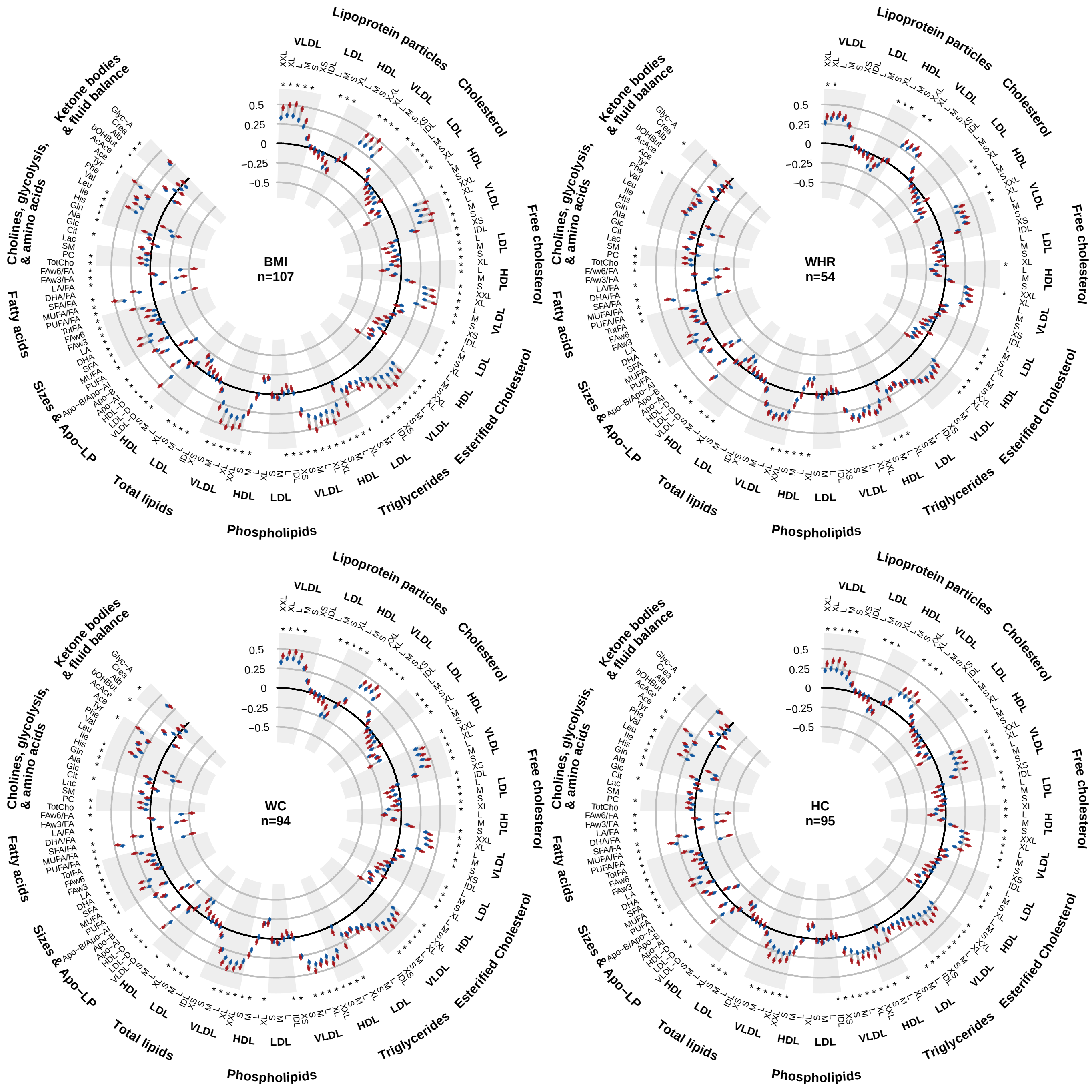

Men
Women

* = Heterogeneity, FDR−adj. p−value<0.05 (X^2, df=1)

The n reported corresponds to the number of heterogeneity tests by sex which would be considered FDR−significant at the 5% level. Analyses included 36,481 participants aged 35 to 84 years who were not using lipid−lowering medications at baseline. All linear regression models are adjusted for, or estimated separately by, age, sex, educational level, district of residence, smoking, alcohol intake, diabetes (i.e., either a self−reported previous diabetes diagnosis, use of glucose−lowering medication, or an HbA1c >6.5%), history of cardiovascular disease (coronary heart disease or stroke), history of other chronic diseases (chronic kidney disease, cirrhosis, emphysema, or cancer), fasting duration, and NMR−experiment site. Notation and abbreviations as per Figure 2.

SENSITIVITY ANALYSES − REPEAT OF FIGURE 3 USING RESIDUALS OF EACH ADIPOSITY MEASURE INSTEAD OF MUTUAL ADJUSTMENTS

Figure S10. Associations of adiposity measures (adjusted for other adiposity measures) with each NMR biomarker

Difference (in SD−units) of each log−NMR biomarker associated with 2−SD higher adiposity measures

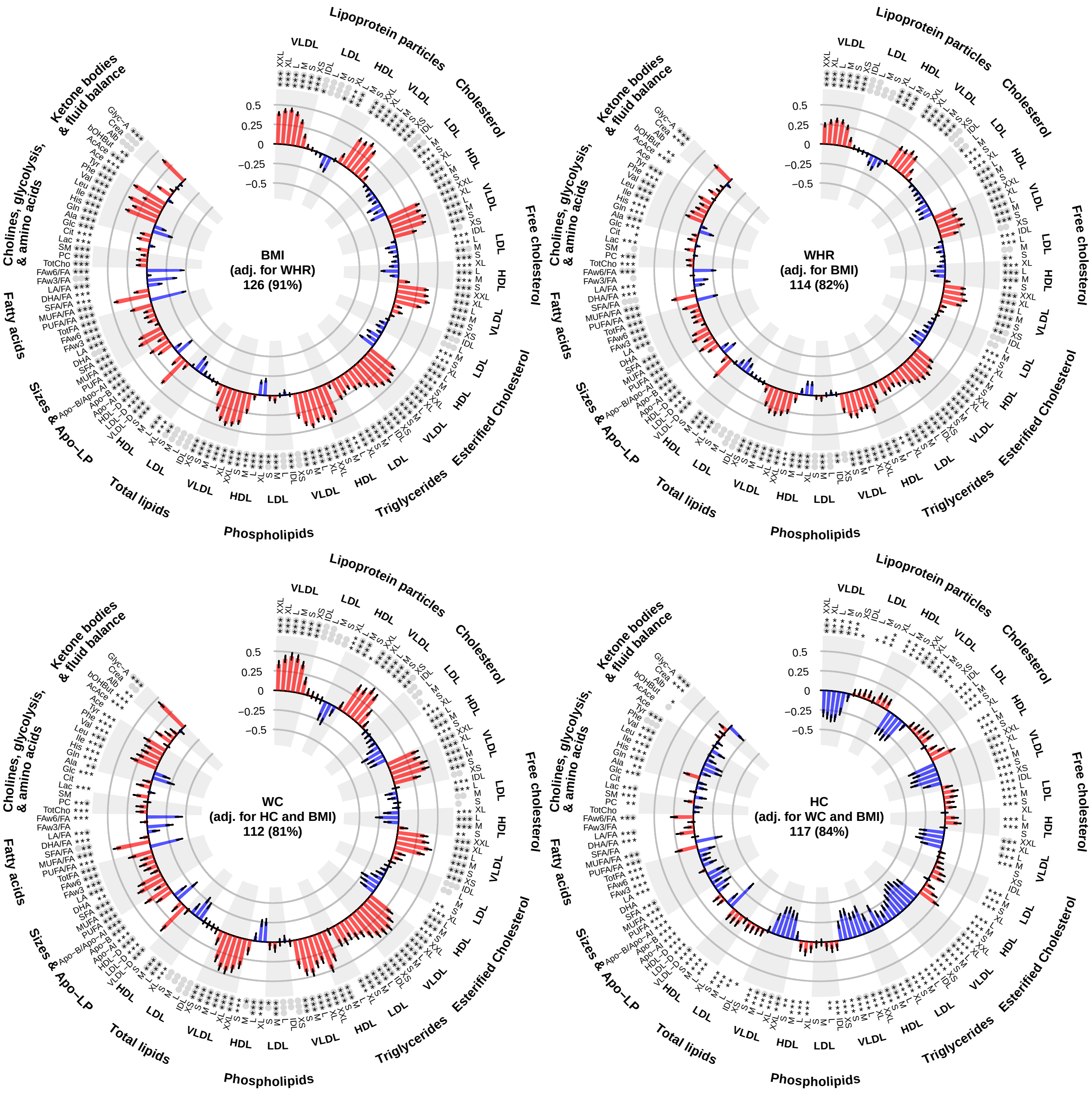

n (%) is the number (%) of the 139 NMR biomarkers that were significantly associated with each adiposity measure after FDR correction.
Analyses, exclusions, notation and abbreviations as in Figure 2

Supplementary methods

Exclusion of participants with prior disease

Certain diseases may induce weight loss (or weight gain) before diagnosis or during the late or severe stages of disease,[s1, s2] which may artificially distort the associations between adiposity and particular NMR biomarkers. In order to reduce this risk, the main analyses excluded those with previous chronic diseases, including those with diagnosed diabetes or HbA1c concentrations consistent with undiagnosed diabetes (as previous findings[s3-s5] from the MCPS strongly suggest that baseline adiposity markers, in particular BMI, were substantially distorted by the presence of pre-existing uncontrolled diabetes). Analyses including those with prior disease are included as sensitivity analyses however.

Selection of NMR biomarkers for main analyses

The Nightingale Health Ltd metabolomics platform provided 228 biomarkers per participant, of which 131 are direct measures and 97 are derived measures. However, some of the direct measures are simple sums of other measures (eg, total lipids within any particular particle subclass equals the sum of free cholesterol, esterified cholesterol, triglycerides and phospholipids). In addition, some are simple ratios of other measures. Consequently, we focussed on a subset of 139 NMR biomarkers that were either relevant direct measures or ratios of particular interest (such as the ratio of apolipoprotein B to apolipoprotein A1, or the ratios of certain fatty acids).

Supplementary references

s1. The Million Women Study Collaborators, Canoy D, Cairns BJ, Balkwill A, Wright FL, Green J, et al. Body mass index and incident coronary heart disease in women: a population-based prospective study. BMC Med. 2013; 11: 87.

s2. D. A. Warrell TMC, J. D. Firth. Oxford Textbook of Medicine. Oxford University Press 2019; Chapters 6.5, 12.6, 21.4 (https://oxfordmedicine.com/view/10.1093/).

s3. Gnatiuc L, Alegre-Díaz J, Wade R, Ramirez-Reyes R, Tapia-Conyer R, Garcilazo-Ávila A, et al. General and Abdominal Adiposity and Mortality in Mexico City: A Prospective Study of 150 000 Adults. Ann Intern Med. 2019; 171: 397.

s4. Alegre-Díaz J, Herrington W, López-Cervantes M, Gnatiuc L, Ramirez R, Hill M, et al. Diabetes and Cause-Specific Mortality in Mexico City. N Engl J Med. 2016; 375:1961–71.

s5. Herrington WG, Alegre-Díaz J, Wade R, Gnatiuc L, Ramirez-Reyes R, Hill M, et al. Effect of diabetes duration and glycaemic control on 14-year cause-specific mortality in Mexican adults: a blood-based prospective cohort study. Lancet Diabetes & Endocrinology 2018; 6: 455–63.
