# Supplementary material for: Adiposity and NMR-measured lipid and metabolic biomarkers among 30,000 Mexican adults
Source: Commun Med (Lond). 2022 Nov 14;2:143. doi: 10.1038/s43856-022-00208-2 (PMC9663185; doi:10.1038/s43856-022-00208-2)
Supplement: Supplementary file 4 — Description of Additional Supplementary Files [file 43856_2022_208_MOESM4_ESM.pdf]

Description of Additional Supplementary Files

File Name: Supplementary Data 1

Description: 139 NMR biomarkers selected for the main analysis

File Name: Supplementary Data 2

Description: Average association of each adiposity marker with each NMR biomarker (for 28,934 participants in the main analysis)
