# Supplementary material for: Adiposity and NMR-measured lipid and metabolic biomarkers among 30,000 Mexican adults
Source: Commun Med (Lond). 2022 Nov 14;2:143. doi: 10.1038/s43856-022-00208-2 (PMC9663185; doi:10.1038/s43856-022-00208-2)
Supplement: Supplementary file 5 — Reporting Summary [file 43856_2022_208_MOESM5_ESM.pdf]

nature portfolio

Corresponding author(s): Jonathan Emberson

Last updated by author(s): Oct 3, 2022

Reporting Summary

Nature Portfolio wishes to improve the reproducibility of the work that we publish. This form provides structure for consistency and transparency in reporting. For further information on Nature Portfolio policies, see our Editorial Policies and the Editorial Policy Checklist.

Statistics

For all statistical analyses, confirm that the following items are present in the figure legend, table legend, main text, or Methods section.

n/a	Confirmed	
☐	☒	The exact sample size (*n*) for each experimental group/condition, given as a discrete number and unit of measurement
☐	☒	A statement on whether measurements were taken from distinct samples or whether the same sample was measured repeatedly
☐	☒	The statistical test(s) used AND whether they are one- or two-sided<br>*Only common tests should be described solely by name; describe more complex techniques in the Methods section.*
☐	☒	A description of all covariates tested
☐	☒	A description of any assumptions or corrections, such as tests of normality and adjustment for multiple comparisons
☐	☒	A full description of the statistical parameters including central tendency (e.g. means) or other basic estimates (e.g. regression coefficient) AND variation (e.g. standard deviation) or associated estimates of uncertainty (e.g. confidence intervals)
☐	☒	For null hypothesis testing, the test statistic (e.g. *F*, *t*, *r*) with confidence intervals, effect sizes, degrees of freedom and *P* value noted<br>*Give P values as exact values whenever suitable.*
☒	☐	For Bayesian analysis, information on the choice of priors and Markov chain Monte Carlo settings
☒	☐	For hierarchical and complex designs, identification of the appropriate level for tests and full reporting of outcomes
☐	☒	Estimates of effect sizes (e.g. Cohen's *d*, Pearson's *r*), indicating how they were calculated

Our web collection on statistics for biologists contains articles on many of the points above.

Software and code

Policy information about availability of computer code

Data collection	We did not use any specific software when collecting the data (other than storing the data in custom databases).
Data analysis	As stated in the manuscript, data processing and statistical analyses were performed in SAS 9.4 (SAS Institute, Cary NC). Plots were created in basic R (v4.0.2) and with the package "RCircos".

For manuscripts utilizing custom algorithms or software that are central to the research but not yet described in published literature, software must be made available to editors and reviewers. We strongly encourage code deposition in a community repository (e.g. GitHub). See the Nature Portfolio guidelines for submitting code & software for further information.

Data

Policy information about availability of data

All manuscripts must include a data availability statement. This statement should provide the following information, where applicable:

- Accession codes, unique identifiers, or web links for publicly available datasets
- A description of any restrictions on data availability
- For clinical datasets or third party data, please ensure that the statement adheres to our policy

The MCPS represents a long-standing collaboration between researchers at the National Autonomous University of Mexico (UNAM) and the University of Oxford. The investigators welcome requests from researchers in Mexico and elsewhere who wish to access MCPS data. If you are interested in obtaining data from the study for research purposes, or in collaborating with MCPS investigators on a specific research proposal, please visit https://www.ctsu.ox.ac.uk/research/

prospective-blood-based-study-of-150-000-individuals-in-mexico where you can download the study's Data and Sample Access Policy in English or Spanish. The policy lists the data available for sharing with researchers in Mexico and in other parts of the world. Full details of the data available may also be viewed at https://datashare.ndph.ox.ac.uk/. The NMR data used in the current report was made available to researchers from Mexico in October 2022. Source data for the figures can be found in Supplementary Data 2.

Human research participants

Policy information about studies involving human research participants and Sex and Gender in Research.

Reporting on sex and gender	When participants were recruited (in 1998-2004), data on self-reported sex was collected using a standardised baseline questionnaire, with the available options being male or female. Throughout the manuscript, the terms 'men' and 'women' are used when referring to self-identified 'males' or 'females' as per the baseline questionnaire. The main analyses focused on epidemiological associations of all the selected participants. However, self-reported sex-specific results are presented and, where appropriate, discussed.
Population characteristics	Individuals are a subset (see 'data exclusions' section in earlier answer) of participants from two districts of Mexico City who were aged 35 years or older when recruited into the Mexico City Prospective Study.
Recruitment	Between 1998 and 2004, 52,644 men and 107,111 women aged 35 years or older who resided in two contiguous districts in Mexico City (Coyoacán and Iztapalapa) were recruited into the Mexico City Prospective Study. Trained health professionals visited participants in their homes and collected information on sociodemographic and lifestyle characteristics, medical history, and current medications using standardised questionnaires.
Ethics oversight	All participants provided written informed consent. Ethics approval was granted by the Mexican Ministry of Health, the Mexican National Council of Science and Technology, and the University of Oxford.

Note that full information on the approval of the study protocol must also be provided in the manuscript.

Field-specific reporting

Please select the one below that is the best fit for your research. If you are not sure, read the appropriate sections before making your selection.

☒ Life sciences ☐ Behavioural & social sciences ☐ Ecological, evolutionary & environmental sciences

For a reference copy of the document with all sections, see nature.com/documents/nr-reporting-summary-flat.pdf

Life sciences study design

All studies must disclose on these points even when the disclosure is negative.

Sample size	NMR metabolomic assays were done in a random sample of 40,000 participants, of which 28,934 were included in the current epidemiological analyses.
Data exclusions	In order to explore the relationships between adiposity and NMR reliably, individuals were excluded if they: were aged ≥85 years at recruitment; had a prior history of diabetes or other chronic disease; had HbA1c ≥6.5%; were taking a lipid-lowering treatment at recuitment; had missing or extreme data on adiposity, covariates or NMR biomarker data; or, for a very small percentage, had been recruited twice (in which case only data from the first visit at which a blood sample was collected was retained). These exclusions are described fully in our manuscript.
Replication	We did not attempt to replicate our findings in a second epidemiological cohort. Our study represents one of the largest such studies to date however (and, to our knowledge, the first large study in a Mexican population).
Randomization	In this observational study, associations were adjusted for age, sex, educational level, district of residence, smoking status, alcohol intake, fifths of fasting duration, and NMR sample processing site.
Blinding	In this observational study no between-group blinding was relevant. However, NMR assays were done blinded to any knowledge of the measured adiposity levels of the individuals who provided the blood samples.

Reporting for specific materials, systems and methods

We require information from authors about some types of materials, experimental systems and methods used in many studies. Here, indicate whether each material, system or method listed is relevant to your study. If you are not sure if a list item applies to your research, read the appropriate section before selecting a response.

Materials & experimental systems

n/a	Involved in the study
☒	☐ Antibodies
☒	☐ Eukaryotic cell lines
☒	☐ Palaeontology and archaeology
☒	☐ Animals and other organisms
☒	☐ Clinical data
☒	☐ Dual use research of concern

Methods

n/a	Involved in the study
☒	☐ ChIP-seq
☒	☐ Flow cytometry
☒	☐ MRI-based neuroimaging
